# Randomized Clinical Trial: Bergamot Citrus and Wild Cardoon Reduce Liver Steatosis and Body Weight in Non-diabetic Individuals Aged Over 50 Years

**DOI:** 10.3389/fendo.2020.00494

**Published:** 2020-08-11

**Authors:** Yvelise Ferro, Tiziana Montalcini, Elisa Mazza, Daniela Foti, Elvira Angotti, Micaela Gliozzi, Saverio Nucera, Sara Paone, Ezio Bombardelli, Ilaria Aversa, Vincenzo Musolino, Vincenzo Mollace, Arturo Pujia

**Affiliations:** ^1^Department of Health Science, University Magna Grecia, Catanzaro, Italy; ^2^Department of Clinical and Experimental Medicine, University Magna Grecia, Catanzaro, Italy; ^3^Department of Medical and Surgical Science, University Magna Grecia, Catanzaro, Italy; ^4^Plantexresearch Srl, Milan, Italy

**Keywords:** nutraceuticals, liver steatosis, lipid, flavonoids, liver elastography

## Abstract

**Background:** Non-alcoholic fatty liver disease is the most common cause of liver-related morbidity and mortality in the world. However, no effective pharmacological treatment for this condition has been found.

**Purpose:** This study evaluated the effect of a nutraceutical containing bioactive components from Bergamot citrus and wild cardoon as a treatment for individuals with fatty liver disease. The primary outcome measure was the change in liver fat content.

**Methods:** A total of 102 patients with liver steatosis were enrolled in a double-blind placebo controlled clinical trial. The intervention group received a nutraceutical containing a Bergamot polyphenol fraction and *Cynara Cardunculus* extract, 300 mg/day for 12 weeks. The control group received a placebo daily. Liver fat content, by transient elastography, serum transaminases, lipids and glucose were measured at the baseline and the end of the study.

**Results:** We found a greater liver fat content reduction in the participants taking the nutraceutical rather than placebo (−48.2 ± 39 vs. −26.9 ± 43 dB/m, *p* = 0.02); The percentage CAP score reduction was statistically significant in those with android obesity, overweight/obesity as well as in women. However, after adjustment for weight change, the percentage CAP score reduction was statistically significant only in those over 50 years (44 vs. 78% in placebo and nutraceutical, respectively, *p* = 0.007).

**Conclusions:** This specific nutraceutical containing bioactive components from Bergamot and wild cardoon reduced the liver fat content during 12 weeks in individuals with liver steatosis over 50 years. If confirmed, this nutraceutical could become the cornerstone treatment of patients affected by liver steatosis.

**Clinical Trial Registration:** www.isrctn.com, identifier ISRCTN12833814.

## Introduction

Non-alcoholic fatty liver disease (NAFLD) is becoming the leading cause of liver damage in Western countries (1). NAFLD is a serious concern because the incidence of hepatocellular carcinoma associated with NAFLD has increased 10-fold in the past decades (2). Despite the genetic heri-tability predisposition to progressive NAFLD (3), the factors leading to the progression from simple liver steatosis to severe liver scarring and cirrhosis are not fully clear. Several studies have shown that inflammatory markers, such as Tumor Necrosis Factor alpha (TNF-α) and other cytokines, play an important role in the pathogenesis of the NAFLD (4). Oxidative stress is one of the key mediators of hepatic damage (5). Additional factors involved in the pathogenesis of NAFLD are obesity and obesity-related metabolic disorders (6, 7). Furthermore, it has been demonstrated that an impaired lipophagy can lead to excessive tissue lipid accumulation making this pathway a new potential therapeutic target in NAFLD (8).

There are currently no approved drugs to treat patients with fatty liver disease. The cornerstone of NAFLD treatment is lifestyle intervention and weight loss (9). Although most of the data on this issue are inconclusive, many dietary natural compounds isolated from fruits and vegetables have been proposed as promising agents capable of reversing hepatic steatosis (10). It has been demonstrated that Bergamot (*Citrus bergamia Risso et Poiteau*) flavonoids, in the form of Bergamot polyphenol fraction (BPF) supplementation, are able to stimulate lipophagy and prevent pathogenic fat accumulation in diet-induced NAFLD rats (11). Hesperidin, one of these flavonoids, prevents several form of liver damage (12, 13) as well as hepatocarcinogenesis (14).

However, molecules that have choleretic properties could offer additional benefits in the treatment of liver steatosis. Wild cardoon (*Cynara cardunculus L.*, the wild ancestor of the globe artichoke) extracts are rich in sesquiterpenes such as cynaropicrin, which possess significant choleretic proprieties thereby improving liver function (15). Bergamot polyphenols do not seem to have a choleretic effect. Wild cardoon also possess anti-inflammatory proprieties (16–18).

Since currently we have no single ingredients working efficiently in NAFLD, the synergic effect of several natural molecules, which possess different proprieties, would represents an original approach in reducing the liver fat content.

In this study our aim was to test the effect of a new nutraceutical containing natural bioactive components from Bergamot and wild Cardoon, as a treatment for patients with liver steatosis.

## Materials and Methods

### Subjects

We enrolled adult individuals invited by newspaper advertisements to be screened for the possible presence of liver steatosis by transient elastography (TE). A population of 102 subjects with NAFLD, aged between 30 and 75 and attending the Clinical Nutrition Unit of the “Mater Domini” Azienda Uni-versity Hospital in Catanzaro, Italy, were enrolled (study duration between February 11, 2019 and June 24, 2019), who were not taking nutraceuticals, supplements or functional food. Local ethical committee at the “Mater Domini” Azienda University Hospital approved the protocol (219/2018/CE, approved September 24, 2018) which was funded by Italian Ministry of University and Research (MIUR, Nutramed Project, PON 03PE000_78_1). The study is listed on the ISRCTN registry (study ID ISRCTN12833814).

According to the protocol of the study, we excluded subjects with past and current alcohol abuse [> 20 g of alcohol per day; 350 mL (12 oz) of beer, 120 mL (4 oz) of wine, and 45 mL (1.5 oz) of hard liquor each contain 10 g of alcohol], who had clinical and laboratory signs of chronic hepatitis B and/or C virus infection or allergies to cardoon, artichoke or maize or with triglycerides concentration over 250 mg/dl (19) and subjects affected by diabetes. Furthermore, we excluded individuals with autoimmune or cholestatic liver disease, liver cirrhosis, pregnancy, nephrotic syndrome, chronic renal failure, gastroesophageal reflux, cancer, and those taking amiodarone, antiretroviral agents, corticosteroids, methotrexate, tamoxifen, valproate, as ascertained from their clinical records. The study's protocol allowed to enroll only long-term lipid-lowering drugs users (more than 6 weeks).

### Study Design

**Figure 1:**
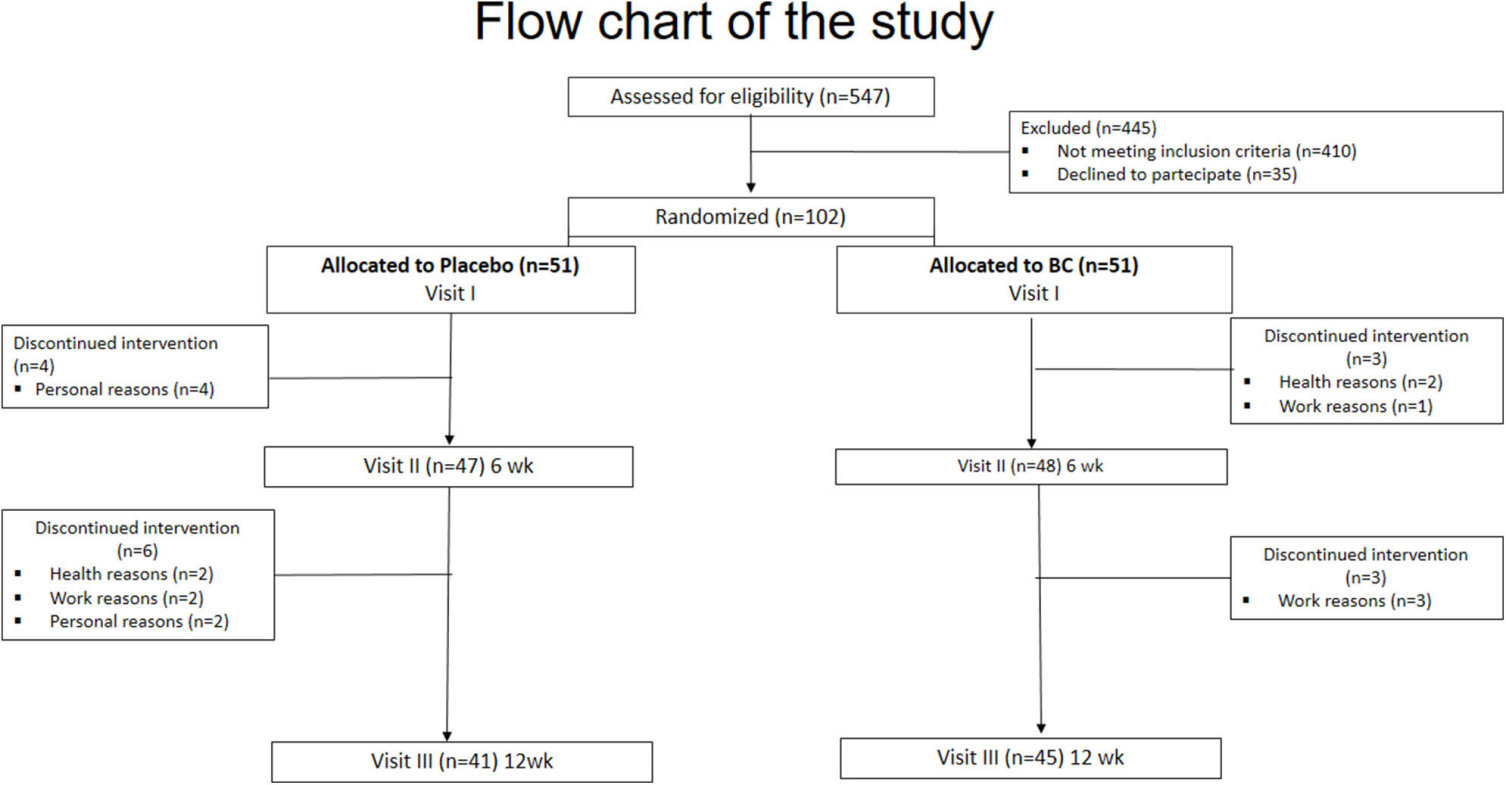
Flow-chart of the study.

Patients were randomly assigned in a 1:1 ratio to receive either a nutraceutical from Bergamot and wild cardoon (abbreviated, BC) or a placebo for up to 12 weeks (Figure 1- Flow-chart of the study). Computer-generated random numbers were used for the simple randomization of subjects.

Eighty-six subjects completed the entire 12 weeks of the study. The study's treatments were as follows:

1. BC (provided by Herbal & Antioxidant SRL, Bianco, RC, Italy): one capsule containing a combination product containing bergamot polyphenolic fraction (BPF®), and wild type *Cynara Cardunculus* extract (CyC) plus excipients including PUFA and a mixture of bergamot pulp and albedo derivative]. (registered Patents RM2008A000615, PCT/IB2009/055061 and 102017000040866); (batch number 18R049, expiration date 10/2020).
2. Placebo: one capsule containing maltodextrin plus excipients including PUFA and a mixture of bergamot pulp and albedo derivative; (batch number 18R050, expiration date 10/2020).

In this study, the primary outcome measure was the change in liver fat content, measured as “controlled attenuation parameter” (CAP) by TE (Fibroscan), and/or other markers of fatty liver, after 12 weeks of treatment. Secondary outcomes were the changes from baseline in plasma lipids and inflammatory markers after 12 weeks of treatment.

Participants received oral and written recommendations to adhere to a Mediterranean dietary pattern, without energy restriction (except in the obese) by a registered dietitians (RD) (20). Y.F. enrolled the participants. A RD assigned participants to interventions. Both the experimenters and participants were blind to who received the nutraceutical or the placebo.

In order to create a negative energy balance, a 400–500 calorie restriction from baseline energy intake was prescribed for overweight/obese individuals.

Written informed consent was obtained from all participants. The investigation conforms to the principles outlined in the Declaration of Helsinki.

The product BC has been carried out according to EU Directive 2004/9/EC and Directive 2004/9/EC for Good Laboratory Practice Guidelines (GLP) as well as OECD Guidelines for Repeated Dose 28 and 90-day Oral Toxicity Study in Rodents.

### Preparation of BC Formulation

The specification sheet with the most relevant active ingredients of BPF and CyC are reported in the supplementary material (Supplementary Figures 1, 2). Briefly, bergamot juice (BJ) was obtained from peeled-off fruits by industrial pressing and squeezing. The juice was oil fraction depleted by stripping, clarified by ultra-filtration and loaded on suitable polystyrene resin columns absorbing polyphenol compounds of MW between 300 and 600 Da (Mitsubishi). Polyphenol fraction was eluted by a mild KOH solution. Moreover, the phytocomplex was then neutralized by filtration on cationic resin at acidic pH. Finally, it was vacuum dried and minced to the desired particle size to obtain a powder. In particular, powder was micronized and co-grinded with bergamot albedo fibers when given alone or in combination. BPF powder was analyzed by HPLC for flavonoid and other polyphenol content. In addition, toxicological analyses were performed including heavy metal, pesticide, phthalate and sinephrine content which revealed the absence of known toxic compounds. Standard microbiological tests detected no mycotoxins and bacteria. The same procedure was used for the production of CyC extract. Fibers obtained by bergamot albedo were used micronized and co-grinded with plant extracts as excipients for final formulations. All materials were provided by HEAD srl (Bianco, Italy.) Finally, to obtain a formulation containing both extracts, 150 mg of BPF powder were combined with 150 mg of CyC and were encapsulated in capsules containing 300 mg of excipients represented by albedo fibers micronized and co-grinded with plant extracts (Seris srl, Cuneo, Italy). The final formulation contained 5% of cynaropicrin. Capsules containing 600 mg maltodextrin were prepared for placebo studies. All capsules were put into bottles containing 56 capsules (for 4 week) and packaged in color-coded plastic bags containing 4 bottles (for 16 weeks) for distribution to participants. All procedures have been performed according to the European Community Guidelines concerning dietary supplements (for toxicological reports and pharmacokinetic studies see Supplemental Material).

## Dietary Intake Assessment (see Supplemental Tables 1, 2)

### Liver Transient Elastography (TE)

CAP is a novel non-invasive measure of NAFLD. TE can quantify liver steatosis by CAP assessment and measure liver stiffness (Fibroscan® Echosense SASU, Paris, France) (21). Both CAP and stiffness score were obtained simultaneously and in the same volume of liver parenchyma. All patients were evaluated using the 3.5 MHz standard M probe on the right lobe of the liver through intercostal spaces with the patient lying supine and placing the right arm behind the head to facilitate access to the right upper quadrant of the abdomen. The tip of the probe transducer was placed on the skin between the rib bones at the level of the right lobe of the liver. All scans were performed by the same investigator. Liver stiffness was expressed by the median value (in kPa) of 10 measurements performed between 25 and 65 mm depth. Only results with 10 valid shots and interquartile range (IQR)/median liver stiffness ratio<30% were included. The cut-off value for defining the presence of fibrosis was liver stiffness >7 kPa.

We assessed CAP score using only the M probe because the CAP algorithm is specific to this device. Ten successful measurements were performed on each patient, and only cases with 10 successful acquisitions were taken into account for this study. The success rate was calculated as the number of successful measurements divided by the total number of measurements. The ratio of the IQR of liver stiffness to the median (IQR/MLSM) was calculated as an indicator of variability. The final CAP score (ranged from 100 to 400 decibels per meter (dBm–), was the median of individual measurements. The ratio of IQR in CAP values to the median (IQR/M CAP) was used as an indicator of variability for the final CAP. The diagnosis of NAFLD was based on a CAP > 216 dB/m. In order to identify each steatosis grade, three different cut-offs were used: CAP between 216 and 252 dB/m for the diagnosis of S1 grade, CAP between 253 and 296 dB/m for the diagnosis of S2 grade, and CAP > 296 dB/m for the diagnosis of S3 grade (severe) (22, 23).

### Anthropometric Measurements and Cardiovascular Risk Factors Assessment

Body weight, BMI and waist and hip circumferences (WC and HC) were measured as previously described (20). Obesity was defined by the presence of a body mass index (BMI) ≥ 30 kg/m^2^.

We assessed the presence of the classical cardiovascular (CV) risk factors from clinical records and patient interview (20, 24, 25). Blood pressure was determined at the time of the three visits (20). The following criteria were used to define and exclude diabetes: fasting blood glucose ≥ 126 mg/dL or antidiabetic treatment (20). Metabolic Syndrome (MS) definition was based on the National Cholesterol Education Program's (NCEP) Adult Treatment Panel III report (ATP III). Individuals with at least three or more abnormalities were identified as having MS (26).

According to World Health Organization criteria (27), android obesity was defined as a Waist-hip-ratio (WHR) above 0.90 for males and above 0.85 for females.

### Biochemical Evaluation

Venous blood was collected after fasting overnight into vacutainer tubes (Becton & Dickinson, Plymouth, England) and centrifuged within 4 h. Serum glucose, total cholesterol, high density lipoprotein (HDL)-cholesterol, triglycerides, creatinine, ALT, AST, GGT, and insulin were measured by chemiluminescent immunoassay on COBAS 8000 (Roche, Switzerland), according to the manufacturer's instructions. LDL- C level was calculated by the Friedewald formula (28).

Blood count analysis was performed using ADVIA 2120i (Siemens Healthcare Diagnostics, Marburg, Germany).

Homeostatic model assessment (HOMA) index was calculated for assessing β-cell function and insulin resistance (IR) from fasting glucose and insulin concentrations (29). The serum concentrations of interleukin-1β (IL-1β), interleukin-6 (IL-6), and tumor necrosis factor α (TNF-α) were determined by sandwich enzyme-linked immunosorbent assay (ELISA) (R&D Systems Inc., Minneapolis, USA) according to the manufacturer's instructions.

### Safety Parameters and Adverse Events

We measured several parameters of general health such as blood pressure as well as serum glucose, creatinine, total bilirubin, and lipids.

We used a patient-reported outcome questionnaire for the measurement of adverse events (AEs). The questionnaire investigated the presence of any new symptoms after entering the study that could be related to the intervention. We assessed the nature of the ADs such as those regarding severity.

### Data Analysis

Data are reported as mean ± standard deviation (SD).

It was taken into account a mean CAP value of 268.6 ± 52 dB/m for individuals with liver steatosis (30). Thus, to detect a CAP score reduction of at least 12%, with an effect size (ES = mean CAP difference/ baseline SD) of 0.62, with 80% power on a two-sided level of significance of 0.05, a minimum of 44 subjects for each group were required. Considering a 10% of drop-out, we enrolled 102 patients (Figure 1).

A Chi square test was performed to analyze the prevalence between groups and an independent unpaired samples *t*-test was used to compare the difference between means. Specifically, we calculated the changes in variables and compared the means of these changes between treatment groups. Changes in the clinical characteristics from baseline to follow-up (within group variation) were calculated using paired Student's *t*-test (two tailed). We used the Bonferroni adjustment method for multiple testing correction (General Linear Model -GLM). Indeed, a repeated measure ANOVA was performed to evaluate time-group difference in CAP score (ANOVA/GLM for grouped data).

We used both an indirect assessment method (i.e., pill count) and patient interviews to assess adherence. We performed intention-to-treat (ITT) as well as on-treatment (OT) analyses, defining OT as those participants taking more than 80% of the prescribed treatment.

We performed several *post-hoc* analyses (not pre-specified) in the following subgroups of participants: ITT; OT; overweight/obese; men; women; with metabolic syndrome; over 50 years; with age ≤ 50 years; with gynoid obesity; with android obesity.

Significant differences were assumed to be present at *p* < 0.05 (two-tailed). All comparisons were performed using SPSS 22.0 for Windows (IBM Corporation, New York, NY, United States).

## Results

Ninety-five subjects completed the first part of the study. Four subjects were lost within 6 weeks in the placebo group and three subjects in the BC group (one due to suspected cancer and one due to low back pain in BC; Figure 1). Eighty-six subjects completed the study (Figure 1). One participant was lost due to suspected cancer and one due to aphthous oral ulcer in the placebo group). The mean age of the population was 51 ± 9 years. A total of 52 (61%) were male. The mean basal LDL-C and CAP score was 119 ± 33 mg/dl and 289 ± 39 dB/m, respectively.

### Dietary Intake Assessment

Nutrient intake assessment and dietary intake changes during the study are showed in Supplemental Table 2.

### Baseline Demographic and Clinical Characteristics of Participants According to the Treatments

Table 1 shows the basal clinical characteristics of participants according to the allocation (*n* = 102). The groups were comparable for all of the characteristics. About half of the population had hyperlipidemia.

**Table 1:** Baseline demographic and clinical characteristics of participants according to the treatments.

**Variables**	**Placebo (*n* = 51)**	**BC (*n* = 51)**	***p-value***
Age (years)	51 ± 11	53 ± 9	0.43
Weight (Kg)	80 ± 11	80 ± 12	0.94
BMI (Kg/m^2^)	29 ± 4	29 ± 3	0.86
WC (cm)	97 ± 8	99 ± 10	0.29
HC (cm)	107 ± 7	105 ± 7	0.30
FM (Kg)	25 ± 8	25 ± 7	0.80
SBP (mmHg)	113 ± 17	112 ± 14	0.97
DBP (mmHg)	71 ± 14	73 ± 11	0.70
CAP score (dB/m)	285 ± 40	294 ± 39	0.27
IQR	11 ± 6	11 ± 5	0.95
Stiffness (kPa)	5.3 ± 1.4	4.9 ± 1.3	0.15
IQR	14 ± 5	14 ± 7	0.53
Glucose (mg/dL)	92 ± 8	93 ± 8	0.74
Insulin (mU/L)	9.9 ± 4	10 ± 5	0.84
HOMA-IR	2.3 ± 1.0	2.3 ± 1.2	0.74
TC (mg/dL)	196 ± 39	193 ± 39	0.66
TG (mg/dL)	115 ± 48	107 ± 53	0.42
HDL-C (mg/dL)	51 ± 13	53 ± 12	0.48
LDL-C (mg/dL)	122 ± 34	118 ± 34	0.60
Non-HDL-C (mg/dL)	145 ± 36	140 ± 39	0.49
AST (IU/L)	23 ± 15	21 ± 6	0.23
ALT (IU/L)	32 ± 27	23 ± 12	0.06
γGT (UI/L)	27 ± 20	25 ± 17	0.59
Creatinine (mg/dL)	0.82 ± 0.1	0.83 ± 0.2	0.80
Uric Acid (mg/dL)	5.1 ± 1.1	5.2 ± 1.2	0.70
Total bilirubin (mg/dL)	0.61 ± 0.3	0.62 ± 0.3	0.90
**Prevalence**			
Gender (Female,%)	45	43	1
Menopause (%)	70	68	1
Smokers (%)	12	24	0.19
Obesity (%)	41	33	0.77
MS (%)	31	22	0.37
Hypertension (%)	35	37	1
Hyperlipidemia (%)	57	49	0.55
Antihypertensive drugs (%)	29	31	1
Lipid-lowering agents (%)	10	18	0.38
Antiplatelet agents (%)	6	10	0.71
Liver steatosis S1 grade (%)	26	16	0.20
Liver steatosis S2 grade (%)	35	35	0.20
Liver steatosis S3 grade (%)	39	49	
Liver Fibrosis (%)	10	6	0.71

*BMI, body mass index; WC, waist circumference; HC, hip circumference; FM, fat mass; SBP, systolic blood pressure; DBP, diastolic blood pressure; CAP, controlled attenuation parameter; IQR, interquartile range; HOMA-IR, homeostatic model assessment of insulin resistance; TC, total cholesterol; TG, triglycerides; HDL-C, high density lipoprotein cholesterol; LDL-C, low density lipoprotein cholesterol; AST, aspartate aminotransferase; ALT, alanine aminotransferase; γGT, gamma glutamyltransferase*.

The nutrient profile of the overall diet of the population according to the treatments is shown in Supplemental Table 1. At enrolment, nutrient intake of the two groups were comparable (Supplemental Table 2). Furthermore, Supplemental Table 2, shows the dietary changes during the study. Only overweight/obese individuals reached a 400–500 caloric restriction from the baseline intake. At the end of the intervention, the two groups were comparable *(t-test)*.

Supplemental Figure 3 shows individual body weight reduction according to the treatment.

### Clinical Characteristics Changes at Follow-Up and Outcome of the Study

Table 2 shows the baseline and follow-up clinical characteristics of participants who completed the study (12 weeks) according to the treatment group (*n* = 86). At baseline, the groups were comparable for all of the characteristics (see *unpaired t-test* between treatments). LDL-C, HDL- C, non-HDL-C, TC, HOMA- IR, AST, and γGT decreased only in the participants taking BC (LDL- C from 116 ± 32 to 107 ± 30 mg/dl, *p* = 0.002, Table 2).

**Table 2:** Baseline and follow-up clinical characteristics of participants according to the treatments (Intention To Treat analysis).

	**Placebo (*****n*** **= 41)**			**BC (*****n*** **= 45)**				
**Variables**	**Basal**	**Follow-up**	***p*-value** **(paired *t*-test)**	**Basal**	**Follow-up**	***p*-value** **(paired *t*-test)**	***p*-value** **(unpaired *t*-test between basal values)**	***p*-value (unpaired *t*-test between follow-up values)**
Weight (Kg)	80 ± 11	77 ± 11	<0.001	80 ± 12	76 ± 12	<0.001	0.87	0.64
BMI (Kg/m^2^)	28.7 ± 4	27.7 ± 4	<0.001	29.2 ± 3	27.6 ± 3	<0.001	0.53	0.76
WC (cm)	97 ± 8	93 ± 8	<0.001	99 ± 10	95 ± 9	<0.001	0.23	0.52
HC (cm)	107 ± 7	103 ± 6	<0.001	106 ± 7	102 ± 7	<0.001	0.52	0.50
FM (Kg)	25 ± 8	22 ± 8	<0.001	25 ± 7	21 ± 7	<0.001	0.93	0.54
SBP (mmHg)	111 ± 17	110 ± 13	0.73	111 ± 14	109 ± 12	0.31	0.97	0.70
DBP (mmHg)	71 ± 15	71 ± 9	0.91	73 ± 11	72 ± 10	0.58	0.48	0.90
CAP score (dB/m)	283 ± 40	256 ± 52	<0.001	295 ± 38	247 ± 42	<0.001	0.15	0.37
Stiffness (kPa)	5.1 ± 1.4	4.6 ± 1.2	0.046	5.0 ± 1.3	4.7 ± 1.2	0.18	0.68	0.74
Glucose (mg/dL)	92 ± 8	92 ± 8	0.95	93 ± 8	92 ± 13	0.85	0.35	0.95
Insulin (mU/L)	10.2 ± 4	8.9 ± 5	0.021	10.2 ± 5	8.3 ± 5	0.016	0.86	0.50
HOMA-IR	2.3 ± 1.1	2.1 ± 1.2	0.07	2.4 ± 1.3	2.0 ± 1.2	0.034	0.76	0.63
TC (mg/dL)	194 ± 40	189 ± 42	0.30	188 ± 34	177 ± 33	0.001	0.48	0.13
TG (mg/dL)	113 ± 48	110 ± 55	0.72	102 ± 47	101 ± 49	0.84	0.28	0.38
HDL-C (mg/dL)	50 ± 12	49 ± 11	0.55	52 ± 12	49 ± 13	0.002	0.38	0.99
LDL-C (mg/dL)	122 ± 35	117 ± 36	0.32	116 ± 32	107 ± 30	0.002	0.42	0.15
Non-HDL-C (mg/dL)	144 ± 38	139 ± 42	0.34	136 ± 36	127 ± 34	0.003	0.32	0.13
AST (IU/L)	21 ± 9	20 ± 5	0.37	21 ± 6	19 ± 4	0.015	0.71	0.76
ALT (IU/L)	28 ± 21	22 ± 9	0.007	24 ± 13	19 ± 8	0.001	0.29	0.14
γGT (UI/L)	27 ± 21	25 ± 22	0.07	26 ± 18	22 ± 18	0.011	0.76	0.56
Creatinine (mg/dL)	0.82 ± 0.1	0.85 ± 0.1	0.034	0.83 ± 0.1	0.83 ± 0.1	0.63	0.68	0.63
Uric Acid (mg/dL)	5.2 ± 1.1	5.3 ± 1.2	0.27	5.2 ± 1.1	5.1 ± 1.0	0.42	1	0.33
Total bilirubin (mg/dL)	0.62 ± 0.3	0.69 ± 0.4	0.046	0.64 ± 0.3	0.65 ± 0.4	0.88	0.82	0.85
WBCs (x10^3^/uL)	6.3 ± 1.3	6.0 ± 1.1	0.06	6.4 ± 1.6	6.0 ± 1.5	0.07	0.91	0.89
Lymphocyte (x10^3^/uL)	1.91 ± 0.4	1.96 ± 0.4	0.58	2.06 ± 0.6	1.98 ± 0.7	0.47	0.31	0.95
Neutrophil (x10^3^/uL)	3.68 ± 1.3	3.37 ± 0.8	0.19	4.01 ± 1.1	3.90 ± 0.9	0.60	0.87	0.027
Monocyte (x10^3^/uL)	0.37 ± 0.1	0.36 ± 0.1	0.80	0.39 ± 0.1	0.38 ± 0.1	0.78	0.75	0.63
***Cytokine evaluation***								
IL-1β (pg/mL)	3.08 ± 0.6	1.53 ± 0.8	<0.001	3.11 ± 0.8	1.75 ± 0.8	<0.001	0.85	0.24
IL-6 (pg/mL)	1.68 ± 0.8	2.57 ± 1.3	<0.001	1.92 ± 0.9	3.03 ± 1.3	<0.001	0.21	0.12
TNF-α (pg/mL)	3.08 ± 0.6	1.53 ± 0.8	<0.001	3.56 ± 3.9	2.04 ± 2.4	0.002	0.55	0.16

*BMI, body mass index; WC, waist circumference; HC, hip circumference; FM, fat mass; SBP, Systolic Blood Pressure; DBP, Diastolic Blood Pressure; CAP, controlled attenuation parameter; HOMA-IR, homeostatic model assessment of insulin resistance; TC, total cholesterol; TG, triglycerides; HDL-C, high density lipoprotein cholesterol; LDL-C, low density lipoprotein cholesterol; AST, aspartate aminotransferase; ALT, alanine aminotransferase; γGT, gamma glutamyltransferase; WBCs, white blood cells; IL-1β, interleukin-1β; IL-6, interleukin-6; TNF-α, tumor necrosis factor α*.

The CAP score, body weight, insulin and ALT decreased in the participants taking BC as well as in participants in the placebo group (in BC group, CAP score dropped from 295 ± 38 to 247 ± 42 dB/m, *p* < 0.001; paired *t*-test; Table 2).

**Figure 2:**
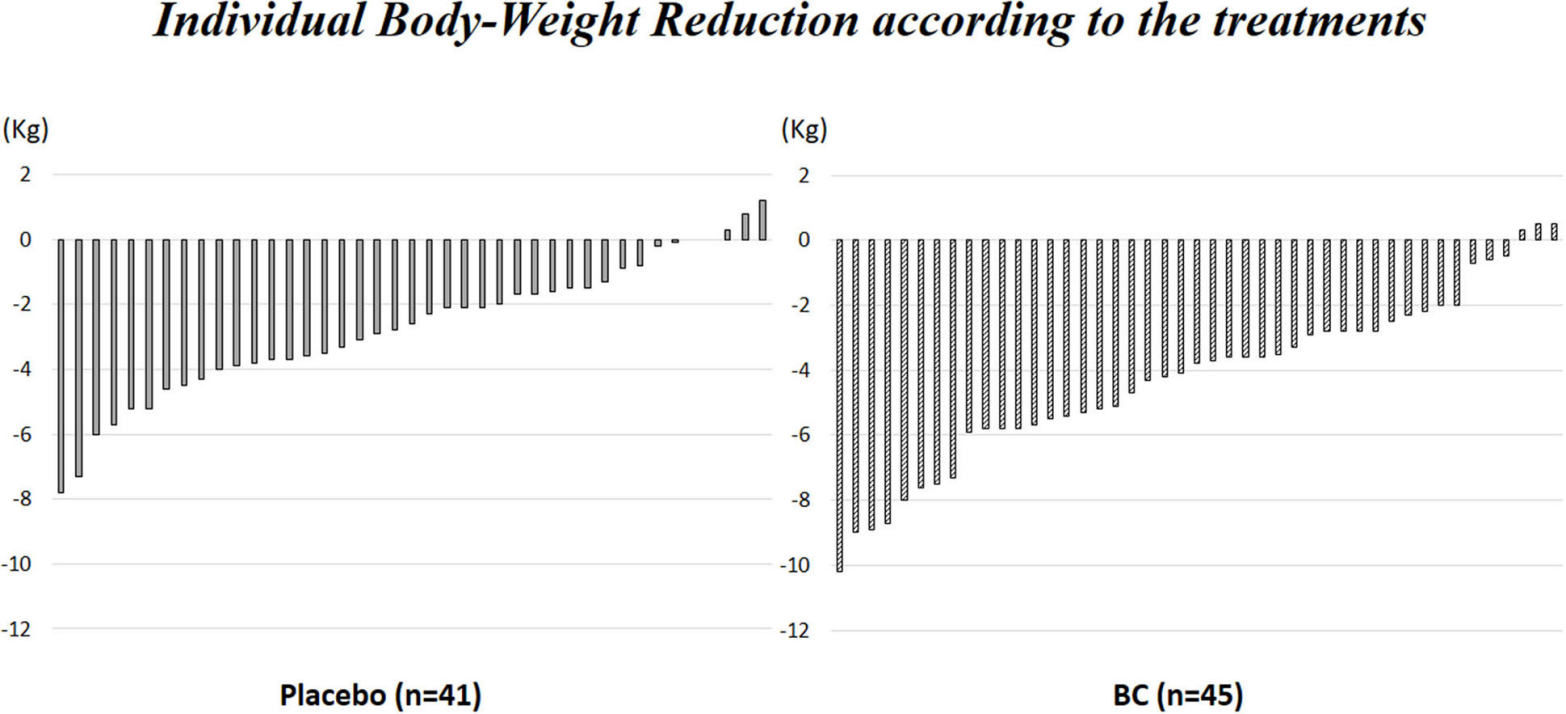
Individual CAP score reduction according to the treatments.

Figure 2 shows the individual CAP score reduction for the participants in each group.

The changes in the clinical parameters after each treatment period are shown in Table 3. The change in the serum concentration of the white blood cells, IL-1β, IL-6, and TNF-α did not differ between groups (Table 3).

**Table 3:** Changes in clinical parameters at follow-up according to the treatments (unpaired *t*-test).

**Variables**	**Placebo (*n* = 41)**	**BC (*n* = 45)**	***p-value***
Follow-up duration (days)	88 ± 4	88 ± 6	0.94
Adherence to treatment (≥ 80%, %)	92	92	1
Weight (Kg)	−2.7 ± 2	−4.2 ± 3	0.004
BMI (Kg/m^2^)	−0.9 ± 0.8	−1.6 ± 0.9	0.003
WC (cm)	−3.7 ± 4	−4.9 ± 4	0.16
HC (cm)	−3.2 ± 3	−3.2 ± 4	0.99
FM (kg)	−2.4 ± 2	−3.3 ± 3	0.07
CAP score (dB/m)	−26.9 ± 43	−48.2 ± 39	0.020
Stiffness (kPa)	−0.46 ± 1.4	−0.26 ± 1.3	0.49
Glucose (mg/dL)	−0.07 ± 7	−0.3 ± 10	0.91
Insulin (mU/L)	−1.2 ± 3	−1.9 ± 5	0.41
HOMA-IR	−0.2 ± 0.8	−0.4 ± 1	0.43
TC (mg/dL)	−5.2 ± 32	−11.9 ± 21	0.26
TG (mg/dL)	−2.2 ± 40	−1.0 ± 34	0.85
HDL-C (mg/dL)	−0.5 ± 6	−2.8 ± 6	0.06
LDL-C (mg/dL)	−4.3 ± 28	−8.9 ± 18	0.36
Non-HDL-C (mg/dL)	−4.3 ± 27	−9.1 ± 19	0.42
AST (IU/L)	−0.9 ± 7	−1.8 ± 5	0.48
ALT (IU/L)	−3.5 ± 28	−2.0 ± 17	0.76
γGT (UI/L)	−2.6 ± 9	−3.8 ± 9	0.55
Uric Acid (mg/dL)	0.13 ± 0.8	−0.09 ± 0.7	0.18
Total bilirubin (mg/dL)	0.07 ± 0.2	0.01 ± 0.3	0.22
WBCs (x 10^3^/uL)	−0.37 ± 1.3	−0.35 ± 1.3	0.94
Lymphocyte (x 10^3^/uL)	0.04 ± 0.4	−0.07 ± 0.5	0.36
Neutrophil (x 10^3^/uL)	−0.31 ± 1.2	−0.11 ± 1.0	0.50
Monocyte (x 10^3^/uL)	−0.01 ± 0.1	−0.01 ± 0.1	0.97
IL-1β (pg/mL)	−1.56 ± 0.8	−1.36 ± 0.9	0.29
IL-6 (pg/mL)	0.42 ± 0.5	0.48 ± 0.4	0.56
TNF-α (pg/mL)	−1.73 ± 2.1	−1.52 ± 2.9	0.71

*BMI, body mass index; WC, waist circumference; HC, hip circumference; FM, fat mass; CAP, controlled attenuation parameter; HOMA-IR, homeostatic model assessment of insulin resistance; TC, total cholesterol; TG, triglycerides; HDL-C, high density lipoprotein cholesterol; LDL-C, low density lipoprotein cholesterol; AST, aspartate aminotransferase; ALT, alanine aminotransferase; γGT, gamma glutamyltransferase; WBCs, white blood cells; IL-1β, interleukin-1β; IL-6, interleukin-6; TNF-α, tumor necrosis factor α*.

BC significantly lowered body weight, BMI and CAP score compared to the placebo [absolute difference: body weight−2.7 ± 2 *vs*. −4.2 ± 3 kg (*p* = 0.004) and CAP score −26.9 ± 43 *vs*. −48.2 ± 39 dB/m (*p* = 0.002) in the placebo and BC group, respectively (Table 3). The mean CAP score decreased significantly over time in the BC group vs. the placebo group (Figure 2).

### Subgroup Analysis

Table 4 shows the changes in the clinical parameters in subgroups according to the treatments. The percentage CAP score reduction was statistically significant in those with android obesity or in overweight/obese individuals (Table 4). Furthermore, CAP score reduction was higher in the OT rather than ITT analysis (−17.1 *vs*. −15.9% in OT *vs*. ITT analysis) as well as a higher CAP score reduction was found in women rather than in men (*p* = 0.03, Table 4). However, after adjustment for weight change, the percentage CAP score reduction was statistically significant only in those over 50 years.

**Table 4:** Changes in clinical parameters in the subgroups according to the treatments.

**Variables**	**Placebo (*n* = 41)**	**BC (*n* = 45)**	***p-value***
**ITT**			
Weight (Kg)	−2.7 ± 2	−4.2 ± 3	0.004
CAP score (dB/m)	−26.9 ± 43	−48.2 ± 39	0.020
CAP (%)	−9.2 ± 16	−15.9 ± 13	0.036
Improvement (%)	51	76	0.025
**On-Treatment**			
Weight (Kg)	−2.7 ± 2	−4.4 ± 3	0.002
CAP score (dB/m)	−25.7 ± 45	−51.7 ± 38	0.007
CAP (%)	−8.7 ± 17	−17.1 ± 12	0.013
Improvement (%)	50	79	0.010
**Women**			
Weight (Kg)	−2.6 ± 2	−4.3 ± 3	0.063
CAP score (dB/m)	−22.3 ± 47	−54.1 ± 35	0.034
CAP (%)	−8 ± 18	−17.8 ± 10	0.067
Improvement (%)	56	83	0.13
**Men**			
Weight (Kg)	−2.8 ± 2	−4.2 ± 3	0.030
CAP score (dB/m)	−29.9 ± 42	−44.3 ± 42	0.225
CAP (%)	−9.9 ± 15	−14.7 ± 14	0.249
Improvement (%)	48	70	0.15
**Age ≤ 50 years**			
Weight (Kg)	−2.9 ± 2	−4.1 ± 3	0.17
CAP score (dB/m)	−43.4 ± 37	−39.5 ± 43	0.75
CAP (%)	−15.7 ± 13	−13.9 ± 15	0.67
Improvement (%)	67	73	0.74
**Age > 50 years**			
Weight (Kg)	−2.4 ± 2	−4.4 ± 2	0.004
CAP score (dB/m)	−9.7 ± 44	−56.5 ± 34	<0.001
*a*CAP score (dB/m)^*^	−13.7 ± 9	−52.9 ± 8	0.004
CAP (%)	−2.3 ± 16	−17.9 ± 10	0.001
Improvement (%)	35	78	0.006
**Android Obesity**			
Weight (Kg)	−2.8 ± 3	−4.4 ± 3	0.016
CAP score (dB/m)	−23.7 ± 45	−51.2 ± 39	0.014
CAP (%)	−8.1 ± 17	−16.8 ± 13	0.027
Improvement (%)	44	80	0.007
**Gynoid Obesity**			
Weight (Kg)	−2.5 ± 3	−3.8 ± 3	0.22
CAP score (dB/m)	−33.3 ± 41	−37.6 ± 41	0.80
CAP (%)	−11.4 ± 16	−12.9 ± 14	0.80
Improvement (%)	64	60	1
**Metabolic Syndrome**			
Weight (Kg)	−2.8 ± 2	−3.4 ± 2	0.43
CAP score (dB/m)	−9.1 ± 54	−47 ± 54	0.13
CAP (%)	−2.3 ± 20	−14.2 ± 16	0.16
Improvement (%)	55	45	0.65
**Overweight & Obesity**			
Weight (Kg)	−3.0 ± 2	−4.5 ± 3	0.005
CAP score (dB/m)	−25.6 ± 46	−50.7 ± 40	0.018
CAP (%)	−8.4 ± 17	−16.5 ± 18	0.031
Improvement (%)	44	78	0.007

*CAP, controlled attenuation parameter*.

* *CAP score adjusted for body weight change*.

### Disease Risk Analysis

**Figure 3:**
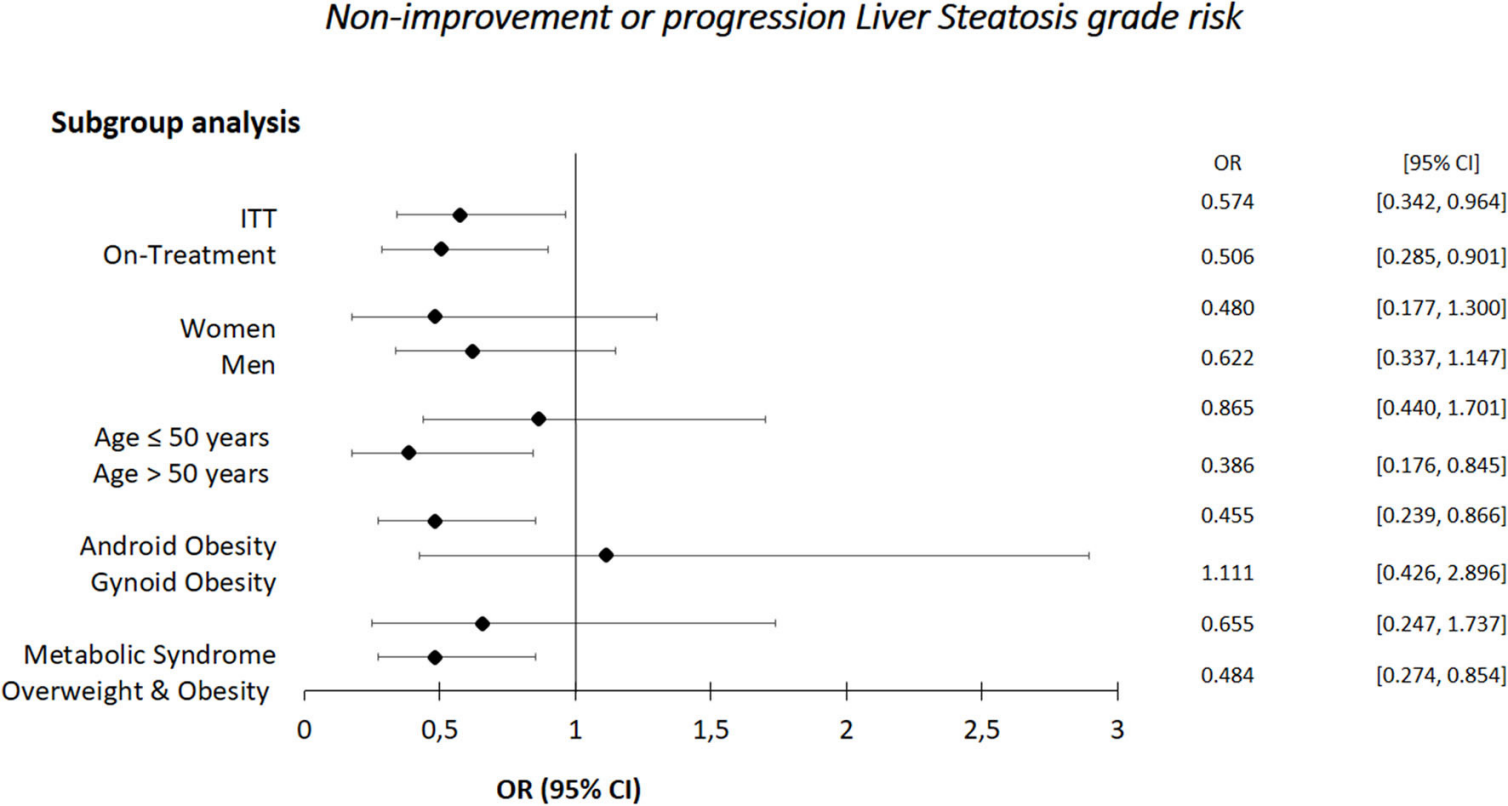
Non-improvement or progression liver steatosis risk.

Figure 3 shows non-improvement or liver steatosis progression risk according to the subgroups in individuals taking BC. The nutraceutical reduced the risk in the following subgroups: over 50 years, with android obesity, with overweight/obesity (Figure 3).

### Adverse Events

The findings are reported in the Supplemental Figure 4. The participants reported a total of 24 AEs, all of grade 1 (mild).

## Discussion

Our clinical trial examined the effects of a new nutraceutical containing a combination of polyphenols from Bergamot citrus and terpenes and other flavonoids from wild cardoon (abbreviated, BC), in reducing the liver fat content in non-diabetic individuals with NAFLD.

BC significantly reduced the CAP score by 7% (-9% in OT analysis; −26.9 ± 43 and −48 ± 39 dB/m in the placebo and BC, respectively), after 12 weeks (Tables 3, 4). The main finding was that BC reduced the CAP score by 15%, in the participants over 50 years (Table 4).

The changes observed in the CAP score were in the range of those obtained in one double-blind, randomized, placebo-controlled study with hesperidin supplementation (31). In that study, hesperidin supplementation, compared with placebo, was associated with a significant reduction in CAP score (~-31 vs ~-51 in the placebo and Hesperidin group, respectively) (31). However, in that study participants were recruited only with NAFLD grades 2 and 3, while in our study we enrolled all individuals who had some degree of steatosis (31). Furthermore, to date, we have no single ingredients working efficiently in NAFLD. Several studies performed in animal models of liver steatosis confirm our results (32, 33). It has been demonstrated that a mixture of natural citrus polyphenols from Bergamot prevents pathogenic fat accumulation in the liver of rats (11, 34). However, no further studies exist on the effects of nutraceuticals on NAFLD patients.

The findings from studies in several cellular models have indicated the ability of dietary polyphenols to counteract ROS production and its associated oxidative damage by controlling mitochondrial membrane potential and oxidative phosphorylation (33, 34). Dietary polyphenols can also act indirectly by up-regulating endogenous antioxidant defenses (35, 36). Furthermore, these molecules act as potent inducer of lipophagy in animal models of steatosis (11).

Citrus fruits are notably rich in flavonoid compounds. However, among different citrus fruits, Bergamot contains the highest concentrations of total flavone glycosides, including both flavone *O*- and *C*-glycosides (37).

A study has demonstrated that in most citrus juices, vicenin-2 is, by far, the most abundant flavone derivative with the highest level in Bergamot cultivars, followed by other fruits (38).

Although pears (*Pyrus* spp) are dietary sources of bioactive components such as polyphenols and triterpenic acids (39), the chemical composition of these fruits significantly differ from Bergamot and Wild Cardoon. Indeed, chlorogenic acid, quinic acid and arbutin are the primary polyphenols and ursolic acid is the predominant triterpenoid in thinned pears, whereas chlorogenic acid and most of the flavan-3-ols, are the main antioxidants in young pears (39).

Cynaropicrin from other source, such as *Saussurea amara*, a Mongolian medicinal plant, provokes a dose-dependent increase in bile flow in the isolated rat liver perfusion system, thus confirm our findings (15).

Using a patented extraction technology through collaborative works of various research institutions, BC contains the highest concentration available of these potent compounds. BC is composed of several, well-known, biologically active phenols, such as naringine, neohesperidine, neoeriocitrine, brutieridine, melitidine, cynaropicrin, cholotogenic acid. The largest representative sesquiterpene lactones in wild cardoon is cynaropicrin which possesses choleretic, anti-inflammatory and anti-hyperlipidemic proprieties (15, 16). Cynaropicrin has potent suppressive effects on TNF-α (17). In addition, wild cardoon recovers other biologically active compounds, such as caffeoylquinic acids, luteolin, and apigenin derivatives, all of which have potential important effects on human health (17, 18). In this study, a specification sheet of the product including the mean concentration of the most relevant active ingredients as well as the HPLC traces for both bergamot and CyC extract was provided (see Supplemental Material). Since currently we have no single molecules working efficiently in NAFLD, the synergic effect of all these polyphenols would represent a novel approach in reducing the liver fat content.

Of interest, in the present study the nutraceutical BC, compared with placebo, was associated with a significant body weight reduction (Table 3 and Supplemental Figure 3) (11). In individuals with NAFLD, interventions aimed at weight loss were associated with improvements in blood biomarkers of liver disease, as well as instrumental and histological markers, such as liver stiffness and steatosis (9). CAP score reduction seems to be explained by weight loss in our population, but not among subjects aged >50 years (*post-hoc* analyses, Table 4). Therefore, BC improved CAP score in adult population.

Our subgroup analyses yield more than one significant interaction. These significant interactions might, however, be associated with each other, and thus explained by a common factor, Thus, CAP score reduction in participants with android obesity as well as overweight-obesity could also be due to weight loss. Although such *post-hoc* analyses might not carry the same weight of evidence as the primary prespecified analysis, they offer the opportunity to explore hypotheses that might not be immediately envisaged at the time of study design and reflect the dynamic nature of clinical research (40). The potential value of *post-hoc* analyses in hypothesis generation cannot be entirely discounted, thus, the outcomes might inform the design of future studies.

Some clarification is needed regarding the lack of an expected significant difference in LDL-cholesterol reduction between groups (41). First, the main purpose of this study was not blood lipid reduction. In addition, only half of the population had hyperlipidaemia and about 15% had taken lipid-lowering agents. The use of these agents did not influence the CAP score differences (data not shown). However, in participants taking BC, we found that LDL-cholesterol dropped from 116 ± 32 to 107 ± 30 mg/dl (Table 2).

Our population had only mild hypercholesterolemia (Tables 1, 2), which limited the efficacy of BC. Moreover, it is well-known that hypercholesterolemia and inflammation are linked in a vicious cycle in which the excess of cholesterol induces an inflammatory response that, in turn, accelerates cholesterol deposition and amplifies inflammation (42).

Cytokines play a key role in inflammatory diseases and a link with hypercholesterolemia and atherosclerosis has emerged mainly for the IL-6, IL-1, and TNFα pathways (42).

All these mechanisms can explain, in our study, both the lack of the lipid-lowering and antiinflammatory effects. In fact, in this study the change in serum concentration of the white blood cells, IL-1β, IL-6, and TNF-α did not differ between groups after 12 weeks of treatment (Table 3). Based on these results, how should we interpret the effects on CAP? The potential effects of BC on the liver would not be limited to antiinflammatory and antioxidant actions. It has been dimonstrated that BPF would prevent NAFLD via stimulation of lipophagy (11). Mitochondria-associated membranes or mitochondria-endoplasmic reticulum contact sites have been implicated in the formation of autophagosomes (43). Polyphenols exert a modulatory action on several mitochondrial processes, not necessarily inflammatory-related, such as biogenesis, membrane potential maintenance, electron transport chain, ATP synthesis and cell death triggering (36). Future studies aimed at discriminating the extent to which a cytoprotective effect of BC on the liver is a consequence of its direct modulatory action on the formerly-referred mitochondrial processes or whether it also critically arises from antiflammatory/antioxidant actions, are warranted.

We found a significant CAP score reduction especially in women (Table 4). This finding is linked to a different redox state between genders (44–47). These findings should be taken into account in future studies.

Despite from a statistical point of view not significant, placebo group had a higher AST concentration at baseline, than BC group (*p* = 0.06). However, these values refer to the whole enrolled population. We performed the subsequent ITT as well as OT analyses and this initial basal difference disappeared (Table 2). Furthermore, it is unlikely, from a clinical point of view, that a similar slightly difference solely in AST translates in a significant CAP change.

BC is a safe product. The most frequent AE in BC was diarrhea but was reported only in three participants. We found 12 AEs for each groups, all of grade 1 (mild). Previously, Gliozzi et al. (48) prescribed 1,300 mg/day of BPF to 107 patients with NAFLD and metabolic syndrome for 120 days. Furthermore, in another study 32 subjects with mixed hyperlipidemia received 1,500 mg of bergamot combined with hypocaloric diet (41). None of these studies, testing a high concentration of BPF, reported any type of adverse events. In a study carried out in individuals infected by hepatitis C virus, *CyC* extract normalized ALT and AST, as well as the level of bilirubin (49). Furthermore, only a moderately interaction with human drug-metabolizing enzymes, such as CYP1A2, CYP2D6, CYP2E1, and CYP3A4, was observed (50). In our tests of potential toxicity, no incidence of significant treatment-related clinical abnormalities was found with BC throughout the studies (see Supplemental Material).

In this study, the pattern of liver enzymes change may provide insight into the differential effect of diet and BC on the liver. A minimal weight loss is always associated with ALT reduction. For every 5% weight loss, a four greater likelihood of ALT normalization was observed (51). This concept can explain the ALT reduction in both treatments. ALT reduction could suggest an enhanced hepatic lipid mobilization. In the liver, ALT is localized solely in the cellular cytoplasm, whereas AST is both cytosolic and mitochondrial (80% of total activity). In our study, AST reduced solely in BC group. Due to the peculiar intralobular distribution of AST (52), our finding may suggest a deep, intralobular effect of BC.

However, in the present study BC, compared with placebo, was associated with a significant body weight reduction but CAP score reduction was explained by weight loss only in subjects aged <50 years (Table 4). Consequently, BC improved CAP score independent of weight loss solely in adult individuals. Cell culture, animal, and some human studies suggest that consumption of polyphenols (from foods or supplements) changes energy metabolism and may facilitate weight loss or prevent weight gain (53). These potential effects may occur through a variety of mechanisms: stimulation of catabolic pathways in adipose tissue and liver, reduction of obesity-related inflammation, increase in the uptake of glucose by skeletal muscles and others (53).

This study has some limitations. Of course, findings after a 12-week intervention cannot accurately reflect the long-term effects of BC in NAFLD patients. We found a low AEs rate (50). Although the results of this study confirmed our assumption, further studies with higher dosages and longer intervention periods are needed to confirm our findings. Unfortunately we did not assess the oxidative stress markers. Another limitation is that CAP score might not be proper to evaluated steatosis stages in detail. Liver biopsy is still considered the gold standard for the diagnosis of fatty liver disease. However, because liver biopsy is an invasive procedure, in the context of the follow-up of individuals with NAFLD, it is not the most appropriate method (54).

Thus, the first-line examination is abdominal ultrasound. However, ultrasound has low sensitivity for the detection of steatosis when it affects <20% of the liver or in individuals with severe obesity. Magnetic resonance spectroscopy is costly, therefore being used only for research purposes. TE by CAP measurement has been shown to detect steatosis with good sensitivity and specificity (55). Other prospective studies are mandatory before definitively recommending this technique for the prediction of steatosis grades. However, the hypothetical bias in our study would pertain to both treatments.

Another limitation of this study is related to the interpretation of the results from *post-hoc* analyses which should be conducted with caution as they serve only in hypothesis generation.

However, this study has important strengths. Most of the currently available data on nutraceuticals pertains to *in vitro* and animal studies, whose observations and conclusions do not always extrapolate directly to humans. Our study is a randomized controlled trial, thus, as “gold standard” in evidence-based medicine, may really detect clinically relevant conclusions on the effects of this new nutraceutical. Moreover, this type of study decreases patient and observer bias. Furthermore, knowledge translation into clinical practice may rapidly occurs.

An important strength of this study is related to the stability of the active ingredients in BC. In this study we provided the batch number, which suggest that, the manufacturing activities are in accordance with the international procedures (56, 57). The batch used in this experiment has an expiration date (10–2020) and within this period the stability of the active ingredients is guaranteed. Furthermore, BC phytocomplex is analyzed for its integrity and stability every 2 months and, the flavonoid profile is intact overtime.

## Conclusion

The results of this study demonstrate that BC significantly reduces the CAP score by 15% but, from a subgroup analysis, we can confirm this finding only in the participants over 50 years. In this population with mild hypercholesterolemia, we did not observe any lipid-lowering and anti-inflammatory effect. Treatment with BC was well-tolerated and was not associated with an increased risk for adverse events. BC would constitute a promising complement to non-pharmacological measures that are commonly used to counteract the onset and progression of NAFLD, at least in individuals over 50 years. Future studies confirming our results and addressing whether long-term BC treatment can reduce the severity of NAFLD would be important for the future.

## Data Availability Statement

The datasets generated for this study are available on request to the corresponding author.

## Ethics Statement

The studies involving human participants were reviewed and approved by Mater Domini Azienda University Hospital. The patients/participants provided their written informed consent to participate in this study.

## Author Contributions

YF and EM: responsibility for the integrity of the work and methodology. TM and AP: conceptualization and original draft preparation. SP and EM: data curation. TM: writing. AP, VMu, EB, SN, and VMo: review & editing. DF, IA, and EA: laboratory Investigation. MG and VMo: funding acquisition. All authors approved the final version and contributed to the manuscript preparation and interpretation of data.

## Conflict of Interest

EB was employed by the company Plantexresearch srl, Milano. The remaining authors declare that the research was conducted in the absence of any commercial or financial relationships that could be construed as a potential conflict of interest.

## Abbreviations

NAFLD: Non-alcoholic fatty liver disease
BPF: polyphenolic fraction
BF: bergamot and wild cardoon
TE: transient elastography
BMI: body mass index
WC: waist circumference
HC: hip circumference
FM: fat mass
SBP: systolic blood pressure
DBP: diastolic blood pressure
CAP: controlled attenuation parameter
IQR: interquartile range
HOMA-IR: homeostatic model assessment of insulin resistance
TC: total cholesterol
TG: triglycerides
HDL-C: high density lipoprotein cholesterol
LDL-C: low density lipoprotein cholesterol
AST: aspartate aminotransferase
ALT: alanine aminotransferase
γGT: gamma glutamyltransferase.

## References

[1] Ekstedt M Franzén LE Mathiesen UL Thorelius L Holmqvist M Bodemar G. Long-term follow-up of patients with NAFLD and elevated liver enzymes. Hepatology. (2006) 44:865–73. 10.1002/hep.21327 17006923

[2] Dyson J Jaques B Chattopadyhay D Lochan R Graham J Das D. Hepatocellular cancer: the impact of obesity, type 2 diabetes and a multidisciplinary team. J Hepatol. (2014) 60:110–7. 10.1016/j.jhep.2013.08.011 23978719

[3] Younossi ZM Koenig AB Abdelatif D Fazel Y Henry L Wymer M Global epidemiology of non-alcoholic fatty liver disease-Meta-ana¬lytic assessment of prevalence, incidence and outcomes. Hepatology. (2016) 64:73–84. 10.1002/hep.28431 26707365

[4] Manco M Marcellini M Giannone G Nobili V. Correlation of serum TNF-alpha levels and histologic liver injury scores in pediatric nonalcoholic fatty liver disease. Am J Clin Pathol. (2007) 127:954–60. 10.1309/6VJ4DWGYDU0XYJ8Q 17509993

[5] Sumida Y Niki E Naito Y Yoshikawa T. Involvement of free radicals and oxidative stress in NAFLD/NASH. Free Radic Res. (2013) 47:869–80. 10.3109/10715762.2013.837577 24004441

[6] Czaja MJ. Liver injury in the setting of steatosis: crosstalk between adipokine and cytokine. Hepatology. (2004) 40:19–22. 10.1002/hep.20328 15239081

[7] Mehta K Van Thiel DH Shah N Mobarhan S. Nonalcoholic fatty liver disease: pathogenesis and the role of antioxidants. Nutr Rev. (2002) 60:289–93. 10.1301/002966402320387224 12296456

[8] Liu K Czaja MJ. Regulation of lipid stores and metabolism by lipophagy. Cell Death Differ. (2013) 20:3–11. 10.1038/cdd.2012.63 22595754 PMC3524634

[9] Koutoukidis DA Astbury NM Tudor KE Morris E Henry JA Noreik M. Association of weight loss interventions with changes in biomarkers of nonalcoholic fatty liver disease: a systematic review and meta-analysis. JAMA Intern Med. (2019) 179:1303–4. 10.1001/jamainternmed.2019.2248 31260026 PMC6604126

[10] Ferramosca A Di Giacomo M Zara V. Antioxidant dietary approach in treatment of fatty liver: new insights and updates. World J Gastroenterol. (2017) 23:4146–57. 10.3748/wjg.v23.i23.4146 28694655 PMC5483489

[11] Parafati M Lascala A Morittu VM Trimboli F Rizzuto A Brunelli E. Bergamot polyphenol fraction prevents nonalcoholic fatty liver disease via stimulation of lipophagy in cafeteria diet-induced rat model of metabolic syndrome. J Nutr Biochem. (2015) 26:938–48. 10.1016/j.jnutbio.2015.03.008 26025327

[12] Siddiqi A Hasan SK Nafees S Rashid S Saidullah B Sultana S. Chemopreventive efficacy of hesperidin against chemically induced nephrotoxicity and renal carcinogenesis via amelioration of oxidative stress and modulation of multiple molecular pathways. Exp Mol Pathol. (2015) 99:641–53. 10.1016/j.yexmp.2015.11.012 26551080

[13] El-Sisi AEE Sokar SS Shebl AM Mohamed DZ. Antifibrotic effect of diethylcarbamazine combined with hesperidin against ethanol induced liver fibrosis in rats. Biomed Pharmacother. (2017) 89:1196–206. 10.1016/j.biopha.2017.03.013 28320086

[14] Mahmoud AM Mohammed HM Khadrawy SM Galaly SR. Hesperidin protects against chemically induced hepatocarcinogenesis via modulation of Nrf2/ARE/HO-1, PPARγ and TGF-β1/Smad3 signaling, and amelioration of oxidative stress and inflammation. Chem Biol Interact. (2017) 277:146–58. 10.1016/j.cbi.2017.09.015 28935427

[15] Glasl S Tsendayush D Batchimeg U Holec N Wurm E Kletter C. Choleretic effects of the mongolian medicinal plant saussurea amara in the isolated perfused rat liver. Planta Med. (2007) 73:59–66. 10.1055/s-2006-957063 17177133

[16] Elsebai MF Mocan A Atanasov AG. Cynaropicrin: A Comprehensive Research Review and Therapeutic Potential As an Anti-Hepatitis C Virus Agent. Front Pharmacol. (2016) 7:472. 10.3389/fphar.2016.00472 28008316 PMC5143615

[17] Cho JY Park J Yoo ES Baik KU Jung JH Lee J. Inhibitory effect of sesquiterpene lactones from saussurea lappa on tumor necrosis factor-alpha production in murine macrophage-like cells. Planta Med. (1998) 64:594–7. 10.1055/s-2006-957528 9810262

[18] Kukic J Popovic V Petrovic S Mucaji P Ciric A Stojkovic D Antioxidant and antimicrobial activity of cynara cardunculus extracts. Food Chem. (2008) 107:861–8. 10.1016/j.foodchem.2007.09.005

[19] Parker HM Cohn JS O'Connor HT Garg ML Caterson ID George J. Effect of fish oil supplementation on hepatic and visceral fat in overweight men: a randomized controlled trial. Nutrients. (2019) 11:E475. 10.3390/nu11020475 30813440 PMC6413081

[20] Mazza E Fava A Ferro Y Rotundo S Romeo S Bosco D. Effect of the replacement of dietary vegetable oils with a low dose of extravirgin olive oil in the mediterranean diet on cognitive functions in the elderly. J Transl Med. (2018) 16:10. 10.1186/s12967-018-1386-x 29351799 PMC5775590

[21] Ferro Y Carè I Mazza E Provenzano F Colica C Torti C. Protein and vitamin B6 intake are associated with liver steatosis assessed by transient elastography, especially in obese individuals. Clin Mol Hepatol. (2017) 23:249–59. 10.3350/cmh.2017.0019 28750503 PMC5628006

[22] de Lédinghen V Vergniol J Foucher J Merrouche W le Bail B. Non-invasive diagnosis of liver steatosis using controlled attenuation parameter (CAP) and transient elastography. Liver Int. (2012) 32:911–8. 10.1111/j.1478-3231.2012.02820.x 22672642

[23] Myers RP Pollett A Kirsch R Pomier-Layrargues G Beaton M Levstik M. Controlled Attenuation Parameter (CAP): a noninvasive method for the detection of hepatic steatosis based on transient elastography. Liver Int. (2012) 32:902–10. 10.1111/j.1478-3231.2012.02781.x 22435761

[24] Psaty BM Furberg CD Kuller LH Bild DE Rautaharju PM Polak JF. Traditional risk factors and subclinical disease measures as predictors of first myocardial infarction in older adults: the cardiovascular health study. Arch Intern Med. (1999) 59:1339–47. 10.1001/archinte.159.12.1339 10386510

[25] Centers for Disease Control and Prevention (CDC). Use of cessation methods among smokers aged 16–24 years—United States, 2003. MMWR Morb Mortal Wkly Rep. (2006) 55:1351–4. 17183227

[26] Expert Panel on Detection, Evaluation and Treatment of High Blood Cholesterol in Adults Executive summary of the third report of the National Cholesterol Education Program (NCEP) expert panel on detection, evaluation, and treatment of high blood cholesterol in adults (adult treatment panel III). JAMA. (2001) 285:2486–97. 10.1001/jama.285.19.2486 11368702

[27] Nishida C Ko GT Kumanyika S. Body fat distribution and noncommunicable diseases in populations: overview of the 2008 WHO expert consultation on waist circumference and waist-hip ratio. Eur J Clin Nutr. (2010) 64:2–5. 10.1038/ejcn.2009.139 19935820

[28] Montalcini T Gorgone G Federico D Ceravolo R Emanuele V Sesti G. Association of LDL cholesterol with carotid atherosclerosis in menopausal women affected by the metabolic syndrome. Nutr Metab Cardiovasc Dis. (2005) 15:368–72. 10.1016/j.numecd.2004.10.003 16216723

[29] Matthews DR Hosker JP Rudenski AS Naylor BA Treacher DF Turner RC. Homeostasis model assessment: insulin resistance and beta-cell function from fasting plasma glucose and insulin concentrations in man. Diabetologia. (1985) 28:412–9. 10.1007/BF00280883 3899825

[30] Ferraioli G Tinelli C Lissandrin R Zicchetti M Faliva M Perna S Correlation of the controlled attenuation parameter with indices of liver steatosis in overweight or obese individuals: a pilot study. Eur J Gastroenterol Hepatol. (2015) 27:305–12. 10.1097/MEG.0000000000000287 25629575

[31] Cheraghpour M Imani H Ommi S Alavian SM Karimi-Shahrbabak E Hedayati M. Hesperidin improves hepatic steatosis, hepatic enzymes, and metabolic and inflammatory parameters in patients with nonalcoholic fatty liver disease: a randomized, placebo-controlled, double-blind clinical trial. Phytother Res. (2019) 33:2118–25. 10.1002/ptr.6406 31264313

[32] Zhu W Chen S Chen R Peng Z Wan J Wu B. Taurine and tea polyphenols combination ameliorate nonalcoholic steatohepatitis in rats. BMC Complement Altern Med. (2017) 17:455. 10.1186/s12906-017-1961-3 28886741 PMC5591522

[33] Parafati M Lascala A La Russa D Mignogna C Trimboli F Morittu VM. Bergamot polyphenols boost therapeutic effects of the diet on non-alcoholic steatohepatitis (NASH) induced by “junk food”: evidence for anti-inflammatory activity. Nutrients. (2018) 1:10. 10.3390/nu10111604 30388763 PMC6267059

[34] Serrano JC Cassanye A Martín-Gari M Granado-Serrano AB Portero-Otín M. Effect of dietary bioactive compounds on mitochondrial and metabolic flexibility. Diseases. (2016) 4:E14. 10.3390/diseases4010014 28933394 PMC5456301

[35] Olayinka ET Ore A Adeyemo OA Ola OS Olotu OO Echebiri RC. Quercetin, a flavonoid antioxidant, ameliorated procarbazine-induced oxidative damage to murine tissues. Antioxidants. (2015) 4:304–21. 10.3390/antiox4020304 26783707 PMC4665474

[36] Sandoval-Acuña C Ferreira J Speisky H. Polyphenols and mitochondria: anupdate on their increasingly emerging ROS-scavenging independent actions. Arch Biochem Biophys. (2014) 559:75–90. 10.1016/j.abb.2014.05.017 24875147

[37] Hostetler GL Ralston RA Schwartz SJ. Flavones: food sources, bioavailability, metabolism, and bioactivity. Adv Nutr. (2017) 8:423–35. 10.3945/an.116.012948 28507008 PMC5421117

[38] Barreca D Mandalari G Calderaro A Smeriglio A Trombetta D Felice MR. Citrus Flavones: an update on sources, biological functions, and health promoting properties. Plants. (2020) 9:288. 10.3390/plants9030288 32110931 PMC7154817

[39] Sun L Tao S Zhang S. Characterization and quantification of polyphenols and triterpenoids in thinned young fruits of ten pear varieties by UPLC-Q TRAP-MS/MS. Molecules. (2019) 24:159. 10.3390/molecules24010159 30609827 PMC6337724

[40] Curran-Everett D Milgrom H. *Post-hoc* data analysis: benefits and limitations. Curr Opin Allergy Clin Immunol. (2013) 13:223–4. 10.1097/ACI.0b013e3283609831 23571411

[41] Mollace V Sacco I Janda E Malara C Ventrice D Colica C. Hypolipemic and hypoglycaemic activity of bergamot polyphenols: from animal models to human studies. Fitoterapia. (2011) 82:309–16. 10.1016/j.fitote.2010.10.014 21056640

[42] Catapano AL Pirillo A Norata GD. Vascular inflammation and low-density lipoproteins: is cholesterol the link? A lesson from the clinical trials. Br J Pharmacol. (2017) 174:3973–85. 10.1111/bph.13805 28369752 PMC5659993

[43] Hamasaki M Furuta N Matsuda A Nezu A Yamamoto A Fujita N. Autophagosomes form at ER-mitochondria contact sites. Nature. (2013) 495:389–93. 10.1038/nature11910 23455425

[44] Borrás C Sastre J García-Sala D Lloret A Pallardó FV Viña J. Mitochondria from females exhibit higher antioxidant gene expression and lower oxidative damage than males. Free Radic Biol Med. (2003) 34:546–52. 10.1016/S0891-5849(02)01356-4 12614843

[45] Ide T Tsutsui H Ohashi N Hayashidani S Suematsu N Tsuchihashi M. Greater oxidative stress in healthy young men compared with premenopausal women. Arterioscler Thromb Vasc Biol. (2002) 22:438–42. 10.1161/hq0302.104515 11884287

[46] Block G Dietrich M Norkus EP Morrow JD Hudes M Caan B. Factors associated with oxidative stress in human populations. Am J Clin Nutr. (2002) 156:274–85. 10.1093/aje/kwf029 12142263

[47] Keaney JF Jr Larson MG Vasan RS Wilson PW Lipinska I Corey D. Obesity and systemic oxidative stress: clinical correlates of oxidative stress in the framingham study. Arterioscler Thromb Vasc Biol. (2003) 23:434–9. 10.1161/01.ATV.0000058402.34138.11 12615693

[48] Gliozzi M Carresi C Musolino V Palma E Muscoli C Vitale C The effect of bergamot-derived polyphenolic fraction on LDL small dense particles and non alcoholic fatty liver disease in patients with metabolic syndrome. Adv Biol Chem. (2014) 4:129 10.4236/abc.2014.42017

[49] Elsebai MF Abass K Hakkola J Atawia AR Farag MA. The wild Egyptian artichoke as a promising functional food for the treatment of hepatitis C virus as revealed via UPLC-MS and clinical trials. Food Funct. (2016) 7:3006–16. 10.1039/C6FO00656F 27296047

[50] Di Lorenzo C Ceschi A Kupferschmidt H Lüde S De Souza Nascimento E Dos Santos A. Adverse effects of plant food supplements and botanical preparations: a systematic review with critical evaluation of causality. Br J Clin Pharmacol. (2015) 79:578–92. 10.1111/bcp.12519 25251944 PMC4386943

[51] Suzuki A Lindor K St Saver J Lymp J Mendes F Muto A. Effect of changes on body weight and lifestyle in nonalcoholic fatty liver disease. J Hepatol. (2005) 43:1060–6. 10.1016/j.jhep.2005.06.008 16140415

[52] Seeto RK Fenn B Rockey DC. Ischemic hepatitis: clinical presentation and pathogenesis. Am J Med. (2000) 109:109–13. 10.1016/S0002-9343(00)00461-7 10967151

[53] Meydani M Hasan ST. Dietary polyphenols and obesity. Nutrients. (2010) 2:737–51. 10.3390/nu2070737 22254051 PMC3257683

[54] Cosgrove D Piscaglia F Bamber J Bojunga J Correas JM Gilja OH. EFSUMB guidelines and recommendations on the clinical use of ultrasound elastography. Part 2: clinical applications. Ultraschall Med. (2013) 34:238–53. 10.1055/s-0033-1335375 23605169

[55] Shi KQ Tang JZ Zhu XL Ying L Li DW Gao J. Controlled attenuation parameter for the detection of steatosis severity in chronic liver disease: a meta-analysis of diagnostic accuracy. J Gastroenterol Hepatol. (2014) 29:1149–58. 10.1111/jgh.12519 24476011

[56] WHO Technical Reports Series (1996), n. 863. Available online at: https://extranet.who.int/prequal/content/who-technical-report-series

[57] WHO Technical Reports Series (2016), n.999. Available online at: https://extranet.who.int/prequal/content/who-technical-report-series

