# Supplementary material for: Randomized Clinical Trial: Bergamot Citrus and Wild Cardoon Reduce Liver Steatosis and Body Weight in Non-diabetic Individuals Aged Over 50 Years
Source: Front Endocrinol (Lausanne). 2020 Aug 11;11:494. doi: 10.3389/fendo.2020.00494 (PMC7431622; doi:10.3389/fendo.2020.00494)
Supplement: Supplementary file 1 [file Data_Sheet_1.zip › Sup. material. 1-BC 28 days toxicology.pdf]

FINAL REPORT

Repeated dose 28-days Oral (Gavage) Toxicity of a composition with Bergamot Poliphenolic Fraction (BPF) and Cynara Carduncolus extract (Bergacyn) in Wistar Rats

Test Article:
Bergacyn composition

Sponsor:
Herbal & Antioxidant Derivatives srl (HEAD)
C.da Chiusi – 89032 BIANCO (RC)

Testing Facility:
Nutramed Scarl
Complesso Ninì Barbieri,
Roccelletta di Borgia (CZ)

Study
N.152- 0041

Author:
Sara Paone, PhD

Study Completion Date: December 20 2016

NUTRAMED S.C.A.R.L.
Complesso Ninì Barbieri
88021 Roccelletta di Borgia (Cz) – Italy
Tel. 09613695732
Mail: massimo.fini@sanraffaele.it

TABLE OF CONTENTS

TABLE OFCONTENTS 2
COMPLIANCE STATEMENT 3
QUALITYASSURANCESTATEMENT 4
SIGNATURE PAGE 5
STUDYPERSONNEL 6
STUDYTIMETABLE 7
SUMMARY 8
INTRODUCTION 10
MATERIALS ANDMETHODS 11
Test andcontrolarticles 11
Test animals&Husbandry 11
Experimentaldesign 13
Administration of thetest article 14
Pathology 16
Terminalstudies 18
Statisticalanalysis 18
RESULT 19
Mortality 19
ClinicalSigns 19
BodyWeights 19
FoodConsumption 19
Clinical Pathology 20
Haematology 20
ClinicalChemistry 20
OrganWeights 20
GrossPathology 20
CONLUSION 21
SUMMARY DATA(TABLES4-13) 22
REFERENCES 32

COMPLIANCE STATEMENT

Repeated dose 28-days Oral (Gavage) Toxicity of a composition with Bergamot Poliphenolic Fraction (BPF) and Cynara Cardunculus extract (Bergacyn) in Wistar Rats

This study was conducted in compliance with the EU Directive 2004/9/EC and Directive 2004/9/EC for Good Laboratory Practice Guidelines (GLP) and with OECD Guidelines for Repeated Dose 30-day Oral Toxicity Study in Rodents (n. 407 Adopted October 3rd 2008)

Study Director: Date

Sara Paone, PhD,DABT Date 05/01/2016

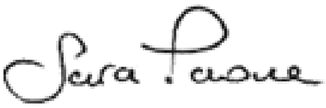

QUALITY ASSURANCE STATEMENT

Repeated dose 28-days Oral (Gavage) Toxicity of a composition with Bergamot Polyphenolic Fraction extract (BPF) and Cynara Cardunculus extract (Bergacyn) in Wistar Rats

This study, n. 152-00041 entitled “Repeated dose 28-day Oral (Gavage) Toxicity of Bergamot Polyphenolic Fraction (BPF) and CynaraCardunculus extract (Bergacyn) in Wistar Rats with a 2-Week Recovery” was inspected/audited by Quality Assurance in accordance with San Raffaele IRCCS Guidelines and OECD Good Laboratory Practice Regulations. All findings were reported to the Study Director and Testing Facility Management.

Date of Inspection	Phase of the Study	Date of Reporting
July 30, 2016	Randomization and grouping of animals	July 30, 2016
August 3,2016	Administration of Test Article; Clinical Examination & Animal Weighing	August 3, 2016
August 03, 2016	Administration of Test Article;	August 03, 2016
September 1,2016	Clinical Examination & Food Weight Measurement Clinical Examination,Functional	September 1, 2016
September 15,2015	Observations Terminal Necropsy	September 15, 2016
September 30,2016	Terminal Necropsy:Reversal Groups	October 2,2016
December 20,2016	Report Audit	December 20,2016

Action has been taken in response to all items listed by Quality Assurance. It is concluded that the final report accurately reflects NutraMed SCARL- San Raffaele Standard Operating Procedures and the raw data for this study.

Michelangelo Iannone
Sr. Manager QualityAssurance

Date

05.01.2017

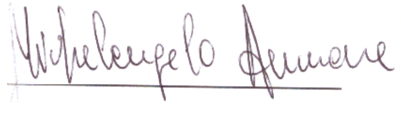

SIGNATURE PAGE

Repeated dose 28-days Oral (Gavage) Toxicity of Bergamot Polyphenolic Fraction and CynaraCardunculus extract (Bergacyn) in Wistar Rats.

Author

05/01/2016

Sara Paone, PhD, DABT
StudyDirector

Date

STUDY PERSONNEL

StudyDirector:	Sara Paone, PhD
ToxicologyAssociate:	Luigi Tucci, PhD
ReportAssociate:	Luca Gareri,PhD
HeadTechnicians:	Giovanni Politi,BS
Supervisor,Necropsy:	Francesca Oppedisano, BS
Manager,Formulations:	RosalbaMacri', BS
Pathologist:	CaterinaCriaco,PhD
Toxicology:	Federica Scarano, PhD, DABT Director,Laboratory
AnimalMedicine:	Nicola Costa,PhD

Sponsor: Herbal & Antioxidant Derivatives srl (HEAD)
C.da Chiusi – 89032 BIANCO (RC)

Sponsor Representative/Study Monitor: Giuseppe Lombardo

STUDY TIMETABLE

Study Initiation Date:	July 30,2016
Experimental Start Date:	August 03,2016
ReceiptofAnimals:	July 03,2016
RandomizationofAnimals:	July 30, 2016
First Day of Dosing:	August 03,2016
Last Day of Dosing:	August 30, 2016
Necropsy: Terminal Sacrifice	September 15,2016
Experimental Completion Date:	October 30,2016
StudyCompletionDate:	December 202016

SUMMARY

Repeated dose 28-days Oral (Gavage) Toxicity of Bergamot Polyphenolic Fraction (BPF) and Cynara Cardunculus extract (Bergacyn) in Wistar Rats

Male and female Wistar rats (10 male and 10 female for each group) received orally BPF and Cynara Cardunculus extract (Bergacyn) (Lot. No. 1701/16) by gastric gavage daily at the doses of 50 mg/kg, 100 mg/kg or 150 mg/kg body weight for 28 consecutive days and were sacrificed on day 29 to evaluate its toxicity. Bergacyn was diluted in vehicle (in demineralised water) at the concentration 10, 40, 200 mg/ml respectively to allow a constant dose of the vehicle (max 2 ml/100 g body weight) to be administered together with the test article. Concurrent control groups receiving vehicle at 2 ml/100 g body weight were also maintained. Additionally, satellite groups of six rats receiving vehicle at 2 ml/100 g body weight and six rats receiving Bergacyn at the highest dose of 150 mg/kg body weight were further observed for a period of 14 days following 28 days exposure, for the assessment of reversibility, persistence or delayed occurrence of toxicity.

The rats were examined daily for signs of toxicity, morbidity and mortality. They were subjected to detailed clinical examination before initiation of the study and weekly thereafter during the exposure period, reversal period, and at termination. In the first, and fourth week of exposure they were additionally examined for assessment of sensory reactivity, assessment of grip strength and motor activity. Body weight and food consumption were recorded weekly. Laboratory investigations were performed on blood samples at termination of the study. All animals sacrificed terminally were subjected to a detailed necropsy and weights of certain organs were recorded.

Histopathological evaluation showed that no organ alterations were encountered. There was no incidence of treatment related mortality in rats exposed to Bergacyn at 50, 100 and 150 mg/kg body weight. The test article did not induce any remarkable and treatment related clinical abnormalities in rats treated at and up to the dose of 150 mg/kg. No mortality or abnormal clinical signs were observed in animals treated with vehicle. Also, the functional observations conducted throughout the time of exposure did not reveal any neurotoxic effects of the test article up to its highest level of exposure.

The hematological parameters of hemoglobin, hematocrit and total RBC counts, leucocyte counts, platelet count and clotting time of rats exposed to the test article at and up to the level of 150 mg/kg were found to be comparable to those of the vehicle control animals at termination of the treatment and also at the end of recovery period.

The test article, at and up to the level of 150 mg/kg, did not alter the plasma levels of total protein, albumin, alanine aminotransferase (ALT), alkaline phosphatase (ALP), gamma glutamyl traspeptidase (GGT), glucose, creatinine, urea, sodium and potassium in Wistar rats. The values of absolute and relative weights of liver, kidneys, adrenals, spleen, heart, brain, testes, thymus and epididymides of male and female rats treated with Bergacyn at and up to 150 mg/kg were found to be comparable with those of the control rats at the end of treatment period and also at the end of recovery period. Bergacyn did not induce any treatment related gross pathological alterations in treated rats at and up to the dose of 150 mg/kg. Based on the findings of this study the no observable adverse effect level (NOAEL) of Bergacyn in Wistar rats, following oral administration for 28 days, was found to be more than 150 mg/kg bodyweight.

INTRODUCTION

Bergamot (Citrus bergamia) is an endemic plant of the Calabrian region in Southern Italy with a unique profile of flavonoid and flavonoid glycosides present in its juice and albedo, such as neoeriocitrin, neohesperidin, naringin, rutin, neodesmin, rhoifolin and poncirin. Bergamot differs from other Citrus fruits not only because of the composition of its flavonoids, but also because of their particularly high content. Among them naringin, present also in grapefruit, has already been reported to be active in animal models of atherosclerosis, while neoeriocitrin and rutin have been shown to inhibit LDL oxidation. Importantly, bergamot juice is rich in neohesperidosides of hesperetin and naringenin, such as melitidine and brutieridine. These flavonoids, recently isolated in the Bergamot Polyphenolic Fraction (BPF) possess a 3-hydroxy-3- methylglutaryl moiety with a structural similarity to the natural substrate of HMG-CoA reductase and exhibit statin-like proprieties. On the other hand, BPF has been found to improve the liver cell functionality in subjects undergoing Non Alcohoolic Fatty Liver Disease (NAFLD) thereby contributing in the physiological packaging of lipoproteins in the liver and, finally, in counteracting dyslipidemia. These properties seem to combine efficiently with plant extracts characterized by hepatoprotective properties such as artichoke extracts.

The cultivation of artichoke comes from the mists of the past. The plant seems to be native to Ethiopia or Egypt, whence it was brought to other Mediterranean areas of civilization. The common name of the plant derives from the Arabic “alharsuf” (“ground-thorn”) which in the different countries, throughout the centuries, became “alcachofa” in Spanish, “alcachorfa” in Portuguese, “artichaut” in French. In English language, it is also known as “globe artichoke”. The scientific name, *Cynara* derives from a Roman consolidated use to manure the plant with ash *“cinere”* in Latin.

The plant is always very appreciated as a food, but recently, the therapeutic potential of Cynara Cardunculus has also been investigated in human studies.

In particular, experimental and epidemiological studies have demonstrated that Cynara Cardunculus extract (CyC) possesses choleretic, cholagogue, diuretic, laxative, anti-gout propertiesa; it also ameliorates serum lipemic profile. The pharmacological and therapeutic actions of the artichoke in liver disorders have been demonstrated by clinical experiments in which ascertained a marked therapeutic activity, particularly in some patients suffering from different forms of jaundice. Researchers observed a rapid disappearance of salts and biliary pigments from urine, restoration of normal color in faeces, simultaneous clearing of the skin and a considerable diuretic effect. The double action, choleretic and diuretic, of the artichoke, was connected to its high content in potassium and magnesium salts. The choleretic activities of artichoke are to be attributed to the active part of leaves.In addition, also the preparations made from artichoke roots

were able to quadruplicate bile secretion, in the same way as those obtained from the leaves.

The purpose of this study was to determine the toxicity of a composition of BPF and Cynara Cardunculus (Bergacyn) in male and female Wistar rats when administered once daily by oral gavage for 30 consecutive days. The rat was selected because it is the standard species for use in toxicology studies for EMA and FDA Guidelines, and because this study was conducted in accordance with the regulatory guideline; alternatives could not be considered. The oral gavage approach was selected because it is the intended route of administration to humansubjects.

METHODS AND MATERIALS

Test and Control Articles

The neat test and control articles used was BPF and Cynara Cardunculus extract (Bergacyn) provided by Herbal and Antioxidant Derivative ssrl (Bianco, Italy – Lot. No.1701/16).

Analitical purity controls have been performed via LC-MS detection at the IRC-FSH, University of Catanzaro, Italy as previously described (see ref. 1)

Reserve samples of the test article were taken at IRC-FSH prior to use on this study. The samples were archived at San Raffaele – Centro del Farmaco under the same conditions as the test and control articles.

Any remaining test and control articles were returned to the Sponsor following completion of the study.

Test Animals and Husbandry

Animals

Animal information is provided in theTable 1.

Table 1: Animal Information

Parameter	Males and Females
Species and Strain	Wistar rats
Supplier	Charles River
Number of Animals Received	125
Number of Animals Used on Study	120
Age of Animals at First Dose	8 to 9 weeks
Body Weight Range at First Dose	220,5-231,4 g

Animals were acclimated to laboratory conditions for 10 days prior to the first dose and released from quarantine by a staff veterinarian. During the acclimation period, each animal was identified by a temporary number that was recorded on each cage label.

NutraMed Animal Care and Use Committee (IACUC) approved this protocol and found it to be in accordance with provisions of the EU Policy on Humane Care and Use of Laboratory Animals, and the Italian Interagency Research Animal Committee Principles for the Utilization and Care of Research Animals.

Husbandry

Animal husbandry was provided as described in Table 2.

Table 2: Husbandry

**Feed[a]**	Certified Global Harlan Laboratory Rodent Diet
**Water[b]**	Water via an automatic watering system and water bottles
**Bedding[c]**	Certified hardwood bedding
**Caging**	Polycarbonate cages
**Racks**	Stainless steel racks
**Animals Per Cage**	One
**Temperature Range**	64 to 79°F
**Humidity Range**	30 to 70%
**Light Cycle**	12-hour light/12-hour dark, interrupted as necessary for study-related events
**Air Changes**	Minimum of 10 air changes perhour

[a] Feed was analyzed by the manufacturer for concentrations of specified heavy metals, aflatoxin, organophosphates and specific nutrients

[b]The number was routinely analyzed for contaminants and specific microbs

[c] The bedding was analyzed by the manufacturer for acceptable levels on heavy metals, afatoxins, bacteria, yeasts, and organophosphates prior to certification

Feed and water were provided *ad libitum*. No contaminants were known to be present in the diet, water, or bedding at levels that might have interfered with achieving the objectives of the study.

Environmental controls were set to maintain animal room conditions as shown in Table 2. Actual temperature and relative humidity in the animal room or zone were monitored continuously by a computerized system and manually recorded at least once daily. All environmental parameters were maintained within the protocol requirements.

Experimental Design

Group Assignment and Doses

Groups of ten male and ten female Wistar rats were administered with Bergacyn *(*Lot. No. 1701/16*)* by oral gavage daily at the doses of 50 mg/kg, 100 mg/kg or 150 mg/kg body weight for 28 days and were sacrificed on day 29 to evaluate its toxicity. Concurrent control group receiving vehicle at 2 ml/ 100 g body weight (in demineralised water) was also maintained. Additionally, satellite groups of six rats receiving vehicle at 2 ml/ 100 g body weight and the test article at 150 mg/kg levels were further observed for a period of 14 days following the 30 day exposure, for assessment of reversibility, persistence or delayed occurrence of toxicity. The Groups of animals and the related treatment regimens are displayed in the Table 3

Table 3

Groups of animals used throughout the study

	Group	Dosage (mg/kg)	No.	Male rats IDs	Female rats IDs
**G1**	Control	0	20	SCC1 -SCCS	SCC1F -SCCSF
**G1R**	Control, Reversal	0	20	SRCI1- SRC5	SRCI1F- SRC5F
**G2**	Low	50	20	SCL1 -SCL5	SCL1F -SCL5F
**G3**	Intermediate	100	20	SCM1-SCM5	SCM1F-SCM5F
**G4**	High	150	20	SCH1- SCH5	SCH1F- SCH5F
**G4R**	High, Reversal	150	20	SHR1- SHR5	SHR1F- SHR5F

	Total		120		

Dose Formulation

Bergacyn *(*Lot. No. 1701/16*)* was suspended in demineralised water and given orally at doses of 50 mg, 100 mg and 150 mg/kg for which the concentrations of 10 mg/ml, 40 mg/ml and 200 mg/ml were chosen respectively to give a constant dosage volume rate of 2 ml/ 100 g body weight.

The formulations were freshly prepared on each day of dosing.

Administration of the test article

The animals were dosed by oral gavage at approximately the same time each day where possible, using a 2.5 ml graduated disposable syringe and a stainless steel intubation needle (16 G). The dosage volume administered to each individual rat was adjusted according to its most recently recorded body weight. The control group rats received demineralised water by oral gavage at the same dosage volume of 2 ml/ 100 g body weight. Treatment in this manner was continued once a day, seven days a week, for a total period of 28 days.

Observations

The animals were monitored during the course of treatment for mortality, body weight gain, food consumption and clinical signs of toxicity by general and detailed clinical and functional examinations as listed below. Rats belonging to the reversal group were further observed for a post-treatment period of 14 days to permit evaluation of the persistence or reversibility of any toxic effects, or the occurrence of delayed toxicity.

Mortality

Throughout the study, all animals were checked early on each working day and again in the afternoon to look for dead or moribund animals to allow necropsy examination to be carried out during the working hours of that day.

Body Weight

The weight of each rat was recorded at the time of allocation of the animal to groups, on the day of commencement of treatment, weekly thereafter and at necropsy. Weights of reversal group rats were recorded weekly during post-treatment period and at necropsy.

Food Consumption

The quantity of food consumed by rats in each cage was recorded on the day of commencement of treatment and weekly thereafter. Food intake per rat (for 24 hours) was calculated using the amount of food offered to and left in each cage in each group, and the number of rats in each cage. Food consumption of reversal group rats was recorded weekly during post-treatment period.

Clinical Signs

All signs of poor health, together with any behavioural changes or reaction to treatment were recorded for individual animals.

Dated and signed records of appearance, change and disappearance of clinical signs were maintained on clinical history sheets for individual animals.

General Clinical Examinations

The rats were daily subjected to general cage side clinical examinations, at the same time each day, and at suitable intervals after dosing, considering the peak period of anticipated effects after dosing.

Detailed Clinical Examinations

The rats were subjected to detailed clinical examinations before initiation of the study and weekly thereafter during the study. These were conducted outside the home cage in a standard arena and at the same time, the observers being unaware of the treatment.

Signs noted included, but were not limited to, changes in skin, fur, eyes, and mucous membranes, occurrence of secretions and excretions and autonomic activity such as lacrimation, pilo-erection, pupil size, and unusual respiratory pattern.

Changes in gait, posture and response to handling as well as the presence of clonic or tonic movements, stereotypes or bizarre behaviour were also recorded.

Functional Observations

During the four week of exposure, following the detailed clinical examination, animals were additionally examined for assessment of sensory reactivity, assessment of grip strength and motor activity.

PATHOLOGY

Clinical Pathology

Food was removed overnight from animals to be sampled for laboratory investigations. Samples of blood were withdrawn at termination of treatment/recovery period, under carbon dioxide anesthesia, by cardiac puncture. The samples were collected in tubes containing Heparin (for clinical chemistry) and EDTA (for haematology) as an anticoagulant. Blood smears were also made on glass slides.

The estimations that were performed on blood samples have been listed below, together with an abbreviated title (used in Appendices andTables).

Haematology

Haematological evaluations of the blood samples were performed using an COBAS m 511 Haematology System (Roche). The following parameters were recorded, with their units of measurement as listed below:

- Haemoglobin (Hb) (mg/dl)
- Hematocrite (%) (HCT)
- Total red cell count(Total RBC) ($x10^6/\mu l$)
- Total white cell count (Total WBC) ($xl0^3/\mu l$)
- Mean corpuscular volume (MCV) (pg)
- Mean corpuscular hemoglobin (MCH) (fl)
- Mean corpuscular hemoglobin concentration (MCHC) (g/dl)
- Platelet count ($x10^3/\mu l$)
- Differential WBCcounts (% of Total WBC):

  Neutrophils (N), Eosinophils (E), Lymphocytes (L), Monocytes(M)
- Clotting time measurement (seconds) was performed manually using standard techniques.

Clinical Chemistry

Chemical and biochemical serum parameters were analyzed using a BT 4500 Clinical Chenistry Analyzer, Biotecnica Instruments, Italy, according to manufacturer's instructions. The following parameters were measured, with their units of measurement as listed below:

- Total Protein (g/dl)
- Albumin(g/dl)
- Alanine aminotransferase (ALT)(IU/1)
- Cholesterol (Cholest)(mg/dl)

- Urea (mg/dl)
- Potassium(mmol/1)
- Alkaline phosphatase (ALP)(IU/1)
- Gamma-glutamyltranspeptidase (GGT)(IU/1)
- Triglycerides(mg/dl)
- Glucose (mg/dl)
- Creatinine (mg/dl)
- Sodium(mmol/1)

TERMINAL STUDIES

Necropsy Examination

On completion of 28 days of treatment and/or 14 days of reversal period, all surviving rats were sacrificed by exsanguination (terminal cardiac puncture) under carbon dioxide anaesthesia. All the tissues listed in Table 12, from all animals, were preserved, in 10% neutral bufferedformalin.

Organ Weights

The following organs from all animals, killed at the scheduled sacrifices, were dissected free of fat and weighed wet as soon as possible to avoid drying: kidneys, liver, adrenals, testes, epididymides, thymus, spleen, brain, heart.

STATISTICAL ANALYSIS

Statistical analysis of Bergacyn treatment effects on all measured parameters (rat body weights, organ weights, food consumption and on haematology and clinical chemistry results) Bartlett's test was performed on each set of data to ensure that variance of the sets are homogenous. In case of homogenous sets of data ANOVA was performed to determine the treatment effects. Finally, Dunnett's test was employed to establish if any mean value of results from treatment groups (G2, G3, G4+G4R, G4R) was significantly different compared to a mean value of the control group (G1, G1+G1 R,G1R).

In case of heterogeneous data, F test was carried out to determine which pairs of groups are heterogeneous. This was followed by an appropriate Student's t test.

RESULTS

Mortality

There was no incidence of any treatment related mortality amongst the rats exposed to bergacyn *(*Lot No. 1701/16*)* at 50 mg, 100 mg and 150 mg/kg body weight. All treated animals survived throughout the treatment period of 28 days and also during recovery period (Table4).

Clinical Signs

In animals treated at and up to the dose 150 mg/kg, the daily general clinical examinations, weekly detailed clinical examinations and the functional observations conducted in the fourth week of the study did not reveal any remarkable and treatment-related incidence of clinical abnormalities. Also no findings, indicative of a neurotoxic potential of the test article, were encountered during these examinations (Table 5 and 6).

Body Weight

Body weight gain by rats treated at 50 mg, 100 mg and 150 mg/kg was found to be comparable to that found in the control rats throughout the treatment period. Also during recovery period, the weight gain by rats from high dose group was found to be comparable to that by the control group rats (Table 7 and 7a).

Food Consumption

The values of average daily food consumption by rats exposed to Bergacyn *(Lot No.* 1701/16*)* at 50, 100 and 150 mg/kg remained very close to the values registered for the control group (G1+1l R) throughout the 28 days of Bergacyn treatment. (Table8).

Furthermore, the average daily food consumption per rat per day, computed over the period of 4 weeks by rats receiving Bergacyn*(*Lot No*.* 1701/16*)* at 150 mg/kg was 96,7% of control values and therefore very similar to the average food intake by vehicle control rats (100%). After cessation of treatment the values of food intake during the recovery period were found to be comparable among the vehicle control and the high dose groups.

Clinical Pathology

Clinical chemistry

The test article Bergacyn(Lot No. 1701/16) up to the dose level of 150 mg/kg, did not induce any changes in the plasma levels of total protein, albumin, alanine aminotransferase, urea, alkaline phosphatase, gamma glutamyltraspeptidase, glucose, creatinine, cholesterol, sodium and potassium in male and female rats at termination of thetreatment.

Values of total protein, albumin, alanine aminotransferase, alkaline phosphatase, glucose, urea, creatinine, cholesterol, sodium and potassium of rats, exposed to Bergacyn (Lot No. 1701/16) at 150 mg/kg, were found to be comparable with those of the control rats at the end of recovery period (Table 10 and 10a).

Haematology

At the end of treatment period, the group mean values of haematological parameters such as haemoglobin, hematocrite, total and differential leucocyte counts, total red blood cells (RBC) count, RBC indices, platelet count and clotting time of rats, exposed to Bergacyn (Lot No. 1701/16) at and up to the level of 150 mg/kg, were found to be comparable with those of the control animals.

Values of haemoglobin, hematocrite, total RBC count, leucocyte counts (WBC), platelet count and clotting time of male and female rats exposed to Bergacyn (Lot No.1701/16) at 150 mg/kg were found to be comparable with those of the control rats at the end of recovery period. (Table 9 and 9a).

Organ Weights

The values of absolute and relative weights of kidneys, liver, adrenals, testes, epididymides, thymus, spleen, brain and heart of male and female rats treated with Bergacyn (Lot No. 1701/16) at and up to 150 mg/kg were found to be comparable to those of the control group rats at termination of the treatment.

Also, the values of absolute and relative weights of kidneys, liver, adrenals, testes, epididymides, thymus, spleen, brain and heart of male and female rats treated with Bergacyn (Lot No. 1701/16) at 150 mg/kg were found to be comparable with those of the control rats at the end of the recovery period (Table 11 and 11a).

Gross Pathology and histopathology

Bergacyn (Lot No. 1701/16) at and up to the dose level of 150 mg/kg, did not induce any remarkable and treatment related pathological alterations in any of the tissues of exposed rats, as evident at the detailed necropsy examination carried out at termination of the study

CONCLUSION

The present study was performed in order to check the potential toxicity and reversibility of the daily oral administration for 30 consecutive days of BPF and Cynara Cardunculus extract (Bergacyn, Lot No. 1701/16) to groups of ten male Wistar rats and ten female Wistar rats for each dose, at doses of 0, 50, 100 and 150 mg/kg.

The findings of this study were as follows:

- Nomortalityatanduptothedoseof150 mg/kg of the test article was found throughout the study;
- No incidence of significant and treatment-related clinical abnormalities at and up to the dose of 150 mg/kg of the test article was found throughout the study;
- No sign of neurotoxicity in the behavioral and functional tests performed on animals treated at and up to the dose of 150 mg/kg of Bergacyn;
- No significant changes in body weight related to the administration of different doses of the test article and vehicle.
- No significant reduction throughout the 28 days treatment in the daily food consumption was found in rats treated with Bergacyn or vehicle.
- No dose-dependent effect on the haematological parameters of rats treated at and up to the dose of 150 mg/kg was registered.
- No effect on the clinical chemistry parameters of rats treated at and up to the dose of 150 mg/kg was observed;
- No significant alterations in the organ weights of rats treated at and up to the level of 150mg/kg;
- No significant and treatment-related gross and microscopic pathological alterations was found in the tissues of rats treated at and up to the level of 150mg/kg.

Based on the findings of this study, the no observable adverse effect level (NOAEL) of BPF and Cynara Cardunculus extract (Bergacyn, Lot No.1701/16) in Wistar rats, following oral administration for 30 days was found to be more than 150 mg/kg body weight.

TABLE 4

SURVIVAL DATA

Group & Dose (mg/kg)	Gl&GlR	G2	G3	G4 & G4R
	0	50	100	150
Week	No. of surviving rats / No. of rats treated			
1	40/40	20/20	20/20	40/40
2	40/40	20/20	20/20	40/40
3	40/40	20/20	20/20	40/40
4	40/40	20/20	20/20	40/40
Reversal Period				
Group & Dose (mg/kg)	GlR		G4R	
	0		150	
Week 4	20/20		20/20	

TABLE 5

SUMMARY OF CLINICAL SIGNS

Group	G1 & G1R<br>Control	G2<br>Low	G3<br>Intermediate	G4 & G4R<br>High
**Dose (mg/kg)**	**0**	**50**	**100**	**150**
**Clinical Finding**	**Incidence<br>(No. of animals with findings / No. of animals treated)**			
**No abnormality detected**	40/40	20/20	20/20	40/40

TABLE6

SUMMARY OF FUNCTIONAL OBSERVATIONS

Period: Day 30

Group	G1 & G1R<br>Control	G2<br>Low	G3<br>Intermediate	G4 & G4R<br>High
**Dose (mg/kg)**	**0**	**50**	**100**	**150**
**No functional abnormalities detected**	40/40	20/20	20/20	40/40

TABLE 7

SUMMARY OF BODY WEIGHT CHANGES (g) – **MALE RATS**

Group and Dose (mg/kg)			Week 0	1	2	3	4
			Weight (g)				
G1&G1R	Control 0	Mean	243,48	264,04	284,78	305,35	312,47
		± SD	12,75	11,95	14,01	12,76	12,06
G2	Low 50	Mean	241,35	254,18	279,94	301,63	308,63
		± SD	11,34	11,60	15,15	12,55	14,15
G3	Intermediate 100	Mean	249,89	268,59	288,71	309,44	317,02
		± SD	11,85	13,80	11,77	11,95	11,85
G4&G4R	High 150	Mean	241,55	264,24	288,74	307,20	315,83
		± SD	14,08	13,73	12,52	11,73	14,63

N= 20 for each group

TABLE 7a

SUMMARY OF BODY WEIGHT CHANGES (g) – **FEMALE RATS**

Group and Dose (mg/kg)			Week				
			0	1	2	3	4
			Weight (g)				
G1&G1R	Control 0	Mean	245,40	265,04	280,78	304,35	310,47
		± SD	12,70	11,90	14,00	12,71	12,06
G2	Low 50	Mean	240,35	255,18	280,94	305,63	310,63
		± SD	11,35	11,56	15,20	12,60	14,20
G3	Intermediate 100	Mean	250,89	268,65	289,71	310,45	310,02
		± SD	11,85	13,86	11,79	11,99	11,85
G4&G4R	High 150	Mean	244,55	264,60	290,74	309,20	315,83
		± SD	14,10	13,75	12,56	11,75	14,63

N= 20 for each group

TABLE 8

FOOD CONSUMPTION – **MALE RATS**

Animal ID	Group and Dose (mg/kg)		Week 0	Week 1	Week 2	Week 3	Week 4	Treatment Period Average	Recovery Period Average
**G1 &G1R**	**Control**	Average	20,67	19,49	21,41	22,05	26,53	22,78	27,33
**G2**	**Low 50**	Average	21,52	21,31	21,84	23,33	23,65	22,12	22,31
		% of Control	104,21	114,24	96,70	112,21	123,84	108,85	109,99
**G3**	**Intermediate 100**	Average	21,41	22,48	23,76	24,82	26	23,88	28,66
		% of Control	105,61	109,23	110,08	98,74	121,45	107,05	128,46
**G4 & G4R**	**High 150**	Average	21,95	22,80	23,97	24,51	26	23,93	28,72
		% of Control	98,15	104,75	108,06	109,44	109,55	105,48	126,58

TABLE 8a
FOOD CONSUMPTION – **FEMALE RATS**

Animal ID	Group and Dose (mg/kg)		Week					Treatment Period Average	Recovery Period Average
			0	1	2	3	4		
G1 &G1R	Control	Average	20,70	19,52	23,41	22,07	26,55	22,78	27,33
G2	Low 50	Average	21,55	21,32	21,86	23,37	23,66	22,12	22,31
		% of Control	105,21	114,30	96,72	112,22	123,87	108,85	109,99
G3	Intermediate 100	Average	21,41	22,48	23,78	24,86	26,01	23,88	28,66
		% of Control	105,65	110,23	110,08	98,78	121,45	107,05	128,46
G4 & G4R	High 150	Average	21,95	22,80	24	24,53	26	23,93	28,72
		% of Control	98,15	105,75	108,06	109,50	109,55	105,48	126,58

TABLE 9

SUMMARY OF HEMATOLOGICAL DATA – **MALE RATS**

Group and Dose (mg/kg)			Hb (g/dl)	HTC (%)	RBC (x 10^6/ml)	WBC (x 10^3/ml)	Platelets (x 10^3/ml)
GI	Control	Mean	13,93	42,62	6,93	4,89	392,08
		± SD	0,40	2,2	0,408	0,204	162,83
		n	20	20	20	20	20
GIR	Controlreversibility	Mean	13,49	40.9	5,76	5,25	383,4
		± SD	0,46	2,85	0.72	0,41	19,26
		n	20	20	20	20	20
G2	Low 50	Mean	13,28	39,06	6,59	4,32	372,6
		± SD	0,40	3,46	0,61	0,51	15,96
		n	20	20	20	20	20
G3	Intermediate 100	Mean	14,42	40,39	7	4,53	361,4
		± SD	0,55	3,57	0,41	0,41	18,74
		n	20	20	20	20	20
G4	High 150	Mean	13,90	43,4	7,21	5,45	353,90
		± SD	0,52	3,06	0,92	0,72	19,67
		n	20	20	20	20	20
G4R	High 150 reversibility	Mean	14,62	44,16	5,45	4,22	362,7
		± SD	0,46	3,57	0,82	0,72	16,06
		n	20	20	20	20	20

TABLE 9a

SUMMARY OF HEMATOLOGICAL DATA – **FEMALE RATS**

Group and Dose (mg/kg)			Hb (g/dl)	HTC (%)	RBC (x 10^6/ml)	WBC (x 10^3/ml)	Platelets (x 10^3/ml)
GI	Control	Mean	13,96	42,66	6,97	4,90	392,10
		± SD	0,40	2,2	0,408	0,204	162,83
		n	20	20	20	20	20
GIR	Controlreversibility	Mean	13,48	40,8	5,78	5,30	383,33
		± SD	0,46	2,85	0.72	0,41	19,26
		n	20	20	20	20	20
G2	Low 50	Mean	13,29	39,09	6,58	4,36	372,8
		± SD	0,40	3,46	0,61	0,51	15,96
		n	20	20	20	20	20
G3	Intermediate 100	Mean	14,46	40,40	7,3	4,55	361,43
		± SD	0,55	3,57	0,41	0,41	18,74
		n	20	20	20	20	20
G4	High 150	Mean	13,93	43,42	7,32	5,46	353,90
		± SD	0,52	3,06	0,92	0,72	19,67
		n	20	20	20	20	20
G4R	High 150 reversibility	Mean	14,65	44,19	5,47	4,22	362,71
		± SD	0,46	3,57	0,82	0,72	16,06
		n	20	20	20	20	20

TABLE 10

SUMMARY OF CLINICAL CHEMISTRY DATA – **MALE RATS**

Group and dose (mg/kg)			Total protein (g/dl)	Albumin (g/dl)	ALT (IU/L)	Cholesterol (mg/dl)	Triglycerides (mg/dl)	ALP (IU/L)	GGT (IU/L)	Glucose (mg/dl)	Creatinin (mg/dl)	Urea (mg/dl)	Sodium (mmol/L)	Potassium (mmol/L)
GI	Control	Mean	5,25	3,29	35,87	54,09	82,71	94,14	1,36	78,4	0,40	36,5	135,94	4,3
		± SD	0,38	0,22	3,17	9,89	16,17	10,71	0,51	8,62	0,019	2,8	8,68	0,8
		n	20	20	20	20	20	20	20	20	20	20	20	20
GIR	Control reversibility	Mean	5,07	3,22	34,74	47,43	70,85	88,84	1,12	88,2	0,34	41,5	131,8	4,84
		± SD	0,41	0,18	2,87	11,85	18,32	22,74	0,30	6,07	0,03	3,23	9,99	0,59
		n	20	20	20	20	20	20	20	20	20	20	20	20
G2	Low 50	Mean	4,31	3,07	36,08	35,47	60,56	104,75	1,53	83,59	0,50	35,5	141,9	4,35
		± SD	0,35	0,25	13,94	13,32	15,19	22,54	0,61	8,72	0,05	4,44	7,47	0,69
		n	20	20	20	20	20	20	20	20	20	20	20	20
G3	Intermediate 100	Mean	4,89	3,51	32,18	29,59	49,88	96,08	1,74	87,2	0,37	35,4	136,6	5,25
		± SD	0,52	0,31	4,61	11,27	17,83	13,56	0,51	7,15	0,049	3,93	8,38	0,90
		n	20	20	20	20	20	20	20	20	20	20	20	20
G4	High 150	Mean	4,77	3,18	35,07	32,43	42,72	89,96	1,33	90,06	0,37	39,07	140,89	5,15
		± SD	0,57	0,34	3,99	9,6	18,71	15,6	0,41	9,99	0,049	4,34	8,88	1,31
		n	20	20	20	20	20	20	20	20	20	20	20	20
G4R	High 150 reversibility	Mean	5,51	3,41	37,31	33,9	51,15	93,73	1,64	82,71	0,39	40,5	142,71	4,24
		± SD	0,30	0,37	4,71	11,07	15,2	18,66	0,30	8,82	0,07	4,44	7,87	0,9
		n	20	20	20	20	20	20	20	20	20	20	20	20

TABLE 10a

SUMMARY OF CLINICAL CHEMISTRY DATA – **FEMALE RATS**

Group and dose (mg/kg)			Total protein (g/dl)	Albumin (g/dl)	ALT (IU/L)	Cholesterol (mg/dl)	Triglycerides (mg/dl)	ALP (IU/L)	GGT (IU/L)	Glucose (mg/dl)	Creatinin (mg/dl)	Urea (mg/dl)	Sodium (mmol/L)	Potassium (mmol/L)
GI	Control	Mean	5,24	3,28	35,75	53,95	82,50	93,93	1,36	78,46	0,39	36,19	135,53	4,68
		± SD	0,36	0,21	3,16	9,86	16,12	10,67	0,50	8,59	0,018	2,79	8,65	0,79
		n	20	20	20	20	20	20	20	20	20	20	20	20
GIR	Control reversibility	Mean	5,05	3,21	34,63	47,48	70,63	88,57	1,11	87,93	0,33	41,37	131,40	4,82
		± SD	0,43	0,17	2,86	11,81	18,26	22,67	0,29	6,05	0,029	3,22	9,96	0,58
		n	20	20	20	20	20	20	20	20	20	20	20	20
G2	Low 50	Mean	4,29	3,06	35,97	35,36	60,37	104,43	1,52	83,33	0,49	35,39	141,47	4,33
		± SD	0,34	0,24	13,89	13,28	15,14	22,47	0,60	8,69	0,049	4,42	7,44	0,68
		n	20	20	20	20	20	20	20	20	20	20	20	20
G3	Intermediate 100	Mean	4,87	3,49	32,083	29,50	49,73	95,79	1,73	86,93	0,36	35,29	136,19	5,23
		± SD	0,51	0,30	4,59	11,23	17,77	13,51	0,50	7,12	0,048	3,91	8,35	0,89
		n	20	20	20	20	20	20	20	20	20	20	20	20
G4	High 150	Mean	4,75	3,17	34,96	32,33	42,59	89,69	1,32	89,78	0,36	38,95	140,46	5,13
		± SD	0,56	0,33	3,97	9,57	18,65	15,55	0,40	9,96	0,048	4,32	8,85	1,30
		n	20	20	20	20	20	20	20	20	20	20	20	20
G4R	High 150 reversibility	Mean	5,51	3,39	37,19	33,79	50,19	93,44	1,63	82,46	0,38	40,37	142,28	4,22
		± SD	0,29	0,36	4,69	11,03	15,15	18,60	0,29	8,79	0,06	4,42	7,84	0,89
		n	20	20	20	20	20	20	20	20	20	20	20	20

TABLE 11

SUMMARY OF ABSOLUTE ORGANWEIGHTS (g) – **MALE RATS**

Animal ID	Group and Dose (mg/kg)		Organ Weights								
			Adrenals	Spleen	Thymus	Heart	Kidneys	Liver	Testes	Epididymides	Brain
GI	Control	Mean	0,132	0,595	0,295	1,012	1,830	9,423	3,115	1,012	1,732
		+ S.D.	0,02	0,14	0,12	0,09	0,27	0,45	0,21	0,07	0,19
		n	20	20	20	20	20	20	20	20	20
GIR	Control Reversibility	Mean	0,142	0,613	0,273	1,056	2,145	9,332	3,265	1,111	1,815
		+ S.D.	0,06	0,21	0,11	0,10	0,31	0,71	0,31	0,38	0, 10
		n	20	20	20	20	20	20	20	20	20
G2	Low 50	Mean	0,155	0,578	0,268	1,032	2,167	9,515	3,352	1,212	1,822
		+ S.D.	0,06	0,20	0,15	0,09	0,31	0,78	0,36	0,30	0,16
		n	20	20	20	20	20	20	20	20	20
G3	Intermediate 100	Mean	0,172	0,625	0,307	1,022	2,111	9,289	3,418	1,295	1,791
		+ S.D.	0,09	0,18	0,18	0,11	0, 34	0,81	0,50	0,28	0,19
		n	20	20	20	20	20	20	20	20	20
G4	High 150	Mean	0,121	0,604	0,299	1,071	2,191	9,415	3,611	1,118	1,818
		+ S.D.	0,08	0,13	0,11	0,08	0, 26	0,51	0,41	0,29	0,09
		n	20	20	20	20	20	20	20	20	20
G4R	High 150 Reversibility	Mean	0,186	0,611	0,282	1,095	2,168	9,321	3,398	1,125	1,841
		+ S.D.	0,08	0, 13	0,140	0,16	0,31	0,61	0,40	0,30	0,16
		n	20	20	20	20	20	20	20	20	20

TABLE 11a

SUMMARY OF ABSOLUTE ORGANWEIGHTS (g) **– FEMALE RATS**

Animal ID	Group and Dose (mg/kg)		Organ Weights								
			Adrenals	Spleen	Thymus	Heart	Kidneys	Liver	Testes	Epididymides	Brain
GI	Control	Mean	0,131	0,593	0,294	1,008,96	1,824	9,394	3,105	1,008	1,732
		+ S.D.	0,019	0,13	0,11	0,89	0,26	0,44	0,20	0,069	0,19
		n	20	20	20	20	20	20	20	20	20
GIR	Control Reversibility	Mean	0,141	0,611	0,272	1,052	2,138	9,304	3,255	1,107	1,815
		+ S.D.	0,059	0,20	0,10	0,09	0,30	0,70	0,30	0,37	0, 10
		n	20	20	20	20	20	20	20	20	20
G2	Low 50	Mean	0,154	0,576	0,267	1,028	2,160	9,486	3,341	1,291	1,822
		+ S.D.	0,059	0,19	0,14	0,089	0,30	0,77	0,35	0,28	0,16
		n	20	20	20	20	20	20	20	20	20
G3	Intermediate 100	Mean	0,171	0,623	0,306	1,018	2,104	9,261	3,407	1, 291	1,791
		+ S.D.	0,089	0,179	0,179	0,10	0, 33	0,80	0,49	0,28	0,19
		n	20	20	20	20	20	20	20	20	20
G4	High 150	Mean	0,120	0,602	0,298	1,066	2,184	9,386	3,600	1,118	1,818
		+ S.D.	0,079	0,12	0,10	0,079	0, 25	0,50	0,40	0,29	0,09
		n	20	20	20	20	20	20	20	20	20
G4R	High 150 Reversibility	Mean	0,185	0,609	0,281	1,091	2,161	9,293	3,387	1,125	1,841
		+ S.D.	0,079	0,12	0,139	0,15	0,30	0,60	0,39	0,30	0,16
		n	20	20	20	20	20	20	20	20	20

TABLE 12

SUMMARY OF GROSS PATHOLOGICAL FINDINGS

Group		G1	G2	G3	G4
**Dose (mg I kg)**		**0**	**50**	**100**	**150**
**Number of animals:**		20	20	20	20
**No abnormality detected**		20	20	20	20
**Lungs**	- acutecongestion		-	-	-
	- infiltration of roundcells	-	-	-	-
	- abscess/suppurative<br>- bronchopneumonia	-	-	-	-
	- emphysema	-	-	-	-
**Kidneys**	- interstitial infiltration of roundcell	-	-	-	-
	- tubular cellhyperplasia	-	-	-	-
	- hydronephrosis	-	-	-	-
**Spleen**	- lymphoidhyperplasia	-	-	-	-
**Testes**	- atrophic seminiferoustubules	-	-	-	-
**Epididymis**	- atrophic epididymalducts	-	-	-	-
**Liver**	- cyst	-	-	-	-
	- acutecongestion	-	-	-	-
	- infiltration of roundcells	-	-	-	-
	- necrosis	-	-	-	-
**Duodenum**	- exfoliation of mucosal epithelium	-	-	-	-
**Jejunum**	- lymphoidhyperplasia	-	-	-	-
	- exfoliation of mucosalepithelium	-	-	-	-
	- exfoliation of mucosa!epithelium	-	-	-	-
**Heart**	- fibrinouspericarditis	-	-	-	-

TABLE 13
SUMMARY OF HISTOPATHOLOGICAL FINDINGS

Group	G1	G1R	G2	G3	G4	G4R
**Dose (mg *I*kg)**	0	0	50	100	150	150
**No abnormality detected**	20/20	20/20	20/20	20/20	20/20	20/20
**Lung – Reddening, abscess**	-	-	-	-	-	-
**Kidneys – slight pallor, hydronephrosis**	-	-	-	-	-	-
**Spleen - slightly enlarged**	-	-	-	-	-	-
**Testes - slightly undersized**	-	-	-	-	-	-
**Liver - Enlarged, sight pllor**	-	-	-	-	-	-
**Heart - Dilatation, fibrosis**	-	-	-	-	-	-

REFERENCES

1. Nogata Y, Sakamoto K, Shiratsuchi H, Ishii T, Yano M, et al. (2006) Flavonoid composition of fruit tissues of citrus species. BiosciBiotechnolBiochem 70:178-192.
2. Jeong YJ, Choi YJ, Choi JS, Kwon HM, Kang SW, et al. (2007) Attenuation of monocyte adhesion and oxidised LDL uptake in luteolin-treated human endothelial cells exposed to oxidised LDL. Br J Nutr 97:447-457.
3. Yu J, Wang L, Walzem RL, Miller EG, Pike LM, et al. (2005) Antioxidant activity of citrus limonoids, flavonoids, and coumarins. J Agric Food Chem 53:2009-2014.
4. Di Donna L, De Luca G, Mazzotti F, Napoli A, Salerno R, et al. (2009) Statin-like principles of bergamot fruit (Citrus bergamia): isolation of 3-hydroxymethylglutaryl flavonoid glycosides. J Nat Prod 72:1352-1354.
5. Mollace V, Sacco I, Janda E, Malara C, Ventrice D, et al. (2011) Hypolipemic andhypoglycaemic activity of bergamot polyphenols: from animal models to human studies. Fitoterapia 82:309-316.
6. Gliozzi M, Walker R, Muscoli S, Vitale C, Gratteri S, et al. (2013) Bergamot polyphenolic fraction enhances rosuvastatin-induced effect on LDL-cholesterol, LOX-1 expression and protein kinase B phosphorylation in patients with hyperlipidemia. Int J Cardiol 170: 140-145.
7. Gliozzi M, Carresi C, Musolino V, Palma E, Muscoli C et al. (2014) The effect of bergamot-derived polyphenolic fraction on LDL small dense particles and non alcoholic fatty liver disease in patients with MS. Advances in BiologicalChemistry.
8. Leighton F, Miranda-Rottmann S, Urquiaga I (2006) A central role of eNOS in the protective effect of wine against metabolic syndrome. Cell BiochemFunct 24:291-298.
9. Mollace V, Ragusa S, Sacco I, Muscoli C, Sculco F, et al. (2008) The protective effect of bergamot oil extract on lecitine-like oxyLDL receptor-1 expression in balloon injury- related neointima formation. J CardiovascPharmacolTher 13:120-129.
