# Supplementary material for: Randomized Clinical Trial: Bergamot Citrus and Wild Cardoon Reduce Liver Steatosis and Body Weight in Non-diabetic Individuals Aged Over 50 Years
Source: Front Endocrinol (Lausanne). 2020 Aug 11;11:494. doi: 10.3389/fendo.2020.00494 (PMC7431622; doi:10.3389/fendo.2020.00494)
Supplement: Supplementary file 1 [file Data_Sheet_1.zip › Sup. material. 2- BC 90 days toxicology.pdf]

FINAL REPORT

Repeated dose 90-days Oral (Gavage) Toxicity of a composition of Bergamot Poliphenolic Fraction (BPF) and Cynara Carduncululus extract (Bergacyn) in Wistar Rats

Test Article:
Bergacyn composition

Sponsor:
Herbal & Antioxidant Derivatives srl (HEAD)
C.da Chiusi – 89032 BIANCO (RC)

Testing Facility:
Nutramed Scarl
Complesso Ninì Barbieri,
Roccelletta di Borgia (CZ)

Study N.
152-00041

Author:
Sara Paone, PhD

Study Completion Date: 30 october 2016

NUTRAMED S.C.A.R.L.
Complesso Ninì Barbieri
88021 Roccelletta di Borgia (Cz) – Italy
Tel. 09613695732
Mail: massimo.fini@sanraffaele.it

TABLE OF CONTENTS

TABLE OF CONTENTS 2
COMPLIANCE STATEMENT 3
QUALITY ASSURANCE STATEMENT 4
SIGNATURE PAGE 5
STUDY PERSONNEL 6
STUDY TIMETABLE 7
SUMMARY 8
INTRODUCTION 10
MATERIALS AND METHODS 11
Test and control articles 11
Test animals & Husbandry 11
Experimental design 13
Administration of the test article 14
Pathology 16
Terminal studies 18
Statistical analysis 18
RESULT 19
Mortality 19
Clinical Signs 19
Body Weights 19
Food Consumption 19
Clinical Pathology 20
Haematology 20
Clinical Chemistry 20
Organ Weights 20
Gross Pathology 20
CONLUSION 21
SUMMARY DATA (TABLES 4-13) 22
REFERENCES 32

COMPLIANCE STATEMENT

Repeated dose 90-day Oral (Gavage) Toxicity of Bergamot Polyphenolic Fraction (BPF) and Cynara Cardunculus extract (Bergacyn) in Wistar Rats

This study was conducted in compliance with the EU Directive 2004/9/EC and Directive 2004/9/EC for Good Laboratory Practice Guidelines (GLP) and with OECD Guidelines for Repeated Dose 90-day Oral Toxicity Study in Rodents (n. 408 Adopted 21st september 1998)

Study Director: Date

Sara Paone, PhD, DABT 10/11/2016

QUALITY ASSURANCE STATEMENT

Repeated dose 90-day Oral (Gavage) Toxicity of Bergamot Polyphenolic Fraction (BPF) and Cynara Cardunculus extract (Bergacyn) in Wistar Rats

This study, n. 132-0437C0 entitled “Repeated dose 90-day Oral (Gavage) Toxicity of Bergamot Polyphenolic Fraction (BPF) and Cynara Cardunculus extract (Bergacyn) in Wistar Rats with a 2-Week Recovery” was inspected/audited by Quality Assurance in accordance with San Raffaele IRCCS Guidelines and OECD Good Laboratory Practice Regulations. All findings were reported to the Study Director and Testing Facility Management.

Date of Inspection	Phase of the Study	Date of Reporting
June 15, 2016	Randomization and grouping of animals	June 15, 2016
July 6, 2016	Administration of Test Article; Clinical Examination & Animal Weighing	June 5, 2016
July 6, 2016	Administration of Test Article;	July 6, 2016
July 20, 2016	Clinical Examination & Food Weight measurement Clinical Examination, Functional	July 20, 2016
July 22, 2016	Observations Terminal Necropsy	July 22 2016
July 28, 2016	Terminal Necropsy: Reversal Groups	July 28, 2016
October 30, 2016	Report Audit	October 30, 2016

Action has been taken in response to all items listed by Quality Assurance. It is concluded that the final report accurately reflects NutraMed SCARL- San Raffaele Standard Operating Procedures and the raw data for this study.

MICHELANGELO Iannone
Sr. Manager Quality Assurance

Date

10.11.2016

SIGNATURE PAGE

Repeated dose 90-days Oral (Gavage) Toxicity of a composition of Bergamot Poliphenolic Fraction (BPF) and Cynara Cardunculus extract (Bergacyn) in Wistar Rats

Author

05/01/2016

Sara Paone, PhD, DABT | Date
Study Director

STUDY PERSONNEL

Study Director:	Sara Paone, PhD
Toxicology Associate:	Luigi Tucci, PhD
Report Associate:	Luca Gareri, PhD
Head Technicians:	Giovanni Politi, BS

Supervisor, Necropsy:	Francesca Oppedisano, BS
Manager, Formulations:	Roberta Macri', BS
Pathologist:	Caterina Criaco, PhD
Toxicology:	Federica Scarano, PhD, DABT Director, Laboratory
Animal Medicine:	Nicola Costa, PhD

Sponsor: Herbal & Antioxidant Derivatives srl (HEAD)
Via Ronchi – POLISTENA (RC)

Sponsor Representative/Study Monitor: Giuseppe Lombardo

STUDY TIMETABLE

Study Initiation Date:	June 15, 2016
Experimental Start Date:	July 06, 2016
Receipt of Animals:	June 14, 2016
Randomization of Animals:	June 15, 2016
First Day of Dosing:	July 06, 2016
Last Day of Dosing:	October 01 2016
Necropsy: Terminal Sacrifice	October 30, 2016
Experimental Completion Date:	October 30, 2016
Study Completion Date:	November 11, 2016

SUMMARY

Repeated dose 90-days Oral (Gavage) Toxicity of a composition of Bergamot Poliphenolic Fraction (BPF) and Cynara Cardunculus extract (Bergacyn) in Wistar Rats

Male and female Wistar rats (10 male and 10 female for each group) received orally BPF and Cynara Cardunculus extract (Bergacyn) (Lot. No. 1701/16) by gastric gavage daily at the doses of 50 mg/kg, 100 mg/kg or 150 mg/kg body weight for 28 consecutive days and were sacrificed on day 29 to evaluate its toxicity. Bergacyn was diluted in vehicle (in demineralised water) at the concentration 10, 40, 200 mg/ml respectively to allow a constant dose of the vehicle (max 2 ml/100 g body weight) to be administered together with the test article. Concurrent control groups receiving vehicle at 2 ml/100 g body weight were also maintained. Additionally, satellite groups of six rats receiving vehicle at 2 ml/100 g body weight and six rats receiving Bergacyn at the highest dose of 150 mg/kg body weight were further observed for a period of 14 days following 28 days exposure, for the assessment of reversibility, persistence or delayed occurrence of toxicity.

The rats were examined daily for signs of toxicity, morbidity and mortality. They were subjected to detailed clinical examination before initiation of the study and weekly thereafter during the exposure period, reversal period, and at termination. In the first, and fourth week of exposure they were additionally examined for assessment of sensory reactivity, assessment of grip strength and motor activity. Body weight and food consumption were recorded weekly. Laboratory investigations were performed on blood samples at termination of the study. All animals sacrificed terminally were subjected to a detailed necropsy and weights of certain organs were recorded.

Histopathological evaluation showed that no organ alterations were encountered. There was no incidence of treatment related mortality in rats exposed to Bergacyn at 50, 100 and 150 mg/kg body weight. The test article did not induce any remarkable and treatment related clinical abnormalities in rats treated at and up to the dose of 150 mg/kg. No mortality or abnormal clinical signs were observed in animals treated with vehicle. Also, the functional observations conducted throughout the time of exposure did not reveal any neurotoxic effects of the test article up to its highest level of exposure.

The hematological parameters of hemoglobin, hematocrit and total RBC counts, leucocyte counts, platelet count and clotting time of rats exposed to the test article at and up to the

level of 150 mg/kg were found to be comparable to those of the vehicle control animals at termination of the treatment and also at the end of recovery period.

The test article, at and up to the level of 150 mg/kg, did not alter the plasma levels of total protein, albumin, alanine aminotransferase (ALT), alkaline phosphatase (ALP), gamma glutamyl traspeptidase (GGT), glucose, creatinine, urea, sodium and potassium in Wistar rats. The values of absolute and relative weights of liver, kidneys, adrenals, spleen, heart, brain, testes, thymus and epididymides of male and female rats treated with Bergacyn at and up to 150 mg/kg were found to be comparable with those of the control rats at the end of treatment period and also at the end of recovery period. Bergacyn did not induce any treatment related gross pathological alterations in treated rats at and up to the dose of 150 mg/kg. Based on the findings of this study the no observable adverse effect level (NOAEL) of Bergacyn in Wistar rats, following oral administration for 28 days, was found to be more than 150 mg/kg bodyweight.

The test article, at and up to the level of 1000 mg/kg, did not alter the plasma levels of total protein, albumin, alanine aminotransferase (ALT), alkaline phosphatase (ALP), gamma glutamyl traspeptidase (GGT), glucose, creatinine, urea, sodium and potassium in Wistar rats. The values of absolute and relative weights of liver, kidneys, adrenals, spleen, heart, brain, testes, thymus and epididymides of male rats treated with Bergacyn, at and up to 1000 mg/kg were found to be comparable with those of the control rats at the end of treatment period and also at the end of recovery period. Bergacyn did not induce any treatment related gross pathological alterations in treated rats at and up to the dose of 1000 mg/kg. Based on the findings of this study the no observable adverse effect level (NOAEL) of Bergacyn in Wistar rats, following oral administration for 90 days, was found to be more than 1000 mg/kg body weight.

INTRODUCTION

Bergamot (Citrus bergamia) is an endemic plant of the Calabrian region in Southern Italy with a unique profile of flavonoid and flavonoid glycosides present in its juice and albedo, such as neoeriocitrin, neohesperidin, naringin, rutin, neodesmin, rhoifolin and poncirin. Bergamot differs from other Citrus fruits not only because of the composition of its flavonoids, but also because of their particularly high content. Among them naringin, present also in grapefruit, has already been reported to be active in animal models of atherosclerosis, while neoeriocitrin and rutin have been shown to inhibit LDL oxidation. Importantly, bergamot juice is rich in neohesperidosides of hesperetin and naringenin, such as melitidine and brutieridine. These flavonoids possess a 3-hydroxy-3-methylglutaryl moiety with a structural similarity to the natural substrate of HMG-CoA reductase and exhibit statin-like proprieties.

The cultivation of artichoke comes from the mists of the past. The plant seems to be native to Ethiopia or Egypt, whence it was brought to other Mediterranean areas of civilization. The common name of the plant derives from the Arabic “alharsuf” (“ground-thorn”) which in the different countries, throughout the centuries, became “alcachofa” in Spanish, “alcachorfa” in Portuguese, “artichaut” in French. In English language, it is also known as “globe artichoke”. The scientific name, *Cynara* derives from a Roman consolidated use to manure the plant with ash *“cinere”* in Latin.

The plant is always very appreciated as a food, but recently, the therapeutic potential of Cynara Cardunculus has also been investigated in human studies.

In particular, experimental and epidemiological studies have demonstrated that Cynara Cardunculus extract (Bergacyn) has choleretic, cholagogue, diuretic, laxative, anti-gout activities; it also ameliorates serum lipemic profile. The pharmacological and therapeutic actions of the artichoke in liver disorders have been demonstrated by clinical experiments in which ascertained a marked therapeutic activity, particularly in some patients suffering from different forms of jaundice. Researchers observed a rapid disappearance of salts and biliary pigments from urine, restoration of normal color in faeces, simultaneous clearing of the skin and a considerable diuretic effect. The double action, choleretic and diuretic, of the artichoke, was connected to its high content in potassium and magnesium salts. The choleretic activities of artichoke are to be attributed to the active part of leaves. In addition, also the preparations made from artichoke roots were able to quadruplicate bile secretion, in the same way as those obtained from the leaves.

The purpose of this study was to determine the toxicity of BPF and Cynara Cardunculus

(Bergacyn) in male Wistar rats when administered once daily by oral gavage for 30 consecutive days. The rat was selected because it is the standard species for use in toxicology studies for EMA and FDA Guidelines, and because this study was conducted in accordance with the regulatory guideline; alternatives could not be considered. The oral gavage approach was selected because it is the intended route of administration to human subjects.

METHODS AND MATERIALS

Test and Control Articles

The neat test and control articles used was Bergamot Polyphenolic Fraction and Cynara Cardunculus extract (Bergacyn) provided by Herbal and Antioxidant Derivatives srl (Bianco (RC), Italy – Lot. No. 1701/16).

Analitical purity controls have been performed via LC-MS detection at the IRC-FSH, University of Catanzaro, Italy as previously described (see ref. 1)

Reserve samples of the test article were taken at IRC-FSH prior to use on this study. The samples were archived at San Raffaele – Centro del Farmaco under the same conditions as the test and control articles.

Any remaining test and control articles were returned to the Sponsor following completion of the study.

Test Animals and Husbandry

<u>**Animals**</u>

Animal information is provided in the Table 1.

Table 1: Animal Information

Parameter	Males
Species and Strain	Wistar rats
Supplier	Charles River
Number of Animals Received	125
Number of Animals Used on Study	120
Age of Animals at First Dose	8 to 9 weeks
Body Weight Range at First Dose	220,5-231,4 g

Animals were acclimated to laboratory conditions for 10 days prior to the first dose and released from quarantine by a staff veterinarian. During the acclimation period, each animal was identified by a temporary number that was recorded on each cage label.

NutraMed Animal Care and Use Committee (IACUC) approved this protocol and found it to be in accordance with provisions of the EU Policy on Humane Care and Use of Laboratory Animals, and the Italian Interagency Research Animal Committee Principles for the Utilization and Care of Research Animals.

Husbandry

Animal husbandry was provided as described in Table 2.

Table 2: Husbandry

**Feed**[a]	Certified Global Harlan Laboratory Rodent Diet
**Water**[b]	Water via an automatic watering system and water bottles
**Bedding**[c]	Certified hardwood bedding
**Caging**	Polycarbonate cages
**Racks**	Stainless steel racks
**Animals Per Cage**	One
**Temperature Range**	64 to 79°F
**Humidity Range**	30 to 70%
**Light Cycle**	12-hour light/12-hour dark, interrupted as necessary for study-related events
**Air Changes**	Minimum of 10 air changes per hour

[a] Feed was analyzed by the manufacturer for concentrations of specified heavy metals, aflatoxin, organophosphates and specific nutrients

[b]The number was routinely analyzed for contaminants and specific microbs

[c] The bedding was analyzed by the manufacturer for acceptable levels on heavy metals, afatoxins, bacteria, yeasts, and organophosphates prior to certification

Feed and water were provided *ad libitum*. No contaminants were known to be present in the diet, water, or bedding at levels that might have interfered with achieving the objectives of the study.
Environmental controls were set to maintain animal room conditions as shown in Table 2. Actual temperature and relative humidity in the animal room or zone were monitored continuously by a computerized system and manually recorded at least once daily. All environmental parameters were maintained within the protocol requirements.

Experimental Design

Group Assignment and Doses

Groups of six male Wistar rats were administered with Bergacyn *(*Lot. No. 1701/16*)* by oral gavage daily at the doses of 50 mg/kg, 200 mg/kg or 1000 mg/kg body weight for 90 days and were sacrificed on day 91 to evaluate its toxicity. Concurrent control group receiving vehicle at 2 ml/kg (in demineralised water) was also maintained. Additionally, satellite groups of six rats receiving vehicle at 2 ml/kg and the test article at 1000 mg/kg levels were further observed for a period of 14 days following the 90 day exposure, for assessment of reversibility, persistence or delayed occurrence of toxicity. The Groups of animals and the related treatment regimens are displayed in the Table 3

Table 3

Groups of animals used throughout the study

	Group	Dosage (mg/kg)	No.	Male rats IDs	Female rats IDs
**G1**	Control	0	20	SCC1 -SCCS	SCC1F -SCCSF
**G1R**	Control, Reversal	0	20	SRCI1- SRC5	SRCI1F- SRC5F
**G2**	Low	50	20	SCL1 -SCL5	SCL1F -SCL5F
**G3**	Intermediate	100	20	SCM1-SCM5	SCM1F-SCM5F
**G4**	High	150	20	SCH1- SCH5	SCH1F- SCH5F
**G4R**	High, Reversal	150	20	SHR1- SHR5	SHR1F- SHR5F

	Total		120		

Dose Formulation

Bergacyn (Lot. No. 1701/16) was suspended in demineralised water and given orally at doses of 50 mg, 200 mg and 1000 mg/kg for which the concentrations of 10 mg/ml, 40 mg/ml and 200 mg/ml were chosen respectively to give a constant dosage volume rate of 5 ml/kg body weight.
The formulations were freshly prepared on each day of dosing.

Administration of the test article

The animals were dosed by oral gavage at approximately the same time each day where possible, using a 2.5 ml graduated disposable syringe and a stainless steel intubation needle (16 G). The dosage volume administered to each individual rat was adjusted according to its most recently recorded body weight. The control group rats received demineralised water by oral gavage at the same dosage volume of 5 ml/kg body weight. Treatment in this manner was continued once a day, seven days a week, for a total period of 90 days.

Observations

The animals were monitored during the course of treatment for mortality, body weight gain, food consumption and clinical signs of toxicity by general and detailed clinical and functional examinations as listed below. Rats belonging to the reversal group were further observed for a post-treatment period of 14 days to permit evaluation of the persistence or reversibility of any toxic effects, or the occurrence of delayed toxicity.

Mortality

Throughout the study, all animals were checked early on each working day and again in the afternoon to look for dead or moribund animals to allow necropsy examination to be carried out during the working hours of that day.

Body Weight

The weight of each rat was recorded at the time of allocation of the animal to groups, on the day of commencement of treatment, weekly thereafter and at necropsy. Weights of reversal group rats were recorded weekly during post-treatment period and at necropsy.

Food Consumption

The quantity of food consumed by rats in each cage was recorded on the day of commencement of treatment and weekly thereafter. Food intake per rat (for 24 hours) was calculated using the amount of food offered to and left in each cage in each group, and the number of rats in each cage. Food consumption of reversal group rats was recorded weekly during post-treatment period.

Clinical Signs

All signs of poor health, together with any behavioural changes or reaction to treatment were recorded for individual animals.

Dated and signed records of appearance, change and disappearance of clinical signs were maintained on clinical history sheets for individual animals.

General Clinical Examinations

The rats were daily subjected to general cage side clinical examinations, at the same time each day, and at suitable intervals after dosing, considering the peak period of anticipated effects after dosing.

Detailed Clinical Examinations

The rats were subjected to detailed clinical examinations before initiation of the study and weekly thereafter during the study. These were conducted outside the home cage in a standard arena and at the same time, the observers being unaware of the treatment.

Signs noted included, but were not limited to, changes in skin, fur, eyes, and mucous membranes, occurrence of secretions and excretions and autonomic activity such as lacrimation, pupil size, and unusual respiratory pattern.

Changes in gait, posture and response to handling as well as the presence of clonic or tonic movements, stereotypes or bizarre behaviour were also recorded.

Functional Observations

During the thirteen week of exposure, following the detailed clinical examination, animals were additionally examined for assessment of sensory reactivity, assessment of grip strength and motor activity.

PATHOLOGY

Clinical Pathology

Food was removed overnight from animals to be sampled for laboratory investigations. Samples of blood were withdrawn at termination of treatment/recovery period, under carbon dioxide anesthesia, by cardiac puncture. The samples were collected in tubes containing Heparin (for clinical chemistry) and EDTA (for hematology) as an anticoagulant. Blood smears were also made on glass slides.

The estimations that were performed on blood samples have been listed below, together with an abbreviated title (used in Appendices and Tables).

Hematology

Hematological evaluations of the blood samples were performed using an COBAS m 511 Hematology System (Roche). The following parameters were recorded, with their units of measurement as listed below:

- Hemoglobin (Hb) (mg/dl)
- Hematocrite (%) (HCT)
- Total red cell count (Total RBC) ($x10^6$/µl)
- Total white cell count (Total WBC) ($xl0^3$/µl)
- Mean corpuscular volume (MCV) (pg)
- Mean corpuscular hemoglobin (MCH) (fl)
- Mean corpuscular hemoglobin concentration (MCHC) (g/dl)
- Platelet count ($x10^3$/µl).
- Differential WBC counts (% of Total WBC): Neutrophils (N), Eosinophils (E), Lymphocytes (L), Monocytes (M)
- Clotting time measurement (seconds) was performed manually using standard techniques.

Clinical Chemistry

Chemical and biochemical serum parameters were analyzed using a BT 4500 Clinical Chenistry Analyzer, Biotecnica Instruments, Italy, according to manufacturer's instructions. The following parameters were measured, with their units of measurement as listed below:

- Total Protein (g/dl)
- Albumin (g/dl)
- Alanine aminotransferase (ALT) (IU/1)
- Cholesterol (Cholest) (mg/dl)

- Urea (mg/dl)

- Potassium (mmol/1)
- Alkaline phosphatase (ALP) (IU/1)
- Gamma-glutamyl transpeptidase (GGT) (IU/1)
- Triglycerides (mg/dl)
- Glucose (mg/dl)
- Creatinine (mg/dl)
- Sodium (mmol/1)

TERMINAL STUDIES

Necropsy Examination

On completion of 90 days of treatment and/or 14 days of reversal period, all surviving rats were sacrificed by exsanguination (terminal cardiac puncture) under carbon dioxide anaesthesia. All the tissues listed in Table 12, from all animals, were preserved, in 10% neutral buffered formalin.

Organ Weights

The following organs from all animals, killed at the scheduled sacrifices, were dissected free of fat and weighed wet as soon as possible to avoid drying: kidneys, liver, adrenals, testes, epididymides, thymus, spleen, brain, heart.

STATISTICAL ANALYSIS

Statistical analysis of Bergacyn treatment effects on all measured parameters (rat body weights, organ weights, food consumption and on hematology and clinical chemistry results) Bartlett's test was performed on each set of data to ensure that variance of the sets are homogenous. In case of homogenous sets of data ANOVA was performed to determine the treatment effects. Finally, Dunnett's test was employed to establish if any mean value of results from treatment groups (G2, G3, G4+G4R, G4R) was significantly different compared to a mean value of the control group (G1, G1+G1 R, G1R).

In case of heterogeneous data, F test was carried out to determine which pairs of groups

are heterogeneous. This was followed by an appropriate Student's t test.

RESULTS

Mortality

There was no incidence of any treatment related mortality amongst the rats exposed to Bergacyn *(*Lot No. 1701/16*)* at 50 mg, 200 mg and 1000 mg/kg body weight. All treated animals survived throughout the treatment period of 90 days and also during recovery period (Table 4).

Clinical Signs

In animals treated at and up to the dose 1000 mg/kg, the daily general clinical examinations, weekly detailed clinical examinations and the functional observations conducted in the fourth week of the study did not reveal any remarkable and treatment-related incidence of clinical abnormalities. Also no findings, indicative of a neurotoxic potential of the test article, were encountered during these examinations (Table 5 and 6).

Body Weight

Body weight gain by rats treated at 50 mg, 200 mg and 1000 mg/kg was found to be comparable to that found in the control rats throughout the treatment period. Also during recovery period, the weight gain by rats from high dose group was found to be comparable to that by the control group rats (Table 7 and 7a).

Food Consumption

The values of average daily food consumption by rats exposed to Bergacyn *(Lot No.* 1701/16*)* at 50, 200 and 1000 mg/kg remained very close to the values registered for the control group (G1+1l R) throughout the 90 days of Bergacyn treatment. (Table 8 and 8a).

Furthermore, the average daily food consumption per rat per day, computed over the period of 12 weeks by rats receiving Bergacyn *(*Lot No*.* 1701/16*)* at 1000 mg/kg was 96,7% of control values and therefore very similar to the average food intake by vehicle control rats (100%). After cessation of treatment the values of food intake during the recovery period were found to be comparable among the vehicle control and the high dose groups.

Clinical Pathology

Clinical chemistry

The test article Bergacyn (Lot No. 1701/16) up to the dose level of 1000 mg/kg, did not induce any changes in the plasma levels of total protein, albumin, alanine aminotransferase, urea, alkaline phosphatase, gamma glutamyl traspeptidase, glucose, creatinine, cholesterol, sodium and potassium in male rats at termination of the treatment.

Values of total protein, albumin, alanine aminotransferase, alkaline phosphatase, glucose, urea, creatinine, cholesterol, sodium and potassium of rats, exposed to Bergacyn (Lot No. 1701/16) at 1000 mg/kg, were found to be comparable with those of the control rats at the end of recovery period (Table 10 and 10a).

Hematology

At the end of treatment period, the group mean values of hematological parameters such as hemoglobin, hematocrite, total and differential leucocyte counts, total red blood cells (RBC) count, RBC indices, platelet count and clotting time of rats, exposed to Bergacyn (Lot No. 1701/16) at and up to the level of 1000 mg/kg, were found to be comparable with those of the control animals.

Values of hemoglobin, hematocrite, total RBC count, leucocyte counts (WBC), platelet count and clotting time of male rats exposed to Bergacyn (Lot No.1701/16) at 1000 mg/kg were found to be comparable with those of the control rats at the end of recovery period. (Table 9 and 9a)

Organ Weights

The values of absolute and relative weights of kidneys, liver, adrenals, testes, epididymides, thymus, spleen, brain and heart of male rats treated with Bergacyn (Lot No. 1701/16) at and up to 1000 mg/kg were found to be comparable to those of the control group rats at termination of the treatment.

Also, the values of absolute and relative weights of kidneys, liver, adrenals, testes, epididymides, thymus, spleen, brain and heart of male rats treated with Bergacyn (Lot No. 1701/16) at 1000 mg/kg were found to be comparable with those of the control rats at the end of the recovery period (Table 11 and 11a).

Gross Pathology and histopathology

Bergacyn (Lot No. 1701/16) at and up to the dose level of 1000 mg/kg, did not induce any remarkable and treatment related pathological alterations in any of the tissues of exposed

rats, as evident at the detailed necropsy examination carried out at termination of the study

CONCLUSION

The present study was performed in order to check the potential toxicity and reversibility of the daily oral administration for 90 consecutive days of Bergamot Polyphenol Fraction (Bergacyn, Lot No. 1701/16) to groups of six male Wistar rats per dose, at doses of 0, 50, 200 and 1000 mg/kg.

The findings of this study were as follows:

- No mortality at and up to the dose of I000 mg/kg of the test article was found throughout the study;
- No incidence of significant and treatment-related clinical abnormalities at and up to the dose of 1000 mg/kg of the test article was found throughout the study;
- No sign of neurotoxicity in the behavioral and functional tests performed on animals treated at and up to the dose of 1000 mg/kg of Bergacyn;
- No significant changes in body weight related to the administration of different doses of the test article and vehicle.
- No significant reduction throughout the 90 days treatment in the daily food consumption was found in rats treated with Bergacyn or vehicle.
- No dose-dependent effect on the hematological parameters of rats treated at and up to the dose of 1000 mg/kg was registered.
- No effect on the clinical chemistry parameters of rats treated at and up to the dose of 1000 mg/kg was observed;
- No significant alterations in the organ weights of rats treated at and up to the level of 1000 mg/kg;
- No significant and treatment-related gross and microscopic pathological alterations was found in the tissues of rats treated at and up to the level of 1000 mg/kg.

Based on the findings of this study, the no observable adverse effect level (NOAEL) of Bergamot Polyphenol Fraction and Cynara Cardunculus extract (Bergacyn, Lot No. 1701/16) in Wistar rats, following oral administration for 90 days was found to be more than 1000 mg/kg body weight.

TABLE 4

SURVIVAL DATA

Group & Dose (mg/kg)	Gl & GlR	G2	G3	G4 & G4R
	0	50	200	1000
Week	No. of surviving rats / No. of rats treated			
1	40/40	20/20	20/20	40/40
2	40/40	20/20	20/20	40/40
3	40/40	20/20	20/20	40/40
4	40/40	20/20	20/20	40/40
5	40/40	20/20	20/20	40/40
6	40/40	20/20	20/20	40/40
7	40/40	20/20	20/20	40/40
8	40/40	20/20	20/20	40/40
9	40/40	20/20	20/20	40/40
10	40/40	20/20	20/20	40/40
11	40/40	20/20	20/20	40/40
12	40/40	20/20	20/20	40/40
Reversal Period				
Group & Dose (mg/kg)	GlR		G4R	
	0		1000	
Week 12	20/20		20/20	

TABLE 5

SUMMARY OF CLINICAL SIGNS

Group	G1 & G1R<br>Control	G2<br>Low	G3<br>Intermediate	G4 & G4R<br>High
Dose (mg/kg)	0	50	200	1000
Clinical Finding	Incidence<br>(No. of animals with findings / No. of animals treated)			
No abnormality detected	40/40	20/20	20/20	40/40

TABLE 6

SUMMARY OF FUNCTIONAL OBSERVATIONS

Period: Day 90

Group	G1 & G1R<br>Control	G2<br>Low	G3<br>Intermediate	G4 & G4R<br>High
**Dose (mg/kg)**	**0**	**50**	**200**	**1000**
**No functional abnormalities detected**	40/40	20/20	20/20	40/40

TABLE 7

SUMMARY OF BODY WEIGHT CHANGES (g) – **MALE RATS**

Group and Dose (mg/kg)			Week 0	4	6	8	10	12
			Weight (g)					
G1&G1R	Control 0	Mean	247,19	268,07	289,12	310,01	317,23	326,81
		± SD	12,95	12,14	14,23	12,96	12,25	14,96
G2	Low 50	Mean	245,35	258,06	284,21	306,23	313,34	325,72
		± SD	11,52	11,78	15,39	12,75	14,37	14,94
G3	Intermediate 200	Mean	253,17	272,69	293,11	314,16	321,85	330,38
		± SD	12,14	14,02	11,95	12,14	12,04	11,71
G4&G4R	High 1000	Mean	245,23	268,27	293,14	311,88	320,64	329,01
		± SD	14,39	13,94	12,72	11,91	14,86	10,62

N= 20 for each group

TABLE 7a

SUMMARY OF BODY WEIGHT CHANGES (g) - **FEMALE RATS**

Group and Dose (mg/kg)			Week					
			0	4	6	8	10	12
			Weight (g)					
G1&G1R	Control 0	Mean	249,19	270,07	290,11	320,01	319,25	328,81
		± SD	12,98	12,16	14,36	12,99	12,25	15
G2	Low 50	Mean	251,35	268,06	289,30	306,33	314,34	325,90
		± SD	11,63	11,80	15,78	12,75	15,01	15,01
G3	Intermediate 200	Mean	263,17	276,75	297,10	316,19	321,90	330,38
		± SD	12,99	14,25	12	12,34	12,20	11,71
G4&G4R	High 1000	Mean	255,23	278,32	293,35	313,89	320,67	330,01
		± SD	14,42	13,99	12,78	11,96	14,86	10,62

N= 20 for each group

TABLE 8
FOOD CONSUMPTION **– MALE RATS**

Animal ID	Group and Dose (mg/kg)		Week						Treatment Period Average	Recovery Period Average
			0	4	6	8	10	12		
**G1 & G1R**	**Control**	Average	20,37	19,21	21,10	21,73	26,14	28,14	22,78	27,33
**G2**	**Low 50**	Average	21,21	21	21,52	22,99	23,31	22,47	22,12	22,31
		% of Control	103,21	112,56	95,28	110,56	122,01	109,53	108,85	109,99
**G3**	**Intermediate 200**	Average	21,10	22,15	23,41	24,46	25,62	26,56	23,88	28,66
		% of Control	104,055	107,62	108,46	97,29	119,66	105,26	107,05	128,46
**G4 & G4R**	**High 1000**	Average	21,63	22,47	23,62	24,15	25,62	26,14	23,93	28,72
		% of Control	96,7	103,21	106,47	107,83	107,94	110,77	105,48	126,58

TABLE 8a
FOOD CONSUMPTION – **FEMALE RATS**

Animal ID	Group and Dose (mg/kg)		Week						Treatment Period Average	Recovery Period Average
			0	4	6	8	10	12		
G1 & G1R	Control	Average	20,39	19,26	21,22	21,75	26,19	28,18	22,78	27,33
G2	Low 50	Average	21,23	21,38	21,54	22,99	23,37	22,49	22,12	22,31
		% of Control	103,26	112,60	95,30	110,57	122,07	109,55	108,85	109,99
G3	Intermediate 200	Average	21,19	22,19	23,46	24,47	25,66	26,59	23,88	28,66
		% of Control	104,060	107,75	108,50	97,31	119,69	105,29	107,05	128,46
G4 & G4R	High 1000	Average	21,69	22,74	23,66	24,16	25,68	26,15	23,93	28,72
		% of Control	96,8	103,22	106,48	107,86	107,95	110,78	105,48	126,58

TABLE 9

SUMMARY OF HEMATOLOGICAL DATA – **MALE RATS**

Group and Dose (mg/kg)			Hb (g/dl)	HTC (%)	RBC (x 10^6/ml)	WBC (x 10^3/ml)	Platelets (x 10^3/ml)
GI	Control	Mean	13,93	42,62	6,93	4,89	392,08
		± SD	0,40	2,2	0,408	0,204	162,83
		n	6	6	6	6	6
GIR	Control reversibility	Mean	13,49	40.9	5,76	5,25	383,4
		± SD	0,46	2,85	0.72	0,41	19,26
		n	6	6	6	6	6
G2	Low 50	Mean	13,28	39,06	6,59	4,32	372,6
		± SD	0,40	3,46	0,61	0,51	15,96
		n	6	6	6	6	6
G3	Intermediate 200	Mean	14,42	40,39	7	4,53	361,4
		± SD	0,55	3,57	0,41	0,41	18,74
		n	6	6	6	6	6
G4	High 1000	Mean	13,90	43,4	7,21	5,45	353,90
		± SD	0,52	3,06	0,92	0,72	19,67
		n	6	6	6	6	6
G4R	High 1000 reversibility	Mean	14,62	44,16	5,45	4,22	362,7
		± SD	0,46	3,57	0,82	0,72	16,06
		n	6	6	6	6	6

TABLE 9a

SUMMARY OF HEMATOLOGICAL DATA – **FEMALE RATS**

Group and Dose (mg/kg)			Hb (g/dl)	HTC (%)	RBC (x 10^6/ml)	WBC (x 10^3/ml)	Platelets (x 10^3/ml)
GI	Control	Mean	13,95	42,70	6,94	4,89	392,864
		± SD	0,40	2,20	0,408	0,204	163,155
		n	20	20	20	20	20
GIR	Control reversibility	Mean	13,51	51	5,77	5,26	384,166
		± SD	0,46	2,85	0,72	0,410	19,2985
		n	20	20	20	20	20
G2	Low 50	Mean	13,30	39,13	6,60	4,32	373,345
		± SD	0,40	3,46	0,61	0,511	15,9919
		n	20	20	20	20	20
G3	Intermediate 200	Mean	14,44	40,47	7,014	4,53	362,122
		± SD	0,55	3,57	0,4108	0,410	18,7774
		n	20	20	20	20	20
G4	High 1000	Mean	14,64	43,48	7,22	5,46	354,607
		± SD	0,52	3,066	0,921	0,721	19,7093
		n	20	20	20	20	20
G4R	High 1000 reversibility	Mean	14,62	44,24	5,46	4,22	363,425
		± SD	0,46	3,57	0,82	0,72	16,0921
		n	20	20	20	20	20

TABLE 10

SUMMARY OF CLINICAL CHEMISTRY DATA **– MALE RATS**

Group and dose (mg/kg)			Total protein (g/dl)	Albumin (g/dl)	ALT (IU/L)	Cholesterol (mg/dl)	Triglycerides (mg/dl)	ALP (IU/L)	GGT (IU/L)	Glucose (mg/dl)	Creatinine (mg/dl)	Urea (mg/dl)	Sodium (mmol/L)	Potassium (mmol/L)
GI	Control	Mean	5,25	3,29	35,87	54,09	82,71	94,14	1,36	78,4	0,40	36,5	135,94	4,3
		± SD	0,38	0,22	3,17	9,89	16,17	10,71	0,51	8,62	0,019	2,8	8,68	0,8
		n	6	6	6	6	6	6	6	6	6	6	6	6
GIR	Control reversibility	Mean	5,07	3,22	34,74	47,43	70,85	88,84	1,12	88,2	0,34	41,5	131,8	4,84
		± SD	0,41	0,18	2,87	11,85	18,32	22,74	0,30	6,07	0,03	3,23	9,99	0,59
		n	6	6	6	6	6	6	6	6	6	6	6	6
G2	Low 50	Mean	4,31	3,07	36,08	35,47	60,56	104,75	1,53	83,59	0,50	35,5	141,9	4,35
		± SD	0,35	0,25	13,94	13,32	15,19	22,54	0,61	8,72	0,05	4,44	7,47	0,69
		n	6	6	6	6	6	6	6	6	6	6	6	6
G3	Intermediate 200	Mean	4,89	3,51	32,18	29,59	49,88	96,08	1,74	87,2	0,37	35,4	136,6	5,25
		± SD	0,52	0,31	4,61	11,27	17,83	13,56	0,51	7,15	0,049	3,93	8,38	0,90
		n	6	6	6	6	6	6	6	6	6	6	6	6
G4	High 1000	Mean	4,77	3,18	35,07	32,43	42,72	89,96	1,33	90,06	0,37	39,07	140,89	5,15
		± SD	0,57	0,34	3,99	9,6	18,71	15,6	0,41	9,99	0,049	4,34	8,88	1,31
		n	6	6	6	6	6	6	6	6	6	6	6	6
G4R	High 1000 reversibility	Mean	5,51	3,41	37,31	33,9	51,15	93,73	1,64	82,71	0,39	40,5	142,71	4,24
		± SD	0,30	0,37	4,71	11,07	15,2	18,66	0,30	8,82	0,07	4,44	7,87	0,9
		n	6	6	6	6	6	6	6	6	6	6	6	6

TABLE 10a

SUMMARY OF CLINICAL CHEMISTRY DATA – **FEMALE RATS**

Group and dose (mg/kg)			Total protein (g/dl)	Albumin (g/dl)	ALT (IU/L)	Cholesterol (mg/dl)	Triglycerides (mg/dl)	ALP (IU/L)	GGT (IU/L)	Glucose (mg/dl)	Creatinine (mg/dl)	Urea (mg/dl)	Sodium (mmol/L)	Potassium (mmol/L)
GI	Control	Mean	6,825	4,277	46,631	37,863	57,897	65,898	0,952	54,88	0,34	41,975	156,331	4,73
		± SD	0,494	0,286	4,121	6,923	11,319	7,497	0,357	6,034	0,01615	3,22	9,982	0,88
		n	20	20	20	20	20	20	20	20	20	20	20	20
GIR	Control reversibility	Mean	6,591	4,186	45,162	33,201	49,595	62,188	0,784	61,74	0,289	47,725	151,57	5,324
		± SD	0,533	0,234	3,731	8,295	12,824	15,918	0,21	4,249	0,0255	3,7145	11,4885	0,649
		n	20	20	20	20	20	20	20	20	20	20	20	20
G2	Low 50	Mean	5,603	3,991	46,904	24,829	42,392	73,325	1,071	58,513	0,425	40,825	163,185	4,785
		± SD	0,455	0,325	18,122	9,324	10,633	15,778	0,427	6,104	0,0425	5,106	8,5905	0,759
		n	20	20	20	20	20	20	20	20	20	20	20	20
G3	Intermediate 200	Mean	6,357	4,563	41,834	20,713	34,916	67,256	1,218	61,04	0,3145	40,71	157,09	5,775
		± SD	0,676	0,403	5,993	7,889	12,481	9,492	0,357	5,005	0,04165	4,5195	9,637	0,99
		n	20	20	20	20	20	20	20	20	20	20	20	20
G4	High 1000	Mean	6,201	4,134	45,591	22,701	29,904	62,972	0,931	63,042	0,3145	44,9305	162,0235	5,665
		± SD	0,741	0,442	5,187	6,72	13,097	10,92	0,287	6,993	0,04165	4,991	10,212	1,441
		n	20	20	20	20	20	20	20	20	20	20	20	20
G4R	High 1000 reversibility	Mean	7,163	4,433	48,503	23,73	35,805	65,611	1,148	57,897	0,3315	46,575	164,1165	4,664
		± SD	0,39	0,481	6,123	7,749	1064%	13,062	0,21	6,174	0,0595	5,106	9,0505	0,99
		n	20	20	20	20	20	20	20	20	20	20	20	20

TABLE 11

SUMMARY OF ABSOLUTE ORGANWEIGHTS (g) - **MALE RATS**

Animal ID	Group and Dose (mg/kg)		Organ Weights								
			Adrenals	Spleen	Thymus	Heart	Kidneys	Liver	Testes	Epididymis	Brain
GI	Control	Mean	0,132	0,595	0,295	1,012	1,830	9,423	3,115	1,012	1,732
		+ S.D.	0,02	0,14	0,12	0,09	0,27	0,45	0,21	0,07	0,19
		n	6	6	6	6	6	6	6	6	6
GIR	Control Reversibility	Mean	0,142	0,613	0,273	1,056	2,145	9,332	3,265	1,111	1,815
		+ S.D.	0,06	0,21	0,11	0,10	0,31	0,71	0,31	0,38	0, 10
		n	6	6	6	6	6	6	6	6	6
G2	Low 50	Mean	0,155	0,578	0,268	1,032	2,167	9,515	3,352	1,212	1,822
		+ S.D.	0,06	0,20	0,15	0,09	0,31	0,78	0,36	0,30	0,16
		n	6	6	6	6	6	6	6	6	6
G3	Intermediate 200	Mean	0,172	0,625	0,307	1,022	2,111	9,289	3,418	1,295	1,791
		+ S.D.	0,09	0,18	0,18	0,11	0, 34	0,81	0,50	0,28	0,19
		n	6	6	6	6	6	6	6	6	6
G4	High 1000	Mean	0,121	0,604	0,299	1,071	2,191	9,415	3,611	1,118	1,818
		+ S.D.	0,08	0,13	0,11	0,08	0, 26	0,51	0,41	0,29	0,09
		n	6	6	6	6	6	6	6	6	6
G4R	High 1000 Reversibility	Mean	0,186	0,611	0,282	1,095	2,168	9,321	3,398	1,125	1,841
		+ S.D.	0,08	0, 13	[illegible]	0,16	0,31	0,61	0,40	0,30	0,16
		n	6	6	6	6	6	6	6	6	6

TABLE 11a

SUMMARY OF ABSOLUTE ORGANWEIGHTS (g) – **FEMALE RATS**

Animal ID	Group and Dose (mg/kg)		Organ Weights								
			Adrenals	Spleen	Thymus	Heart	Kidneys	Liver	Testes	Epididymis	Brain
GI	Control	Mean	0,1452	0,6545	0,3245	1,1132	2,013	10,3653	3,4265	1,1132	1,9052
		+ S.D.	0,022	0,154	0,132	0,099	0,297	0,495	0,231	0,077	0,209
		n	20	20	20	20	20	20	20	20	20
GIR	Control Reversibility	Mean	0,1562	0,6743	0,3003	1,1616	2,3595	10,265	3,5915	1,2221	1,9965
		+ S.D.	0,066	0,231	0,121	0,11	0,341	0,781	0,341	0,418	0,1
		n	20	20	20	20	20	20	20	20	20
G2	Low 50	Mean	0,1705	0,6358	0,2948	1,1352	2,3837	10,4665	3,6872	1,3332	2,0042
		+ S.D.	0,066	0,22	0,165	0,099	0,341	0,858	0,396	0,33	0,176
		n	20	20	20	20	20	20	20	20	20
G3	Intermediate 200	Mean	0,1892	0,6875	0,3377	1,1242	2,3221	10,2179	3,7598	1,4245	1,9701
		+ S.D.	0,099	0,198	0,198	0,121	0,34	0,891	0,55	0,308	0,209
		n	20	20	2000%	20	20	20	20	20	20
G4	High 1000	Mean	0,1331	0,6644	0,3289	1,1781	2,4101	10,3565	3,9721	1,2298	1,9998
		+ S.D.	0,088	0,143	0,121	0,088	0,26	0,561	0,451	0,319	0,099
		n	20	20	20	20	20	20	20	20	20
G4R	High 1000 Reversibility	Mean	0,2046	0,6721	0,3102	1,2045	2,3848	10,2531	3,7378	1,2375	2,0251
		+ S.D.	0,088	0,15	0,1474	0,176	0,341	0,671	0,44	0,33	0,176
		n	20	20	20	20	20	20	20	20	20

TABLE 12

SUMMARY OF GROSS PATHOLOGICAL FINDINGS

Group		Gl	G2	G3	G4
Dose (mg *I* kg)		**0**	**50**	**200**	**1000**
Number of animals:		20	20	20	20
No abnormality detected		20	20	20	20
Lungs	- acute congestion		-	-	-
	- infiltration of round cells	-	-	-	-
	- abscess/suppurative<br>- bronchopneumonia	-	-	-	-
	- emphysema	-	-	-	-
Kidneys	- interstitial infiltration of round cell	-	-	-	-
	- tubular cell hyperplasia	-	-	-	-
	- hydronephrosis	-	-	-	-
Spleen	- lymphoid hyperplasia	-	-	-	-
Testes	- atrophic seminiferous tubules	-	-	-	-
Epididymis	- atrophic epididymal ducts	-	-	-	-
Liver	- cyst	-	-	-	-
	- acute congestion	-	-	-	-
	- infiltration of round cells	-	-	-	-
	- necrosis	-	-	-	-
Duodenum	- exfoliation of mucosal epithelium	-	-	-	-
Jejunum	- lymphoid hyperplasia	-	-	-	-
	- exfoliation of mucosal epithelium	-	-	-	-
	- exfoliation of mucosa! epithelium	-	-	-	-
Heart	- fibrinous pericarditis	-	-	-	-

TABLE 13

SUMMARY OF HISTOPATHOLOGICAL FINDINGS

Group	G1	G1R	G2	G3	G4	G4R
**Dose (mg Ikg)**	0	0	50	200	1000	1000
**No abnormality detected**	20/20	20/20	20/20	20/20	20/20	20/20
**Lung –<br>Reddening, abscess**	-	-	-	-	-	-
**Kidneys –<br>slight pallor, hydronephrosis**	-	-	-	-	-	-
**Spleen -<br>slightly enlarged**	-	-	-	-	-	-
**Testes -<br>slightly undersized**	-	-	-	-	-	-
**Liver -<br>Enlarged, sight pllor**	-	-	-	-	-	-
**Heart -<br>Dilatation, fibrosis**	-	-	-	-	-	-

REFERENCES

1. Nogata Y, Sakamoto K, Shiratsuchi H, Ishii T, Yano M, et al. (2006) Flavonoid composition of fruit tissues of citrus species. Biosci Biotechnol Biochem 70: 178-192.
2. Jeong YJ, Choi YJ, Choi JS, Kwon HM, Kang SW, et al. (2007) Attenuation of monocyte adhesion and oxidised LDL uptake in luteolin-treated human endothelial cells exposed to oxidised LDL. Br J Nutr 97: 447-457.
3. Yu J, Wang L, Walzem RL, Miller EG, Pike LM, et al. (2005) Antioxidant activity of citrus limonoids, flavonoids, and coumarins. J Agric Food Chem 53: 2009-2014.
4. Di Donna L, De Luca G, Mazzotti F, Napoli A, Salerno R, et al. (2009) Statin-like principles of bergamot fruit (Citrus bergamia): isolation of 3-hydroxymethylglutaryl flavonoid glycosides. J Nat Prod 72: 1352-1354.
5. Mollace V, Sacco I, Janda E, Malara C, Ventrice D, et al. (2011) Hypolipemic and hypoglycaemic activity of bergamot polyphenols: from animal models to human studies. Fitoterapia 82: 309-316.
6. Gliozzi M, Walker R, Muscoli S, Vitale C, Gratteri S, et al. (2013) Bergamot polyphenolic fraction enhances rosuvastatin-induced effect on LDL-cholesterol, LOX-1 expression and protein kinase B phosphorylation in patients with hyperlipidemia. Int J Cardiol 170: 140-145.
7. Gliozzi M, Carresi C, Musolino V, Palma E, Muscoli C et al. (2014) The effect of bergamot-derived polyphenolic fraction on LDL small dense particles and non alcoholic fatty liver disease in patients with MS. Advances in Biological Chemistry.
8. Leighton F, Miranda-Rottmann S, Urquiaga I (2006) A central role of eNOS in the protective effect of wine against metabolic syndrome. Cell Biochem Funct 24: 291-298.
9. Mollace V, Ragusa S, Sacco I, Muscoli C, Sculco F, et al. (2008) The protective effect of bergamot oil extract on lecitine-like oxyLDL receptor-1 expression in balloon injury- related neointima formation. J Cardiovasc Pharmacol Ther 13: 120-129.
