# Supplementary material for: Randomized Clinical Trial: Bergamot Citrus and Wild Cardoon Reduce Liver Steatosis and Body Weight in Non-diabetic Individuals Aged Over 50 Years
Source: Front Endocrinol (Lausanne). 2020 Aug 11;11:494. doi: 10.3389/fendo.2020.00494 (PMC7431622; doi:10.3389/fendo.2020.00494)
Supplement: Supplementary file 1 [file Data_Sheet_1.zip › Supl. mater. 3- pharmacokinetics_ previous study.pdf]

Hypoglycemic and Hypolipemic effects of a new lecithin formulation of Bergamot Polyphenolic Fraction: a double blind, randomized, placebo-controlled study

Vincenzo Mollace[1*], Sara Paone[1], Francesca Casale[1], Carla Calandruccio[1], Micaela Gliozzi[1], Vincenzo Musolino[1], Cristina Carresi[1], Jessica Maiuolo[1], Miriam Scicchitano[1], Saverio Nucera[1], A. Riva[2], P. Allegrini[2], M. Ronchi[2], G. Petrangolini[2] and E. Bombardelli[2]

[1]*Institute of Research for Food Safety & Health (IRC-FSH), Department of Health Sciences, University "Magna Graecia" of Catanzaro, Catanzaro, Italy.*

[2]*Research and Development Unit, Indena S.p.A., Milan, Italy*

[*]**Corresponding author:** Vincenzo Mollace, Viale Europa, Loc. Germaneto, 88100 Catanzaro, Italy, E-mail: mollace@unicz.it

Abstract

The occurrence of hyperlipemia represents an independent risk factor for developing cardiovascular disease states in patients suffering from type 2 diabetes mellitus (DM). Moreover, the pharmacological treatment of dyslipemia in patients undergoing type 2 DM (e.g. by means of statins), is accompanied by relevant side effects and oral supplementation with natural antioxidants, such as Citrus polyphenols, has recently been suggested to improve cardioprotection in such patients. However, due to the poor gastrointestinal absorption of polyphenols, novel formulations have recently been developed in order to obtain a better profile of citrus species extract rich in polyphenols.

Here we studied the effect of standard bergamot polyphenolic fraction (BPF®) as well as of its phytosomal formulation (BPF Phyto), in patients with type 2 DM and hyperlipemia.

A randomized, double blind, placebo-controlled study was carried out in 60 patients suffering from type 2 DM and mixed hyperlipemia. Patients were divided into three groups: one receiving placebo, the second receiving standard BPF and the third BPF Phyto. In the groups receiving BPF and BPF Phyto, a significant reduction of fasting plasma glucose, serum LDL cholesterol and triglycerides alongside with an increase of HDL cholesterol was found. This effect was accompanied by a significant reduction of small dense atherogenic LDL particles, as detected via proton NMR Spectroscopy, thus confirming the beneficial effect of bergamot extract both when using standard formulation as well as BPF Phyto.

No differences were seen in the therapeutic response among groups receiving BPF and BPF Phyto, thus suggesting a substantial bioequivalence in their hypoglycemic and hypolipemic profile. However, when comparing the pharmacokinetic profile of naringin (the major component of BPF) and its metabolites, in patients treated with BPF Phyto, an at least 2,5 fold increase in its absorption was found, confirming in human studies the better profile of BPF Phyto compared to standard BPF.

These data suggest that better absorption and tissue distribution of BPF Phyto formulation represents a novel and potentially useful approach in supplementation treatments of cardiometabolic disorders.

Keywords: type 2 diabetes mellitus, hyperlipemia, bergamot polyphenolic fraction, BPF Phytosome, bioavailability, flavonoids

Introduction

Increasing experimental and epidemiological evidence suggests that dietary polyphenols, in particular flavonoids, may play an important role in counteracting pathophysiological mechanisms leading to the development of hyperlipemia either combined or not with Type 2 Diabetes mellitus (DM)[1]. These beneficial effects are generally attributed to their anti-oxidant properties, as well as to more specific mechanisms such as the modulation of metabolic enzymes, nuclear receptors, gene expression and multiple signaling pathways [2-3].

Bergamot (*Citrus Bergamia Risso et Poiteau*) is an endemic plant of the Calabrian region in the Southern Italy with a unique profile of flavonoid and flavonoid glycosides detectable in its juice and albedo. Bergamot differs from other Citrus fruits because of the composition and the particularly high content of its flavonoids [4,5] able to counteract the detrimental effect of hyperlipidemia through a multi-action mechanism. In particular, naringin has already been reported to be active in animal models of atherosclerosis [6], while neoeriocitrin and rutin have been shown to inhibit LDL oxidation [7]. Moreover, two glycosilated flavonoids exclusive of bergamot-derivatives identified as melitidine and bruteridine (neohesperidosides of hesperetin and naringenin, respectively), were found possessing a structural similarity to the physiological substrate of HMG-CoA reductase, thereby exhibiting statin-like proprieties [8].

Recently, the therapeutic potential of bergamot derivatives has also been investigated in human studies [9-11]. In particular, experimental and epidemiological studies revealed that bergamot polyphenolic fraction (BPF) improves serum lipemic profile and normalizes blood glucose in patients suffering from metabolic syndrome (MS) [12-15]. The hypolipemic effect might be due to its ability to reduce hepatic TG accumulation and to down-regulate the activity of TG synthetic enzyme phosphatidate phosphohydrolase [16]. Moreover, in vitro studies show that naringenin and hesperetin reduced activities of acyl CoA:cholesterol acyltransferases (ACAT), thus decreasing the availability of lipids needed to assembly apoB-containing lipoproteins [17]. In addition, melitidine and brutieridine, in concert with naringin and other flavonone glycosides, might be responsible for the notable potency of BPF in reducing cholesterol levels, being 3-hydroxy-3-methylglutaryl derivatives of hesperetin and naringenin, respectively. Finally, BPF has been shown to inhibit pancreatic cholesterol

ester hydrolase (pCEH), thereby reducing the rate of dietary cholesterol absorption at the intestinal level [18].

As far as the vasoprotective effect of BPF is concerned, its antioxidant effect seems to play a major role. In particular, evidence shows that eNOS knockout mice exhibit a cluster of cardiovascular risk factors comparable to those of MS suggesting that a decreased eNOS activity might cause MS in human, inducing an impaired NO-dependent vasodilation [11, 19,20]. In particular, oxidative stress and inflammatory processes observed in MS can justify endothelial dysfunction induced by eNOS-down-regulation. In this context, Citrus flavonoids, by increasing superoxide dismutase and catalase activities [21], may attenuate overproduction of oxygen reactive species in the vascular wall thereby preventing endothelial dysfunction, as also detected in patients under BPF treatment.

An additional beneficial effect of BPF might be related to its hypoglycemic activity; indeed, it has been demonstrated that naringenin, similarly to other polyphenols, significantly increased AMP kinase (AMPK) activity and glucose uptake in muscle cells and liver [22-24].

Overall, these discoveries suggest that the supplementation of an ordinary diet with BPF is an alternative phytotherapeutic approach to better control prediabetic states in patients with hypelipemia, because it induces a normalization of lipid profile, an amelioration of NO-dependent vasoreactivity and a reduction of blood glucose levels [8].

Besides these evidence, the rate of BPF absorption remains low and better formulation is required to increase BPF bioavailability in order to improve its efficacy and safety profile. Recently, an highly standardized BPF extract was formulated with the innovative food grade delivery system Phytosome® (BPF Phytosome®) [25]. In particular, this innovative strategy involves the use of lipid-compatible molecular complexes in which water-soluble phytoconstituents can be formulated into a lipid-compatible molecules known as PHYTOSOME® [26,27]. This new formulation of BPF has been proven to enhance the rate of polyphenol absorption in rats [25], and this suggests that BPF phytosomal (BPF Phyto) formulation may be relevant in reducing serum glucose and lipids in patients.

Here we investigated on the effect of BPF phyto in patients with hyperlipemia and type 2 DM by means of a double blind, randomized, placebo-controlled study. The effect of BPF Phyto was compared with standard BPF formulation.

Materials and Methods

Preparation of BPF® and BPF Phytosome®

C. bergamia Risso & Poiteau fruits were collected from plants located in a range of 90 Km from Bianco to Reggio Calabria, Italy.

Bergamot juice was obtained from peeled-off fruits by squeezing. The juice was oil fraction-depleted by stripping, clarified by ultra-filtration and loaded on to a suitable polystyrene resin column able to absorb polyphenol compounds of molecular weight between 300 to 600 Da (Mitsubishi). Polyphenol fractions were eluted by a 1mM KOH solution. The basic eluate was incubated at a rocking platform to reduce the furocumarin content. The shaking time was adjusted proportionally to the amount of furocumarin contaminants. Next, the phytocomplex derived from the process performed to remove furocumarins was neutralized by filtration on cationic resin at acidic pH. Finally it was vacuum dried and minced to the desired particle size to obtain BPF powder. BPF powder was analysed for flavonoid, furocumarin and other polyphenol content which was standardized at 40%. In addition, all toxicological analyses were performed, including heavy metal, pesticide, phthalate and sinephrine content which revealed the absence of known toxic compounds at significant levels (data not shown). Standard microbiological test showed the final BPF was free of mycotoxins and contaminating bacteria. The main flavonoids identified in 38% BPF were neoeriocitrin (370 ppm), naringin (520 ppm), and neohesperidin (310 ppm).

Dietary phospholipids (sunflower lecithin) were formulated with 40% in weight standardized BPF extract (provided by HEAD Research group, Bianco, Italy) in order to enhance oral bioavailability of BPF main flavonoids.

Tablets containing standard formulation with 650 mg of the BPF powder or 500 mg of BPF Phytosome (containing 200 mg of BPF Extract) were provided by Indena SpA (Milan, Italy). All procedures have been performed according to Food Supplement European Regulation.

Tablets with no active ingredients were used as placebo.

Study Design

A double blind, randomized, placebo-controlled study was carried out in 60 patients suffering from mixed hyperlipemia (LDL cholesterol > 120 mg/dl and triglycerides > 175 mg/dl) and

type 2 DM (serum glucose > 110 mg/dl) enrolled at the Clinical Trial Center of the International Research Center for Food Safety & Health (IRC-FSH) of University of Catanzaro "Magna Graecia". After randomization, patients were taking placebo (Group 1), BPF 650 mg (Group 2) or BPF Phyto 500 mg (Group 3) twice a day before meals for 30 consecutive days.

All participants provided written informed consent prior to participation. The study was approved by the local Medical Ethics Committee and conducted according to the principles of Good Clinical Practice (GMP), EU directives 2001/20/EC, 2005/28/EC and the Declaration of Helsinki (1964).

Data Collection and Measurements

At baseline, all participants were asked to come in the morning after fasting for >10 h. Fasting blood samples were collected for measuring conventional risk factors of cardiometabolic diseases, including serum lipids, glucose, transaminases and inflammatory markers. Face-to-face interviews and physical examinations were performed by well-trained nurses or physicians. Demographic and lifestyle information was collected by a standardized questionnaire (see table 1). Excessive drinkers with alcohol consumption of ≥20 g per day in males or ≥10 g per day in females were not included in this study.

Laboratory Measurements

Plasma samples were collected in EDTA-containing vials after a 12 h overnight fast on day 0 and after 30 days of treatment with placebo, BPF Phyto and standard BPF formulation and stored at -20°C for both hematological biomarkers and for pharmacokinetic studies.

Total cholesterol (in mg/dL), high density lipoprotein cholesterol (HDL-C), low density lipoprotein cholesterol (LDL-C), triglycerides (TG) and fasting plasma glucose, were evaluated at baseline and after 30 days of treatment with placebo, standard BPF and BPF Phyto. Under the same treatment schedule, lipoprotein particles were detected by means of proton NMR spectroscopy technique which simultaneously measures the particle concentrations of lipoprotein subclasses of different sizes. Each of the lipoprotein subclasses emits a distinctive NMR signal, the amplitude of which is directly proportional to the number of subclass particles emitting the signal. Importantly, variation in lipoprotein particle lipid composition does not alter the relationship between the NMR signal and the particle size. The NMR also

provides calculated values for mean very-low-density lipoprotein (VLDL), LDL, Intermediate-Density Lipoprotein (IDL) and HDL particle sizes plus estimates of total and VLDL, TG and HDL cholesterol. NMR-based estimates of TG and HDL cholesterol were calculated using conversion factors that assume normal lipid content of the various subclasses.

Pharmacokinetic studies

Reagents, drugs and supplements

Naringin and naringenin (Purity (HPLC) ≥99%) were purchased from Extrasynthese (Genay, France). Naringenin glucuronide was kindly provided by Prof. Procopio, C<, Italy. HPLC-grade methanol, acetonitrile and acetic acid were obtained from Merck (Darmstadt, Germany). All other chemicals and solvents used were of analytical grade. Bergamot Polyphenolic Fraction (BPF), patented by H&AD Srl (Trademark and patent No. 0001380456 by Herbal and Antioxidant Derivatives S.R.L.). The PHYTOSOME®, a patented technology developed by Indena S.p.A. (Milan, Italy), C18 Oasis®HLB cartridges purchased by Waters. BPF Phytosome (Indena S.p.A) consists of dietary phospholipids (sunflower lecithin) formulated with a standardized BPF extract (H&AD Srl).

Working solutions and Calibration standards,

All chemicals were used as commercially available. The standardization of the method was developed using 99% pure standards of naringin and its metabolites naringenin and naringenin glucuronide mixed in a hydroalcoholic solution of MeOH/H2O 80:20 (v/v) to give the following concentrations: 1, 10, 50, 100, 500 ppm.

Sample handling

The 4-ml aliquot of all blood samples was then divided into 2 aliquots (2 ml each) in order to collect plasma and serum. For plasma collection, EDTA (200 microliters) was added to one aliquot. For serum separation, the other aliquot was left in rest at room temperature (30 minutes).

Then vials were centrifuged at 10,000 rmp for 10 min at 4 °C. From vials containing EDTA at most 200-600 microliters of plasma was decanted, aliquots were flash-frozen in liquid nitrogen and stored at -80° C for LC-UV analysis. From vials containing serum, about 1 ml was stored at 2-8°C for later XL640 analysis.

After the selection of the more accurate analytical method, naringin, naringenin and naringenin glucuronide were determined by UHPLC-UV post- Solid Phase Extraction (SPE).

Chromatographic method and pharmacokinetic parameters

The chromatography separation was performed using a Dionex UHPLC UltiMate® 3000 Solvent Rack (Thermo Scientific) equipped with a reverse-phase C18 column Hypersil GOLD-(Dim.(mm) 250 x 4.6; Particle Sz. (µ) 5) operating at flow rate1.0 mL/min. The volume injected 20 µL. Pump model: LPG-3400SD 0-620 bar (9000 psi). Detection was performed by a Photometer MWD-3000 monitoring the absorbance signals between 210-350 nm and the data elaboration was carried out selecting the wavelength of maximum absorption of the analytes: 280 nm. The mobile phase was A) trifluoracetic acid 0.1% (v/v) in aqueous solution and B) methanol; the gradient used was as follows: zero time condition was 5% A and it was increased to 95% A in 30 minutes.

Pharmacokinetic parameters were determined by means of a non-compartmental analysis using the WinNonlin Professional software version 3.3 (Phar-sight Corporation, USA). The linear trapezoidal method was used to calculate the area under the plasma concentration curve (AUC $0-24$) from time 0 until the detectable concentration at 24h. The maximum plasma concentration (C_{max}) and the time needed to reach C_{max} were determined by visual inspection of the experimental data.

Statistical Analysis

For continuous variables, differences between the BPF and baseline levels were assessed using Student's t test for independent samples. Data analyses were conducted using SPSS software (version 18.0).

Results

Effect of placebo, BPF and BPF Phyto in hyperlipemic and hyperglycemic patients

Demographics and glyco-lipemic serum profile in patients with type 2 DM at baseline as well as following 30 consecutive days of treatment with placebo, BPF and BPF Phyto are shown in Table 1.

Basal levels showed a mixed hyperlipemia (elevated total cholesterol plus hypertriglyceridemia) associated with moderate glucose elevation. High LDL cholesterol was associated with reduced HDL cholesterol and elevated fasting serum glucose, suggesting the occurrence of MS.

In patients treated with BPF (650 mg given orally twice a day before meals) or BPF Phyto (500 mg given orally twice a day before meals) for 30 consecutive days, a significant reduction of serum total cholesterol, LDL-C and triglycerides was found compared to placebo group (Figure 1). This effect was accompanied by significant reduction of serum glucose. No differences were found between BPF or BPF Phyto groups of patients suggesting a substantial bioequivalence on their hypolipemic and hypoglycemic effect .

Moreover, a substantial rearrangement of lipoprotein particles was found in patients treated with BPF and BPF Phyto compared to placebo group (Figure 2a-d). Indeed, as measured using NMR, data detected in patients following 30 days of BPF or BPF Phyto treatment showed relevant changes in mean particle diameters for VLDL, LDL, and HDL compared to the Placebo group.

In particular, BPF and BPF Phyto were found able to decrease the mean concentration of IDL particles, to increase large LDL and to decrease small LDL. Moreover, 30 day treatment with BPF as well with BPF Phyto leads to increase of total HDL particles, mainly due to the increase of large HDL. Also the re-arrangement of lipoprotein profile occurred with no significant differences between standard formulation of BPF and BPF Phyto group.

Neither relevant side effects nor changes in red blood cells, white blood cell counts as well as transaminases were found throughout the study in all groups of patients receiving placebo, BPF and BPF Phyto, respectively (not shown).

Pharmacokinetics of naringin and its metabolites after oral intake of BPF, BPF Phyto and placebo

Naringin in its native form and two flavanone metabolites were identified in plasma after the oral intake of BPF and BPF Phyto. In particular, naringin concentrations peaked at time 60-80 min and declined significantly after 2-4h being not detectable at day 2 after the administration of BPF and BPF Phyto. Citrus flavanones and their metabolites were not present in plasma of patients at time 0, prior supplementation with BPF or BPF Phyto, or in the control subjects that had been given placebo.

Figure 3 represents the plasma concentration curves for naringin after supplementation with BPF or BPF Phyto. Values were expressed as means and standard deviations.

The following pharmacokinetic parameters (AUC_{0-24h}, C_{max} and Time to C_{max}) corresponding to naringin and its metabolites in plasma of patients treated with BPF and BPF Phyto are summarised in Table 2. Data, collected after single administration of BPF or BPF Phyto revealed that there were no significant statistical differences between the pharmacokinetic parameters corresponding to naringin, naringenin and naringenin glucuronide among patients of both groups, thus demonstrating that both formulations are equivalent in terms of plasma concentrations of major flavonone naringin and its metabolites.

Discussion

The present data confirmed previous results showing that BPF, the extract of bergamot juice rich in polyphenols, reduces both cholesterol, triglycerides and glucose in patients suffering from Type 2 DM [22-23]. This effect is accompanied by reduction of LDL-C and elevation of HDL-C, thus suggesting a beneficial effect in the lipemic profile of patients undergoing MS. The added value and novelty of data reported in this study using BPF in such a subgroup of patients with elevated cardiometabolic risk, is also displayed by prominent re-arrangement of lipoprotein particle profile found following 30 day BPF treatment. Indeed, BPF reduced LDL small-size, atherogenic particles and enhanced large-size anti-atherogenic HDL lipoprotein particles. This effect highlights that BPF leads to an attenuation of atherogenic risk in patients with Type 2 DM.

Our data also show that using BPF in Phytosome leads to bioequivalent response in patients, an effect associated to better polyphenolic oral absorption. In fact, BPF Phyto, compared to

standard BPF formulation, leads to near 2,5 fold increase in the rate of naringin serum concentration, an effect confirmed by measurement of its metabolite naringenin and naringenin glucuronide. This effect was accompanied by substantial comparable response of BPF Phyto on serum lipemic and glycemic responses of patients with type 2 DM, including the significant re-arrangement of lipoproteins which are addressed to a lesser atherogenic profile compared to placebo-treated group. As for BPF standard formulation, the effect of BPF Phyto occurred in the absence of significant side effects, thereby confirming the safety profile of both formulations.

Our experiments confirm previous in vitro ed in vivo studies carried out in rats, showing that using BPF in Phytosome significantly enhances polyphenol absorption when given orally (25,26) and shed new light into studies concerning oral supplementation with citrus polyphenols.

In recent years, a greater understanding of flavonoid absorption and metabolism has been achieved. Flavonoid glycosides are thought to reach the small intestine intact, and it is believed that they may require deglycosidation for absorption across the intestine (28, 29). The presence of naringin in the plasma of patients treated with standard BPF or BPF Phyto demonstrates that the deglycosidation of naringin is not always necessary for its absorption. Previous studies (30) have administered naringin as a pure compound, whereas in the present study citrus flavanones were administered in the form of a bergamot extract (as it occurs in nature) either in standard and in phytosomal formulation. In both cases, naringin was detected into a range of effective concentrations, being BPF Phyto much more suitable for oral administration due to the better absorption displayed in patients.

Furthermore, our data show that three different flavanone forms were found in plasma of both BPF and BPF phyto-treated patients, thus demonstrating naringin absorption after an oral intake of bergamot extract: naringin in its native form, naringenin and naringenin glucuronide. These results confirm the bioavailability of BPF flavanones and their metabolites in diabetic patients after the oral administration of BPF in standard formulation as well as in Phytosome settings. The aglycone naringenin showed the highest rate of absorption but the lowest extended exposure and lowest retention time in the body. Both naringin and naringenin glucuronide showed high extended exposure values, whereas naringenin glucuronide presented the highest values for retention time, remaining in the body for approximately 8 h.

All these effects are highlighted when phytosomal formulation of BPF is used.

Conclusion

In conclusion, our data show that BPF formulated with Phytosome displays a better absorption and pharmacokinetic profile compared to standard formulation. Furthermore, BPH Phyto showed a substantial bioequivalence compared to standard BPF in terms of efficacy (hypolipemic and hypoglycemic effect) in type 2 diabetes and an identical safety profile.

Conflict of interest: The other authors declare no conflict of interest.

AR, PA, MR, GP are employees of Indena S.p.A., Milan, Italy.

EB is consultant for Indena S.p.A., Milan, Italy.

Acknowledgements

This paper has been supported by PON03PE_00078_1 and PON03PE_00078_2.

References

[1]Cherniack EP. Polyphenols: planting the seeds of treatment for the metabolic syndrome. Nutrition 2011;27:617-623.

[2]Fraga CG, Galleano M, Verstraeten SV, Oteiza PI Basic biochemical mechanisms behind the health benefits of polyphenols. Mol Aspects Med 2010;31:435-445.

[3]Seeram NP. Berry fruits: compositional elements, biochemical activities, and the impact of their intake on human health, performance, and disease. J Agric Food Chem 2008;56:627-629.

[4]Dugo P, Presti ML, Ohman M, Fazio A, Dugo G, Mondello L. Determination of flavonoids in citrus juices by micro-HPLC-ESI/MS. J Sep Sci 2005;28:1149-1156.

[5]Nogata Y, Sakamoto K, Shiratsuchi H, Ishii T, Yano M, Ohta H. Flavonoid composition of fruit tissues of citrus species. Biosci Biotechnol Biochem 2006;70:178-192.

[6]Jeong YJ, Choi YJ, Choi JS, Kwon HM, Kang SW, Bae JY, et al. Attenuation of monocyte adhesion and oxidised LDL uptake in luteolin-treated human endothelial cells exposed to oxidised LDL. Br J Nutr 2007;97:447-457.

[7]Yu J, Wang L, Walzem RL, Miller EG, Pike LM, Patil BS. Antioxidant activity of citrus limonoids, flavonoids, and coumarins. J Agric Food Chem 2005;53:2009-2014.

[8]Di Donna L, De Luca G, Mazzotti F, Napoli A, Salerno R, Taverna D, et al. Statin-like principles of bergamot fruit (Citrus bergamia): isolation of 3-hydroxymethylglutaryl flavonoid glycosides. J Nat Prod 2009;72:1352-1354.

[9] Mollace V, Sacco I, Janda E, Malara C, Ventrice D, Colica C, et al. Hypolipemic and

hypoglycaemic activity of bergamot polyphenols: from animal models to human studies. Fitoterapia 2011;82:309-316.

[10] Gliozzi M, Walker R, Muscoli S, Vitale C, Gratteri S, Carresi C, et al. Bergamot polyphenolic fraction enhances rosuvastatin-induced effect on LDL-cholesterol, LOX-1 expression and protein kinase B phosphorylation in patients with hyperlipidemia. Int J Cardiol 2013;170:140-145.

[11] Gliozzi M, Carresi C, Musolino V, Palma E, Muscoli C, Gratteri S, et al. The effect of bergamot-derived polyphenolic fraction on LDL small dense particles and non alcoholic fatty liver disease in patients with MS. Advances in Biological Chemistry 2014;4:129-137 doi: 10.4236/abc.2014.42017.

[12] Leighton F, Miranda-Rottmann S, Urquiaga I. A central role of eNOS in the protective effect of wine against metabolic syndrome. Cell Biochem Funct 2006;24:291-298.

[13] Mollace V, Ragusa S, Sacco I, Muscoli C, Sculco F, Visalli V, et al. The protective effect of bergamot oil extract on lecitine-like oxyLDL receptor-1 expression in balloon injury-related neointima formation. J Cardiovasc Pharmacol Ther 2008;13:120-129.

[14] Nogata Y, Sakamoto K, Shiratsuchi H, Ishii T, Yano M, Ohta H. Flavonoid composition of fruit tissues of citrus species. Biosci Biotechnol Biochem 2006;70:178-192.

[15] Choe SC, Kim HS, Jeong TS, Bok SH, Park YB. Naringin has an antiatherogenic effect with the inhibition of intercellular adhesion molecule-1 in hypercholesterolemic rabbits. J Cardiovasc Pharmacol 2001;38:947-955.

[16] Vinson JA, Liang X, Proch J, Hontz BA, Dancel J, Sandone N. Polyphenol antioxidants in citrus juices: in vitro and in vivo studies relevant to heart disease. Adv Exp Med Biol 2002;505:113-122.

[17] Cha JY, Cho YS, Kim I, Anno T, Rahman SM, Yanagita T. Effect of hesperetin, a citrus flavonoid, on the liver triacylglycerol content and phosphatidate phosphohydrolase activity in orotic acid-fed rats. Plant Foods Hum Nutr 2001;56:349-358.

[18] Musolino et al

[19] Kim HJ, Oh GT, Park YB, Lee MK, Seo HJ, Choi MS. Naringin alters the cholesterol biosynthesis and antioxidant enzyme activities in LDL receptor- knockout mice under cholesterol fed condition. Life Sci 2004;74:1621-1634.

[20] Mollace V, Muscoli C, Masini E, Cuzzocrea S, Salvemini D. Modulation of prostaglandin biosynthesis by nitric oxide and nitric oxide donors. Pharmacol Rev 2005;57:217-252.

[21] Salvemini D, Kim SF, Mollace V. Reciprocal regulation of the nitric oxide and cyclooxygenase pathway in pathophysiology: relevance and clinical implications. Am J Physiol Regul Integr Comp Physiol 2013;304:R473-487.

[22] Jeon SM, Bok SH, Jang MK, Lee MK, Nam KT, Park YB, et al. Antioxidative activity of naringin and lovastatin in high cholesterol-fed rabbits. Life Sci 2001;69:2855-2866.

[23] Hwang JT, Kwon DY, Yoon SH. AMP-activated protein kinase: a potential target for the diseases prevention by natural occurring polyphenols. N Biotechnol 2009;26:17-22.

[24] Zygmunt K, Faubert B, MacNeil J, Tsiani E. Naringenin, a citrus flavonoid, increases muscle cell glucose uptake via AMPK. Biochem Biophys Res Commun 2010;398:178-183.

[25] Casale F, Calandruccio C, Musolino V, Nucera S, Gliozzi M, Carresi C et al., Studies on the Increased Bioavailability of a New Lecithin Formulation of Bergamot Flavonoids: pre-clinical studies. End. Metab, Immune Dis. , 2017; in press

[26] Bombardelli E, Curri SB, Della Loggia R, Del Negro P, Tubaro A, Gariboldi P.

Complexes between phospholipids and vegetable derivatives of biological interest, Fitoterapia 1989; 60, 1-9.
[27] Jain N, Gupta BP, Takur R, Kain R, Banweer J, Jain DK, Jain S. Phytosome. A novel drug delivery system for herbal medicine. Int. J. Pharm. Sci. Drug Discovery 2010; 2 (4), 224-228.
[28] Manach C, Scalbert A, Morand C, Remesy C & Jimenez L (2004) Polyphenols: food sources and bioavailability. Am J Clin Nutr 79, 727 –7.
[29] Scalbert A & Williamson G (2000) Dietary intake and bioavailability of polyphenols. J Nutr 130, 2073S– 2085S
[30] Fang T, Wang Y, Ma Y, Su W, Bai Y & Zhao P A rapid LC/ MS/MS quantitation assay for naringin and its two metabolites in rat's plasma. J Pharm Biomed Anal 2006; 40, 454 – 459.
