# Supplementary material for: Randomized Clinical Trial: Bergamot Citrus and Wild Cardoon Reduce Liver Steatosis and Body Weight in Non-diabetic Individuals Aged Over 50 Years
Source: Front Endocrinol (Lausanne). 2020 Aug 11;11:494. doi: 10.3389/fendo.2020.00494 (PMC7431622; doi:10.3389/fendo.2020.00494)
Supplement: Supplementary file 1 [file Data_Sheet_1.zip › Supplemental Figure1.docx]

**Supplemental Material**

**Figure 1**

**BPF: Bergamot Polyphenolic Fraction**

**Specification sheet**

| **ORGANOLEPTIC** | | |
| --- | --- | --- |
| Colour | **Yellow Powder** | **visual (CQ-MO-148)** |
| Odour | **Aromatic** | **visual (CQ-MO-148)** |
| Flavour | **Characteristic of bergamot** | **sensory (CQ-MO-148)** |

| **CHEMICAL CHARACTERISTICS** | | |
| --- | --- | --- |
| pH | **3.0 – 4.0** | **IM (0.5% in water) at 25°C** |
| Average Mesh Size | **Pass 70 mesh** | **Sieve: (CQ-MO-023)** |
| Bulk Density | **30-70g/100ml** | **PT CHIM 65 rev 0 2011** |
| Moisture Content | **< 10.0%** | **ISTISAN 96/34, pag 7** |
| Organic Solvent Residue | **None** | **GC: (CQ-MO-168)** |
| Soluble in 40°C H_2_O | **Good** | **visual: (CQ-MO-148)** |
| Soluble in 50% H_2_O + EtOH | **Good** | **visual: (CQ-MO-148)** |
| Active Ingredient Strength | **HPLC** |  |
| Pesticides Residue | **Negative** | **PT CHIM 69rev 02 011** |
| **ACTIVE INGREDIENTS** | **UNIT** | **RANGE** |
| Polyphenols (Neoeriocitrin, Naringin, Neohesperidin, Melitidin, Bruteridin) | % | **38%** |

| **DESCRIPTION** | **SPECIFICATIONS** | **METHODS** |
| --- | --- | --- |
| Botanical Source | **Citrus Bergamia Risso et Poit.** |  |
| Family | **Rutaceae** |  |
| Synonyms | **Citrus aurantium var. bergamia** |  |
| Country of Origin | **Calabria, Italy** |  |
| Part Used | **Fruit** |  |
| Shelf Life | **3 years, if correctly stored** |  |

| **HEAVY METALS** | | |
| --- | --- | --- |
| Arsenic | ppm | **<2.0** |
| Lead | ppm | **<2.0** |
| Heavy Metals (tot. amount) | ppm | **<20.0** |

| **MICROBIOLOGICAL EVALUATION** |  |  |
| --- | --- | --- |
| Aerobic Plate Count | **<1,000 CFU/g** | **ISO 4833-1:2013** |
| Yeast and Mold Count | **<100 CFU/g** | **ISO 21527-1:2008** |
| E. Coli | **Negative** | **ISO 16694-2:2001** |
| Coliform | **Negative** | **ISO 4832:2006** |
| Salmonella | **Negative** | **UNI EN ISO 6579:2000** |
| Staphylococcus Aureus | **Negative** | **UNI EN ISO 6888-2:2004** |
| Streptococci | **Negative** | **PT BAT26 rev0 02012** |
| **PRODUCT TREATMENT** | | |
| Extraction solvents | **Water+KOH** |  |
| Drying Method | **Sprydry** |  |

**DI.**

**Figure 2**

**CyC: Cynara Cardunculus Extract**

**Specification sheet**


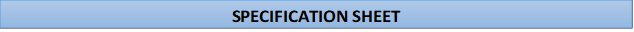


| **DESCRIPTION** | **SPE** | **CIFICATIONS** | **METHODS** |
| --- | --- | --- | --- |
| ID Code | **CRCESTPC** | |  |
| Trade Name | **CYC ®** | |  |
| Botanical Source | **Cynara Cardunculus** | |  |
| Family | **Asteraceae** | |  |
| Synonyms | **Cynara Cardunculus** | |  |
| Country of Origin | **Italy** | |  |
| Part Used | **Leaf** | |  |
| **ORGANOLEPTIC** | | | |
| Colour | **Gold Green Powder** | | **visual (CQ-MO-148 )** |
| Odour | **Aromatic** | | **visual (CQ-MO-148 )** |
| Flavour | **Characteristic of Cynara leaf** | | **sensory (CQ-MO-148)** |
| **CHEMICAL CHARACTERISTICS** | | | |
| pH | **3.0 – 5.0** | | **IM (0.5% in water) at 25°C** |
| Average Mesh Size | **Pass 70 mesh** | | **Sieve: (CQ-MO-02 3)** |
| Bulk Density | **40-70 g/100mL** | | **PT CHIM 65 rev 0 2011** |
| Moisture Content | **< 8.0%** | | **ISTISAN 96/34, pa g 7** |
| OrganicSolvent Residue | **None** | | **GC: (CQ-MO-168)** |
| Soluble in 40°C H 2O | **Good** | | **visual: (CQ-MO-148)** |
| Soluble in 50% H2O + EtOH | **Good** | | **visual: (CQ-MO-148)** |
| Active IngredientStrength | **HPLC** | |  |
| Pesticides Residue | **Negative** | | **PT CHIM 69rev 02 011** |
| **COMPONENT** | **UNI** | **T** | **RANGE** |
| Cynaropicrin | **%** | | **10%** |
| Total flavonoids (as luteolin-7-O- glucoside) | **%** | | **15%** |
| Caffeoylquinic acid (as Chlorogenic acid) | **%** | | **6%** |
| **HEAVY METALS** | | | |
| Arsenic | **mg/kg** | | **<1.0** |
| Lead | **mg/kg** | | **<0.5** |
| Cadmium | **mg/kg** | | **<0.5** |
| Mercury | **mg/kg** | | **<0.3** |
| **MICROBIOLOGICAL EVALUATION** | | | |
| Aerobic Plate Count | **<1,000 CFU/g** | | **ISO 4833-1:2013** |
| Yeast and Mold Count | **<100 CFU/g** | | **ISO 21527-1:2008** |
| E. Coli | **Negative** | | **ISO 16694-2:2001** |
| Coliform | **Negative** | | **ISO 4832:2006** |
| Salmonella | **Negative** | | **UNI EN ISO 6579: 2000** |
| StaphylococcusAureus | **Negative** | | **UNI EN ISO 68 88-2:2004** |
| Streptococci | **Negative** | | **PT BAT26 rev0 02012** |
| **PRODUCT TREATMENT** | | | |
| Drying Method | **Spray dry** | |  |
