# Supplementary material for: Randomized Clinical Trial: Bergamot Citrus and Wild Cardoon Reduce Liver Steatosis and Body Weight in Non-diabetic Individuals Aged Over 50 Years
Source: Front Endocrinol (Lausanne). 2020 Aug 11;11:494. doi: 10.3389/fendo.2020.00494 (PMC7431622; doi:10.3389/fendo.2020.00494)
Supplement: Supplementary file 1 [file Data_Sheet_1.zip › Table 1.docx]

| **Table 3***. Changes in clinical parameters at follow-up according to the treatments (unpaired t test)* | | | |
| --- | --- | --- | --- |
| **Variables** | **Placebo (n=41)** | **BC (n=45)** | ***p-value*** |
| Follow-up duration (days) | 88±4 | 88±6 | 0.94 |
| Adherence to treatment (≥ 80 %, %) | 92 | 92 | 1 |
| Weight (Kg) | -2.7±2 | -4.2±3 | 0.004 |
| BMI (Kg/m^2^) | -0.9±0.8 | -1.6±0.9 | 0.003 |
| WC (cm) | -3.7±4 | -4.9±4 | 0.16 |
| HC (cm) | -3.2±3 | -3.2±4 | 0.99 |
| FM (kg) | -2.4±2 | -3.3±3 | 0.07 |
| CAP score (dB/m) | -26.9±43 | -48.2±39 | 0.020 |
| Stiffness (kPa) | -0.46±1.4 | -0.26±1.3 | 0.49 |
| Glucose (mg/dL) | -0.07±7 | -0.3±10 | 0.91 |
| Insulin (mU/L) | -1.2±3 | -1.9±5 | 0.41 |
| HOMA-IR | -0.2±0.8 | -0.4±1 | 0.43 |
| TC (mg/dL) | -5.2±32 | -11.9±21 | 0.26 |
| TG (mg/dL) | -2.2±40 | -1.0±34 | 0.85 |
| HDL-C (mg/dL) | -0.5±6 | -2.8±6 | 0.06 |
| LDL-C (mg/dL) | -4.3±28 | -8.9±18 | 0.36 |
| Non-HDL-C (mg/dL) | -4.3±27 | -9.1±19 | 0.42 |
| AST (IU/L) | -0.9±7 | -1.8±5 | 0.48 |
| ALT (IU/L) | -3.5±28 | -2.0±17 | 0.76 |
| γGT (UI/L) | -2.6±9 | -3.8±9 | 0.55 |
| Uric Acid (mg/dL) | 0.13±0.8 | -0.09±0.7 | 0.18 |
| Total bilirubin (mg/dL) | 0.07±0.2 | 0.01±0.3 | 0.22 |
| WBCs (x10ˆ3/uL) | -0.37±1.3 | -0.35±1.3 | 0.94 |
| Lymphocyte (x10ˆ3/uL) | 0.04±0.4 | -0.07±0.5 | 0.36 |
| Neutrophil (x10ˆ3/uL) | -0.31±1.2 | -0.11±1.0 | 0.50 |
| Monocyte (x10ˆ3/uL) | -0.01±0.1 | -0.01±0.1 | 0.97 |
| IL-1β (pg/mL) | -1.56±0.8 | -1.36±0.9 | 0.29 |
| IL-6 (pg/mL) | 0.42±0.5 | 0.48±0.4 | 0.56 |
| TNF-α (pg/mL) | -1.73±2.1 | -1.52±2.9 | 0.71 |
| ***Note.*** BMI = body mass index, WC = waist circumference, HC = hip circumference, FM = fat mass, CAP = controlled attenuation parameter, HOMA-IR = homeostatic model assessment of insulin resistance, TC = total cholesterol, TG = triglycerides, HDL-C = high density lipoprotein cholesterol, LDL-C = low density lipoprotein cholesterol, AST = aspartate aminotransferase, ALT = alanine aminotransferase, γGT = gamma glutamyltransferase, WBCs = white blood cells, IL-1β = interleukin-1β, IL-6 = interleukin-6, TNF-α = tumor necrosis factor α. | | | |
