# Supplementary material for: Randomized Clinical Trial: Bergamot Citrus and Wild Cardoon Reduce Liver Steatosis and Body Weight in Non-diabetic Individuals Aged Over 50 Years
Source: Front Endocrinol (Lausanne). 2020 Aug 11;11:494. doi: 10.3389/fendo.2020.00494 (PMC7431622; doi:10.3389/fendo.2020.00494)
Supplement: Supplementary file 1 [file Data_Sheet_1.zip › Table 3.docx]

| **Table 3.** *Changes in clinical parameters in the subgroups according to the treatments* | | | | |
| --- | --- | --- | --- | --- |
| **ITT** | | | | |
| Variables | Placebo (n=41) | | BC (n=45) | *p-value* |
| Weight (Kg) | -2.7±2 | | -4.2±3 | 0.004 |
| CAP score (dB/m) | -26.9±43 | | -48.2±39 | 0.020 |
| CAP (%) | -9.2±16 | | -15.9±13 | 0.036 |
| Improvement (%) | 51 | | 76 | 0.025 |
| **On-Treatment** | | | | |
| Variables | Placebo (n=38) | | BC (n=42) | *p-value* |
| Weight (Kg) | -2.7±2 | | -4.4±3 | 0.002 |
| CAP score (dB/m) | -25.7±45 | | -51.7±38 | 0.007 |
| CAP (%) | -8.7±17 | | -17.1±12 | 0.013 |
| Improvement (%) | 50 | | 79 | 0.010 |
| **Women** | | | | |
| Variables | Placebo (n=16) | | BC (n=18) | *p-value* |
| Weight (Kg) | -2.6±2 | | -4.3±3 | 0.063 |
| CAP score (dB/m) | -22.3±47 | | -54.1±35 | 0.034 |
| CAP (%) | -8±18 | | -17.8±10 | 0.067 |
| Improvement (%) | 56 | | 83 | 0.13 |
| **Men** | | | | |
| Variables | Placebo (n=25) | | BC (n=27) | *p-value* |
| Weight (Kg) | -2.8±2 | | -4.2±3 | 0.030 |
| CAP score (dB/m) | -29.9±42 | | -44.3±42 | 0.225 |
| CAP (%) | -9.9±15 | | -14.7±14 | 0.249 |
| Improvement (%) | 48 | | 70 | 0.15 |
| **Age ≤ 50 years** | | | | |
| Variables | Placebo (n=21) | BC (n=22) | | *p-value* |
| Weight (Kg) | -2.9±2 | -4.1±3 | | 0.17 |
| CAP score (dB/m) | -43.4±37 | -39.5±43 | | 0.75 |
| CAP (%) | -15.7±13 | -13.9±15 | | 0.67 |
| Improvement (%) | 67 | 73 | | 0.74 |
| **Age > 50 years** | | | | |
| Variables | Placebo (n=20) | | BC (n=23) | *p-value* |
| Weight (Kg) | -2.4±2 | | -4.4±2 | 0.004 |
| CAP score (dB/m) | -9.7±44 | | -56.5±34 | <0.001 |
| *a*CAP score (dB/m)* | -13.7±9 | | -52.9±8 | 0.004 |
| CAP (%) | -2.3±16 | | -17.9±10 | 0.001 |
| Improvement (%) | 35 | | 78 | 0.006 |
| **Android Obesity** | | | | |
| Variables | Placebo (n=27) | | BC (n=35) | *p-value* |
| Weight (Kg) | -2.8±3 | | -4.4±3 | 0.016 |
| CAP score (dB/m) | -23.7±45 | | -51.2±39 | 0.014 |
| CAP (%) | -8.1±17 | | -16.8±13 | 0.027 |
| Improvement (%) | 44 | | 80 | 0.007 |
| **Gynoid Obesity** | | | | |
| Variables | Placebo (n=14) | | BC (n=10) | *p-value* |
| Weight (Kg) | -2.5±3 | | -3.8±3 | 0.22 |
| CAP score (dB/m) | -33.3±41 | | -37.6±41 | 0.80 |
| CAP (%) | -11.4±16 | | -12.9±14 | 0.80 |
| Improvement (%) | 64 | | 60 | 1 |
| **Metabolic Syndrome** | | | | |
| Variables | Placebo (n=11) | | BC (n=9) | *p-value* |
| Weight (Kg) | -2.8±2 | | -3.4±2 | 0.43 |
| CAP score (dB/m) | -9.1±54 | | -47±54 | 0.13 |
| CAP (%) | -2.3±20 | | -14.2±16 | 0.16 |
| Improvement (%) | 55 | | 45 | 0.65 |
| **Overweight & Obesity** | | | | |
| Variables | Placebo (n=32) | | BC (n=40) | *p-value* |
| Weight (Kg) | -3.0±2 | | -4.5±3 | 0.005 |
| CAP score (dB/m) | -25.6±46 | | -50.7±40 | 0.018 |
| CAP (%) | -8.4±17 | | -16.5±18 | 0.031 |
| Improvement (%) | 44 | | 78 | 0.007 |
| ***Note.*** CAP = controlled attenuation parameter. *CAP score adjusted for body weight change. | | | | |
