# Supplementary figures and images for: Randomized Clinical Trial: Bergamot Citrus and Wild Cardoon Reduce Liver Steatosis and Body Weight in Non-diabetic Individuals Aged Over 50 Years
Source: Front Endocrinol (Lausanne). 2020 Aug 11;11:494. doi: 10.3389/fendo.2020.00494 (PMC7431622; doi:10.3389/fendo.2020.00494)

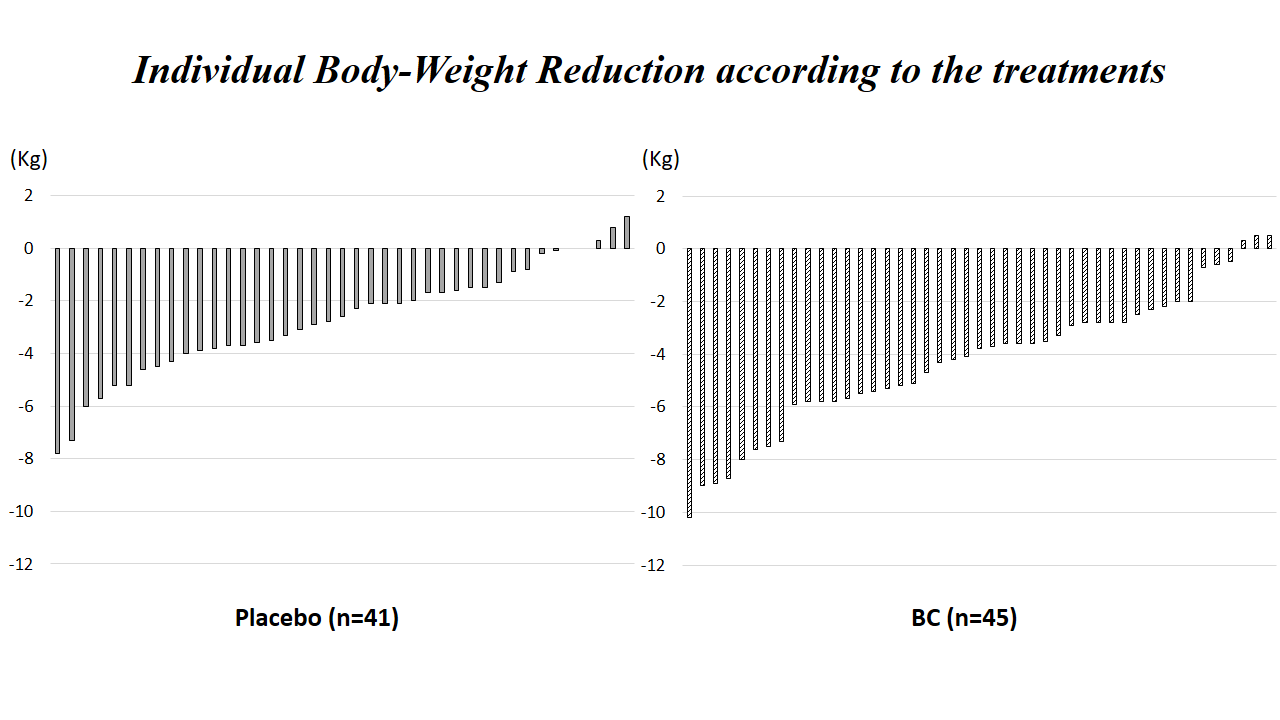

Supplement: Supplementary file 1 [file Data_Sheet_1.zip › Supplemental Figure 2.TIF]

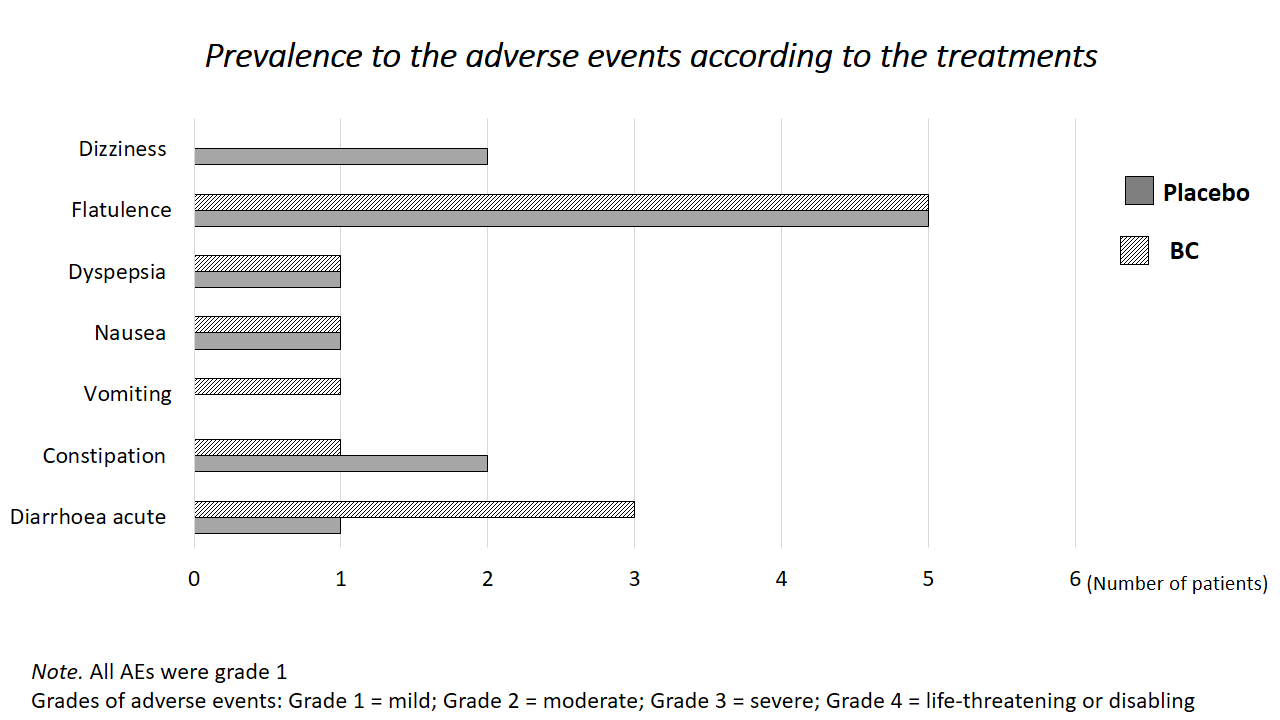

Supplement: Supplementary file 1 [file Data_Sheet_1.zip › Supplemental Figure 3.tif]
